# Sex ratio and age of onset in AQP4 antibody-associated NMOSD: a review and meta-analysis

**DOI:** 10.1007/s00415-024-12452-8

**Published:** 2024-07-03

**Authors:** Simon Arnett, Sin Hong Chew, Unnah Leitner, Jyh Yung Hor, Friedemann Paul, Michael R. Yeaman, Michael Levy, Brian G. Weinshenker, Brenda L. Banwell, Kazuo Fujihara, Hesham Abboud, Irena Dujmovic Basuroski, Georgina Arrambide, Veronika E. Neubrand, Chao Quan, Esther Melamed, Jacqueline Palace, Jing Sun, Nasrin Asgari, Simon A. Broadley, the Guthy Jackson International Clinical Consortium*, Hesham Abboud, Orhan Aktas, Raed Alroughani, Ayse Altintas, Metha Apiwattannakul, Georgina Arrambide, Jagannadha Avasarala, Brenda Banwell, Terrence F. Blaschke, James Bowen, Edgar Carnero Contentti, Tanuja Chitnis, Jerome de Seze, Guillermo Delgado-Garcia, Irena Dujmovic Basuroski, Jose Flores, Kazuo Fujihara, Lorna Galleguillos, Benjamin M. Greenberg, May Han, Joachim Havla, Kerstin Hellwig, Jyh Yung Hor, Sven Jarius, Jorge Andres Jimenez, Najib Kissani, Ingo Kleiter, Marco Lana-Peixoto, M. Isabel Leite, Michael Levy, Sara Mariotto, Maureen A. Mealy, Veronika E. Neubrand, Celia Oreja-Guevara, Lekha Pandit, Sarah M. Planchon, Anne-Katrin Pröbstel, Peiqing Qian, Chao Quan, Pavle Repovic, Claire Riley, Marius Ringelstein, Juan I.Rojas, Dalia Rotstein, Klemens Ruprecht, Maria José Sá, Albert Saiz, Sara Salama, Sasitorn Siritho, Aksel Siva, Terry J. Smith, Elias S. Sotirchos, Ibis Soto de Castillo, Silvia Tenembaum, Pablo Villoslada, Barbara Willekens, Dean Wingerchuk, Bassem I. Yamout, Michael Yeaman

**Affiliations:** 1 School of Medicine and Dentistry, Gold Coast Campus, Griffith University, Gold Coast, QLD 4222 Australia; 2 Department of Neurology, Gold Coast University Hospital, Southport, QLD Australia; 3 Department of Neurology, Penang General Hospital, George Town, Penang Malaysia; 4 NeuroCure Clinical Research Center, Charité - Universitätsmedizin Berlin, corporate member of Freie Universität Berlin, Humboldt-Universität Zu Berlin, and Berlin Institute of Health, Berlin, Germany; 5 Experimental and Clinical Research Center, Max Delbrueck Center for Molecular Medicine and Charité - Universitätsmedizin Berlin, Berlin, Germany; 6 Department of Medicine, David Geffen School of Medicine at the University of California, Los Angeles, CA USA; 7 Department of Medicine, Divisions of Molecular Medicine & Infectious Diseases, Harbor-UCLA Medical Center, Torrance, CA USA; 8 Lundquist Institute for Biomedical Innovation at Harbor-UCLA Medical Center, Torrance, CA USA; 9 Department of Neurology, Massachusetts General Hospital and Harvard Medical School, Boston, MA USA; 10 Department of Neurology, University of Virginia, Charlottesville, VA USA; 11 Division of Child Neurology, Children’s Hospital of Philadelphia, Department of Neurology and Department of Pediatrics, Perelman School of Medicine, University of Pennsylvania, Philadelphia, PA USA; 12 Department of Multiple Sclerosis Therapeutics, Fukushima Medical University and Multiple Sclerosis and Neuromyelitis Optica Center, Southern Tohoku Research Institute for Neuroscience, Koriyama, Japan; 13 Case Western Reserve University, University Hospitals Cleveland Medical Center, Cleveland, OH USA; 14 Department of Neurology, University of North Carolina School of Medicine, Chapel Hill, NC USA; 15 Neurology-Neuroimmunology Department, Multiple Sclerosis Centre of Catalonia (Cemcat), Vall d’Hebron Barcelona Hospital Campus, Barcelona, Catalonia Spain; 16 Department of Cell Biology, Faculty of Sciences, University of Granada, Granada, Spain; 17 Department of Neurology, The National Centre for Neurological Disorders, Huashan Hospital, Shanghai Medical College, Fudan University, Shanghai, China; 18 Dell Medical School, University of Texas, Austin, TX USA; 19 Nuffield Department of Clinical Neurosciences, Oxford University Hospitals, Oxford, UK; 20 Department Clinical Neurology, John Radcliffe Hospital, Oxford, OX3 9DU UK; 21 Institute of Integrated Intelligence and Systems, Nathan Campus, Griffith University, Nathan, QLD Australia; 22 Rural Health Research Institute, Charles Sturt University, Bathurst, NSW Australia; 23 Department of Neurology, Slagelse Hospital, Slagelse, Denmark; 24 Institutes of Regional Health Research and Molecular Medicine, University of Southern Denmark, Odense, Denmark

**Keywords:** Neuromyelitis optica, Risk factors, Environment, Aetiology, Epidemiology, Age of onset, Sex

## Abstract

**Background:**

Aquaporin-4 (AQP4) antibody-associated neuromyelitis optica spectrum disorder (NMOSD) is an antibody-mediated inflammatory disease of the central nervous system. We have undertaken a systematic review and meta-analysis to ascertain the sex ratio and mean age of onset for AQP4 antibody associated NMOSD. We have also explored factors that impact on these demographic data.

**Methods:**

A systematic search of databases was conducted according to the PRISMA guidelines. Articles reporting sex distribution and age of onset for AQP4 antibody-associated NMSOD were reviewed. An initially inclusive approach involving exploration with regression meta-analysis was followed by an analysis of just AQP4 antibody positive cases.

**Results:**

A total of 528 articles were screened to yield 89 articles covering 19,415 individuals from 88 population samples. The female:male sex ratio was significantly influenced by the proportion of AQP4 antibody positive cases in the samples studied (*p* < 0.001). For AQP4 antibody-positive cases the overall estimate of the sex ratio was 8.89 (95% CI 7.78–10.15). For paediatric populations the estimate was 5.68 (95% CI 4.01–8.03) and for late-onset cases, it was 5.48 (95% CI 4.10–7.33). The mean age of onset was significantly associated with the mean life expectancy of the population sampled (*p* < 0.001). The mean age of onset for AQP4 antibody-positive cases in long-lived populations was 41.7 years versus 33.3 years in the remainder.

**Conclusions:**

The female:male sex ratio and the mean age of onset of AQP4 antibody-associated NMOSD are significantly higher than MS. The sex ratio increases with the proportion of cases that are positive for AQP4 antibodies and the mean age of onset increases with population life expectancy.

**Supplementary Information:**

The online version contains supplementary material available at 10.1007/s00415-024-12452-8.

## Introduction

Aquaporin-4 (AQP4) antibody-associated neuromyelitis optica spectrum disorder (NMOSD) is an antibody-mediated autoimmune astrocytopathy that typically manifests as symptoms arising from inflammatory attacks of the optic nerves, diencephalon, periaqueductal grey matter, and spinal cord [1]. Involvement of the area postrema, periependymal brainstem, hypothalamus, and cerebral hemispheres also occurs [1]. Serum antibodies to AQP4, a water channel found in high density on the foot processes of astrocytes at the blood–brain barrier, appear to be pathogenic [2]. Without treatment, the condition is associated with significant morbidity and mortality [3].

Little is known about the aetiology of NMOSD, but studies suggest that genetic and environmental factors for MS are not shared by AQP4 antibody-associated NMOSD [4–6]. Epidemiological studies are an essential step in gaining clues to aetiology [7]. Recent systematic reviews of the worldwide distribution of NMOSD have indicated that the condition appears to occur in people of all ethnic backgrounds, but that prevalence is highest in Black Africans (ninefold), and is higher in South-East Asians (threefold), when compared to populations with European Ancestry [8].

Prior studies have indicated that the sex ratio (female:male) is higher in NMOSD than for MS, but some variability of results has been noted [9]. This has been attributed to possible geographical variability or differing diagnostic criteria [10]. Several studies have also noted a different sex ratio in paediatric and late onset (typically defined as an onset age of 50 years or higher) [11, 12]. The age of onset in NMOSD has been noted to be higher than in MS [10]. The diagnostic criteria for NMOSD have changed over the past 25 years [1, 13–15] and the emergence of other antibody-mediated demyelinating diseases of the CNS, that have an overlapping clinical picture, such as myelin oligodendrocyte glycoprotein antibody-associated disease (MOGAD) [16] which has a female:male sex ratio approaching 1.0 and mean age of onset similar to or possibly younger than MS [17], has complicated the task of defining seronegative NMOSD.

Here we have undertaken a systematic review and meta-analysis of studies reporting on the sex and age of onset distribution for NMOSD. The aim of the study was to define the sex and age of onset distribution around the world and explore potential associations with ethnicity and population structure. We have initially undertaken a more inclusive approach using various diagnostic criteria to provide a wider geographical range of surveys, before conducting a more focused analysis of studies looking at only AQP4 antibody positive cases. We would emphasise that all included studies were surveying what at the time was thought to be AQP4 antibody-associated NMOSD although it is now recognised that the earlier criteria are likely to have included a heterogeneous group of diagnoses including MOGAD and MS. We hypothesised that in AQP4 antibody associated NMOSD: (1) the proportion of females would be higher than is seen in multiple sclerosis (MS); (2) the proportion of females would be the same in different populations; and (3) the mean age of onset would be different in different parts of the world depending upon mean life expectancy and ethnicity.

## Methods

### Literature search

Two independent searches of Medline, Embase and PubMed databases using the following search strings: (1) (“neuromyelitis optica” OR “NMO” OR “NMOSD” OR “Devic’s disease” AND “sex”); and (2) (“neuromyelitis optica” OR “NMO” OR “NMOSD” OR “Devic’s disease” AND “age of onset” OR “age at onset”) restricted to articles in English and published from 1 January 1999 to 17 July 2023 were undertaken. Articles were screened based on title and abstract looking for articles reporting on epidemiological studies of people with NMOSD assumed to be related to AQP4 antibodies. Full text articles were reviewed according to the following criteria. Inclusion criteria were: population-based or clinic-based surveys; incidence, prevalence, cross-sectional, cohort or case–control studies; diagnosis of AQP4 antibody related NMO or NMOSD as determined by the relevant diagnostic criteria of the time; and reporting data on sex or age of onset in any format. Exclusion criteria were: article not in English; selected population (e.g. on treatment); lack of raw or summary data; data subsequently updated; earlier more comprehensive study; review article; not NMOSD population; and case reports. Studies looking at whole population data and specific age groups (e.g. paediatric or late onset) were analysed separately for sex distribution. Age-specific groups were not included in the age-of-onset analysis.

### Data extraction

The following data were extracted: first author; year of publication; prevalence year or last year of data collection; geographical location; study design (prevalence study or case series); setting; recruitment source (population-based or clinic-based); age cut-offs for paediatric and late-onset cohorts; ethnicity; diagnostic criteria used; AQP4 assay used; proportion of AQP4 antibody positive cases (of all cases not just those tested); size of study population; number of females and males (as assigned at birth); mean or median age of onset (years) with standard deviation, quartiles or range; and reason for exclusion (if excluded). Where age of onset distribution data was provided this was tabulated into decade age ranges. Mean life expectancy has been used as a simple measure of population age distribution, with a lower mean life expectancy indicating a downshifted, population age distribution profile (fewer people living to an older age) [18]. Thus, a lower mean life expectancy indicates that a smaller proportion of the population will have survived to acquire any given disease at an older age, thereby resulting in a lower mean age of onset for that disease. Mean life expectancy for females in the country of study in 2019 from the World Health Organisation website was used [19]. For multinational studies, life expectancy for the country with the largest proportion of cases was used.

Study quality was assessed using the Joanna Briggs Institute (JBI) critical appraisal checklist for prevalence studies [20]. For the initial analysis, all studies were included, but studies assessed as “No” or “Unclear” for any item on the JBI prevalence checklist (except estimation of response rates) were removed as part of sensitivity analyses. Estimation of response rates was deemed to be of less importance in the current context, as compared to a prevalence study. Review of studies and quality assessment were conducted by two researchers, any discrepancies were resolved by a third. This study used only previously published summary data and therefore institutional ethics review and participant consent was not required.

### Statistical analysis

Where the age of onset data were presented as median and range or quartiles, appropriate methods were used to estimate the mean and standard deviation [21]. The sex ratio is reported as the female:male ratio. Meta-regression analysis was performed using the Comprehensive Meta-analysis v3 (Biostat^®^ Inc., Englewood, NJ, US) statistical package [22]. The proportion of AQP4 antibody positive cases (> 90% or ≤ 90%), life expectancy (< 80 years or ≥ 80 years), mean age of onset (> 35 years or ≤ 35 years) and geographical location were included as covariates as appropriate. Zero counts for the sex distribution data were adjusted by the addition of 1 to both the number of males and females [23]. The natural log scale was used for sex ratio Forest plots. Fixed effects or random effects models were used as appropriate for the observed heterogeneity using *I*^2^ and *Tau*^2^ [24]. The risk of bias was assessed using Funnel Plots and Egger’s test [25]. Sensitivity analyses restricted to studies using cell-based assays for AQP4 antibodies were undertaken and *p* < 0.05 was considered significant. Age standardisation [26] was applied to the age of onset distribution data using the population distribution for each country in the year of study from the Population Pyramid website [27].

## Results

### Literature search

**Fig. 1:**
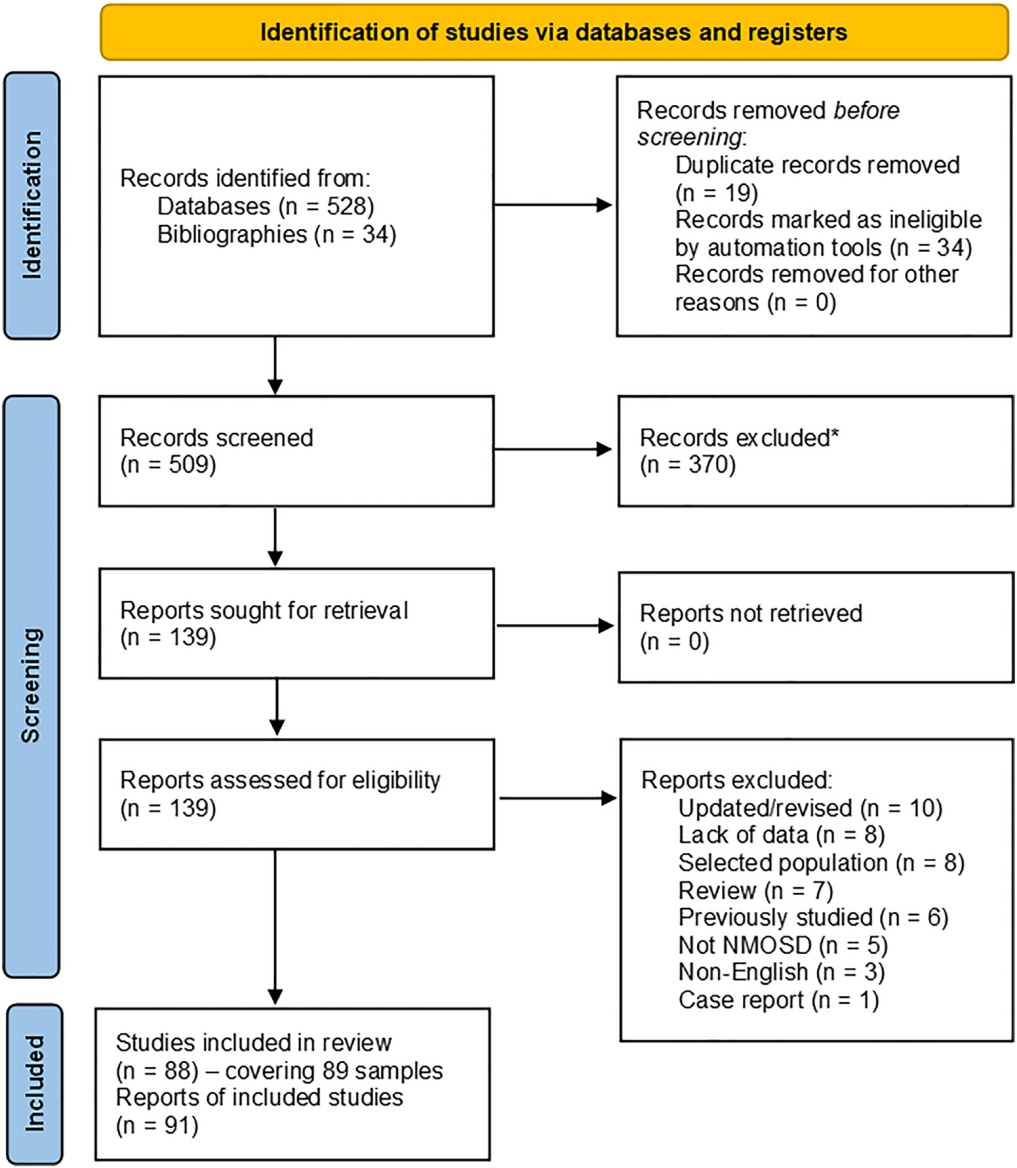
PRISMA flow chart of outcomes from literature review. NMOSD = neuromyelitis optica spectrum disorder

From a total of 562 potential articles, 88 articles were included (64 whole population/adult studies [3, 28–90], 13 paediatric studies [11, 91–102] and 11 late onset studies [12, 103–112]) as shown in Fig. 1. Two of the whole population studies reported on two separate subpopulations which were analysed separately [50, 74]. Three studies had relevant updates or prior studies that provided additional relevant information regarding AQP4 antibody positive cases and were therefore included [113–115]. Thus, there were a total of 91 articles covering 89 separate populations. Study quality varied. Few studies included an estimate of the number of missed cases [28, 37, 86, 113]. Some studies lacked details about the sampling process, particularly in relation to the timing of the data collection and the type of AQP4 assay used. A summary of all included studies reviewed, together with a summary of the critical appraisal, is given in Supplementary Table S1. Excluded studies and reasons for exclusion are listed in Supplementary Table S2 [8, 9, 116–161]. There were nine studies that provided age of onset distribution data (Supplementary Table S3) [29, 36, 37, 39, 43, 47, 72, 82, 87] with one providing data for three separate years of onset data [87].

### Sex distribution

#### Whole population studies

**Fig. 2:**
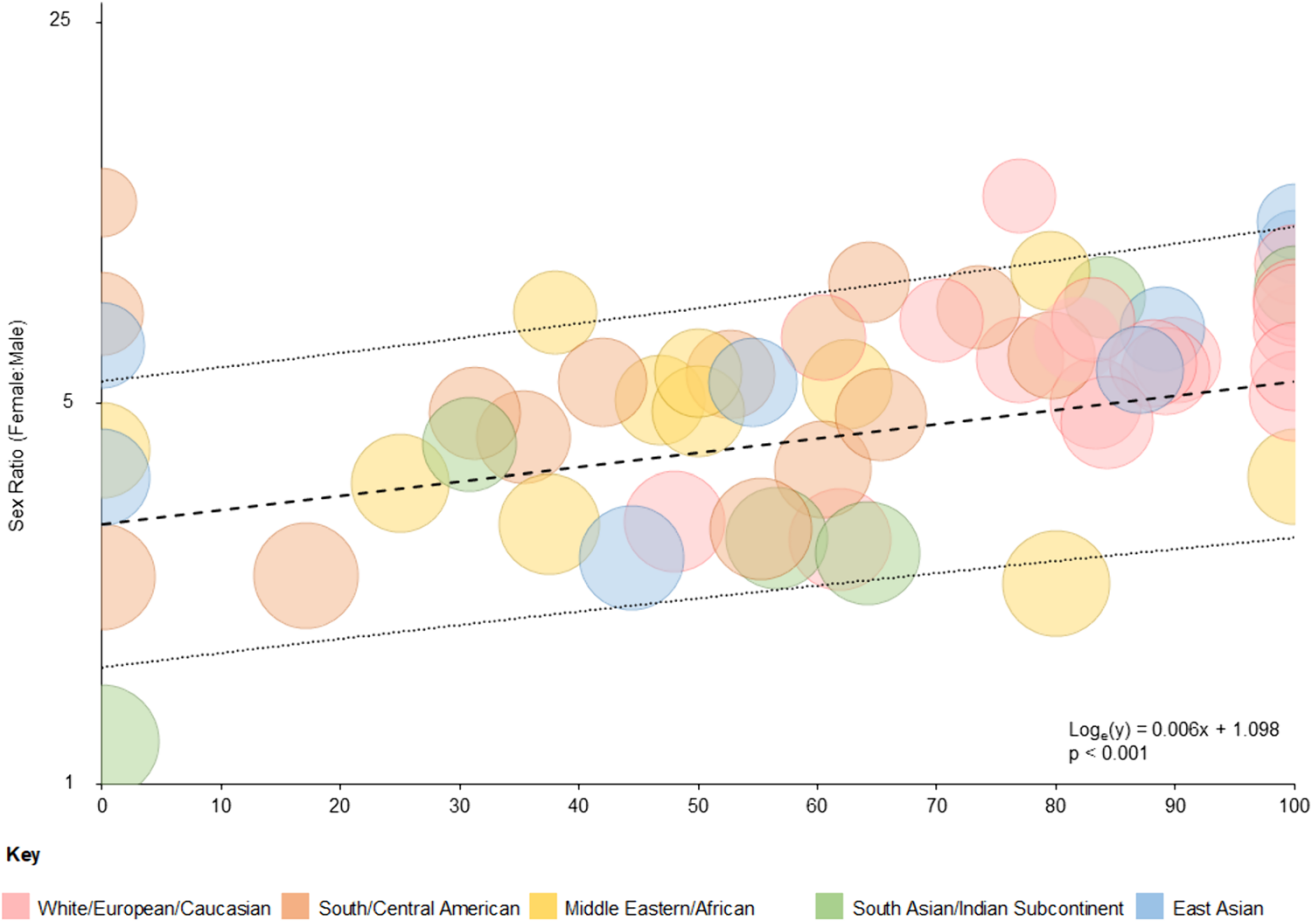
Bubble plot of sex ratio for NMOSD studies plotted against the proportion of AQP4 antibody positive cases in the sample studied. The sex ratio is plotted on a log scale. Bubble size is proportional to the variance of the sex ratio. Meta-regression model included geographical regions. The dashed line indicates fitted regression from meta-regression analysis (indicated by formula) and dotted lines show a 95% confidence interval

**Fig. 3:**
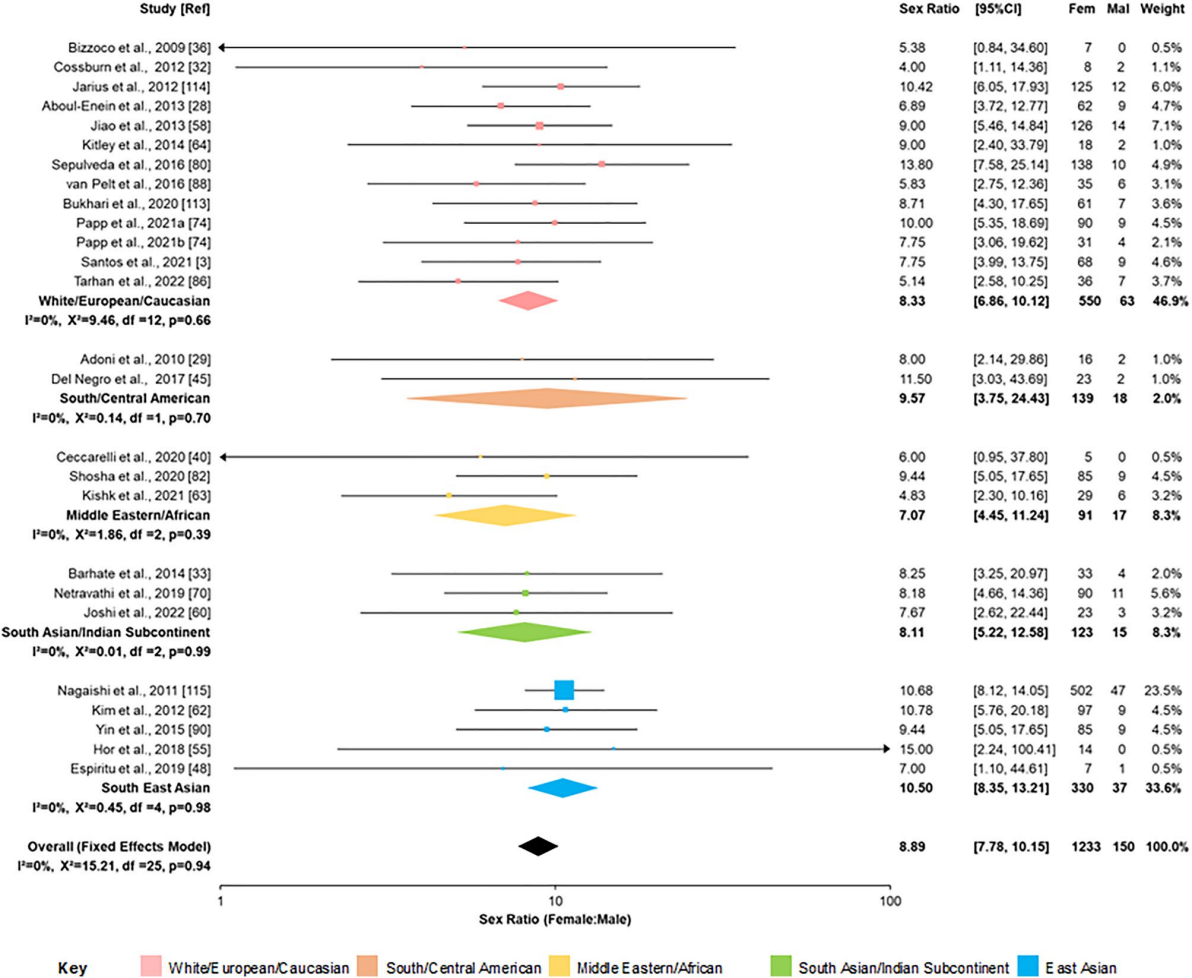
Forest plot of sex ratio, sub-grouped by geographical region for studies only including AQP4 antibody positive cases. Fixed effects model. Plotted on log scale. Colour coding of geographical regions is indicated by the key. CI = confidence interval; Fem = female; Mal = male

Meta-analysis for whole population studies (*N* = 19,415) grouped by geographical region (Supplementary Fig. S1) gave an overall sex ratio of 4.70 (95% CI 4.33–5.11). Superficially, this analysis suggests that the female:male sex ratio in Europe and East Asia may be higher, and may be lower in the Indian Subcontinent. However, there was considerable heterogeneity (*I*^*2*^ = 61%). Meta-regression analysis including the proportion of AQP4 antibody-positive cases and geographical location (*N* = 6,917) revealed a significant residual heterogeneity and that the sex ratio was significantly influenced by the proportion of AQP4 antibody-positive cases (Supplementary Table S4) but not by geographical location. The diagnostic criteria used had no effect once the proportion of AQP4 antibody cases was taken into account (data not shown). A higher proportion of AQP4 antibody positive cases was associated with a higher sex ratio (*p* < 0.001) and this is illustrated in a bubble chart (Fig. 2). Funnel plot (Supplementary Fig. S2A) and Egger’s test (*p* = 0.21) indicated no publication bias. When analysis was restricted to studies with only AQP4 antibody-positive cases (Fig. 3), *I*^2^ was reduced to 0% indicative of very low residual heterogeneity (*p* = 0.53) and gave a female:male ratio of 8.89 (95% CI 7.78–10.15) equivalent to 90% being female. Sensitivity analyses, restricted to studies using a cell-based assay (sex ratio = 8.87 [95% CI 7.14–11.03]) or restricting to the 13 studies meeting all JBI critical appraisal domains (except for response rate) in AQP4 antibody positive cases (sex ratio = 8.50 [95% CI 7.01–10.31]) did not significantly affect this finding.

#### Paediatric and late-onset studies

**Fig. 4:**
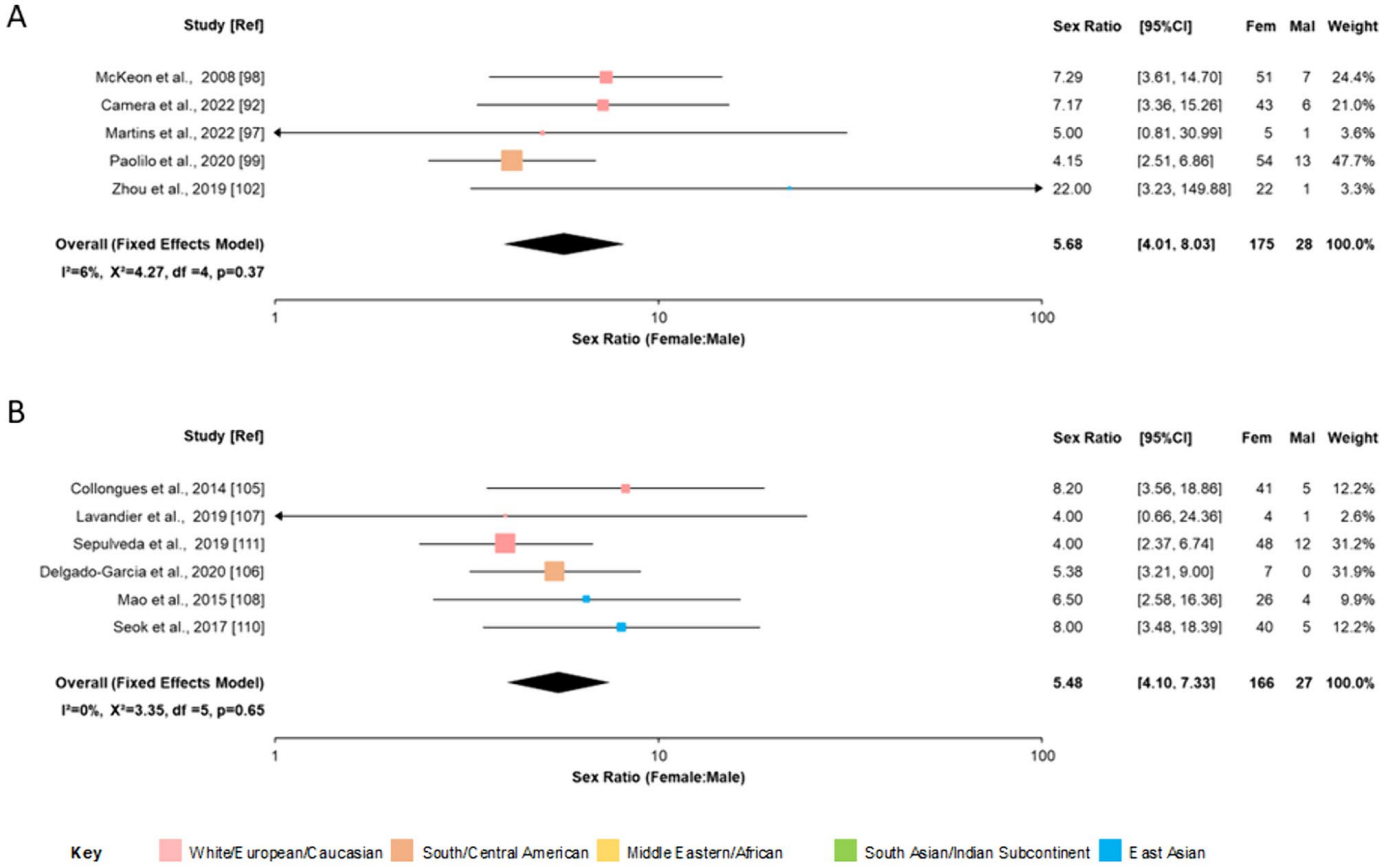
Forest plots of sex ratio in paediatric (**A**) and late-onset (**B**) AQP4 antibody positive cases of NMOSD. Inverse variance method with fixed effects models. Colour coding of geographical regions is indicated by the key. CI = confidence interval

Data for all studies is given in Supplementary Fig. S3. Because of the above-noted effects of AQP4 antibody status on sex distribution, only data for seropositive cases were analysed (five paediatric and six late-onset studies). The overall estimate for the sex ratio in paediatric studies (*N* = 203) was 5.68 (95% CI 4.01–8.03). These confidence intervals overlap with those of the whole population studies (Fig. 4a). The sex ratio in late-onset cases (*N* = 193) was 5.48 (95% CI 4.10–7.33) as shown in Fig. 4b. Funnel plots (Supplementary Fig. S4) did not suggest any significant publication bias.

### Age of onset

**Fig. 5:**
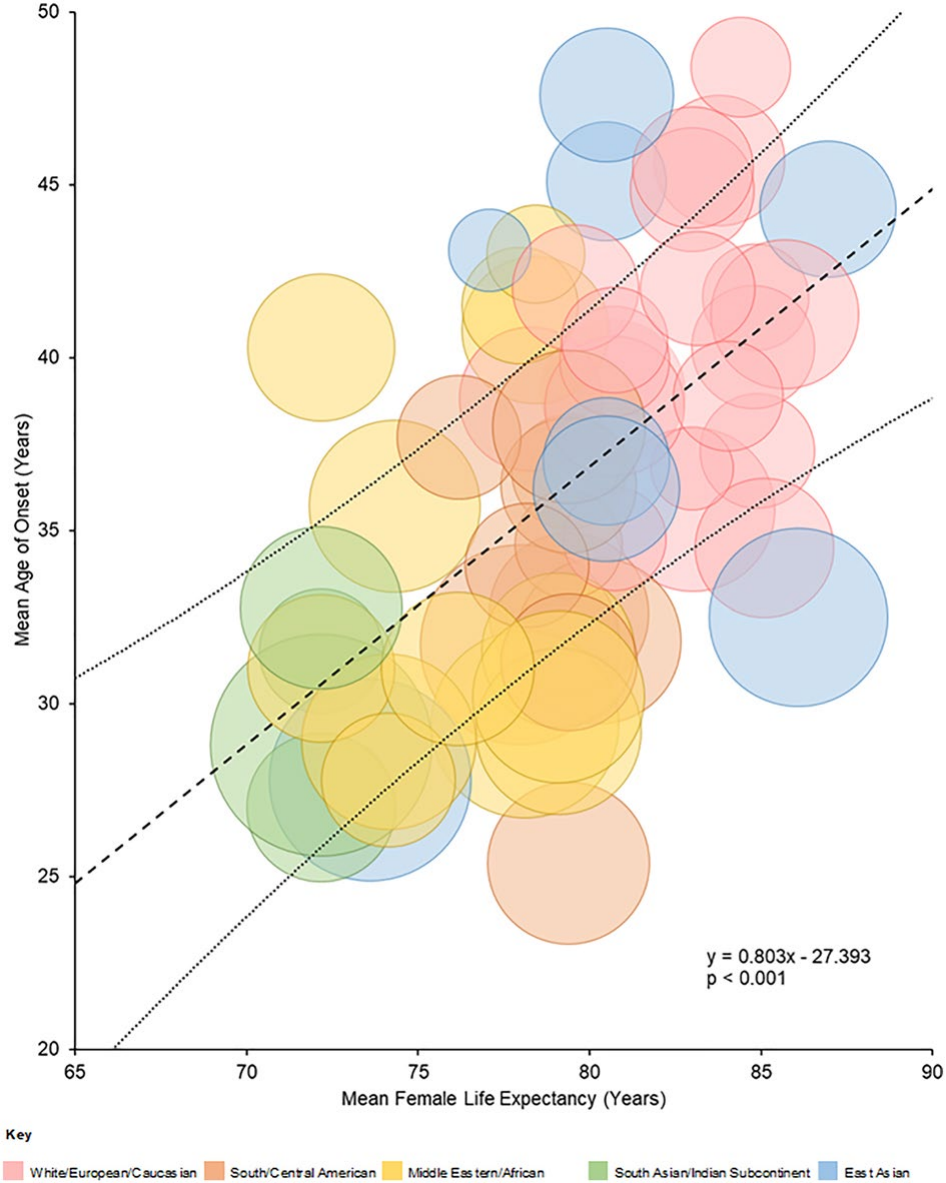
Bubble plot of mean age of onset against mean female, life expectancy for the country of study. Bubble size is proportional to the variance of the mean age of onset. Meta-regression model included the proportion of AQP4 antibody positive cases. The dashed line indicates fitted regression from meta-regression analysis (indicated by formula) and dotted lines show a 95% confidence interval

**Fig. 6:**
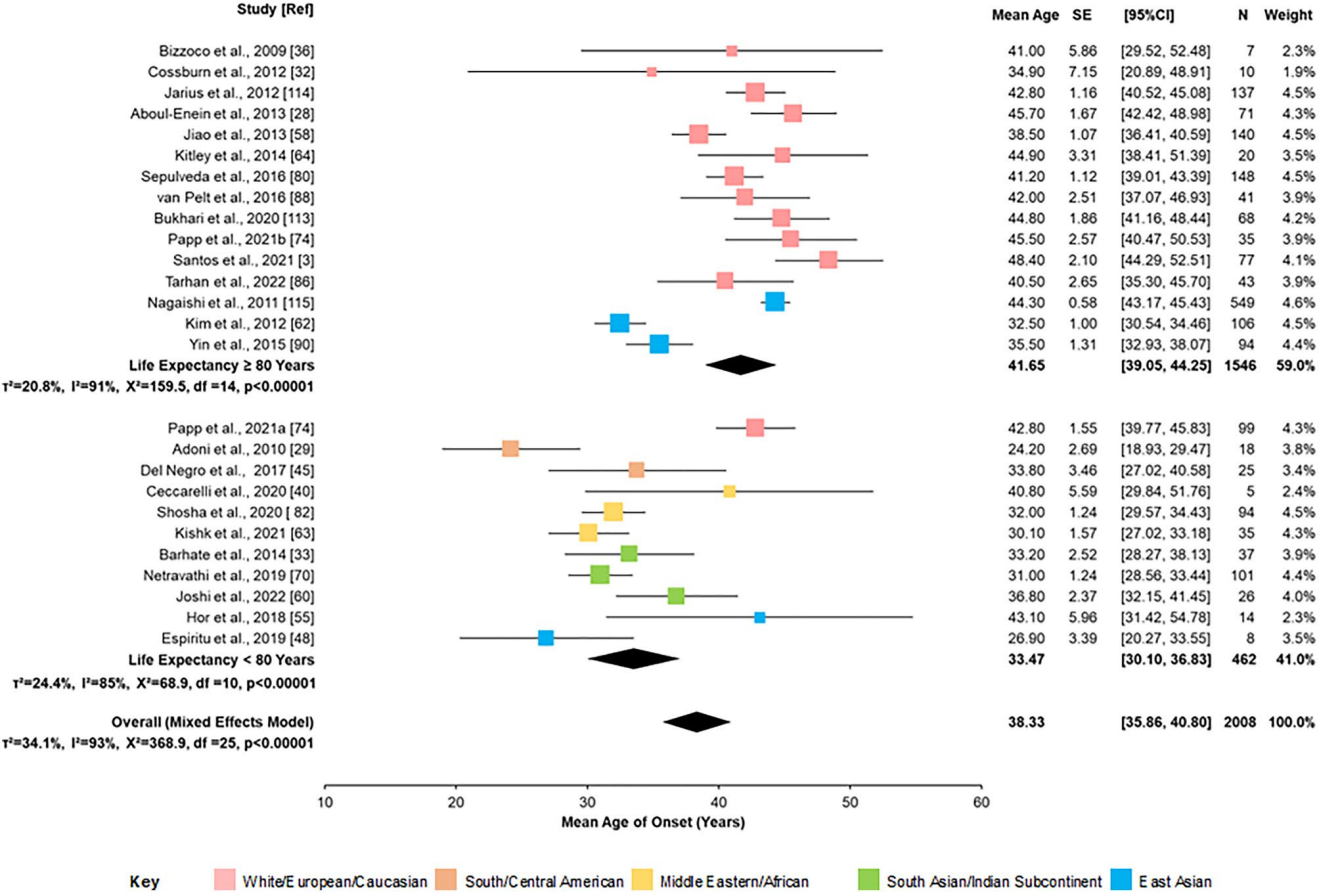
Forest plot of mean age of onset, sub-grouped by mean life expectancy (≥ 80 year or < 80 years) for studies only including AQP4 antibody positive cases. Mixed effects model. Colour coding of geographical regions is indicated by the key. CI = confidence interval; *N* = number of cases

Mean age of onset and standard deviation data were either available or could be calculated for 54 populations covering a total of 6240 cases. The outcome of meta-regression analysis is given in Supplementary Table S5 and indicates that the mean age of onset was significantly influenced by population life expectancy (*p* < 0.001) and proportion of AQP4 antibody positive cases (*p* = 0.019), but not geographical region. These results are illustrated in Fig. 5 and Supplementary Fig. S5. Meta regression analysis restricted to only AQP4 antibody positive populations, stratified by mean female, life expectancy is illustrated in Fig. 6 and summarised in Supplementary Table S6. This analysis showed that the overall mean age of onset was 38.3 years (95% CI 35.9–40.8) and the age of onset ranged from 2 to 86 years. However, an *I*^2^ of 93% and *Tau*^*2*^ of 32.6 indicated a significant degree of heterogeneity (*p* < 0.00001). The mean age of onset for countries where the life expectancy of females was 80 or more years was 41.7 (95% CI 39.1–44.3) and for those where life expectancy was < 80 years was 33.5 (95% CI 30.1–36.8), a difference that was statistically significant (*p* < 0.001). Sensitivity analysis restricting studies meeting all JBI critical appraisal criteria in regions with live expectancy was 80 years or more gave the same result (42.0 years [95% CI 38.3–45.6]). The effect of life expectancy on the mean age of onset within AQP4 antibody positive populations is shown in Supplementary Fig. S6 (*p* < 0.001). Funnel plots (Supplementary Fig. S2B) and Egger’s test (*p* = 0.678) did not suggest any publication bias.

**Fig. 7:**
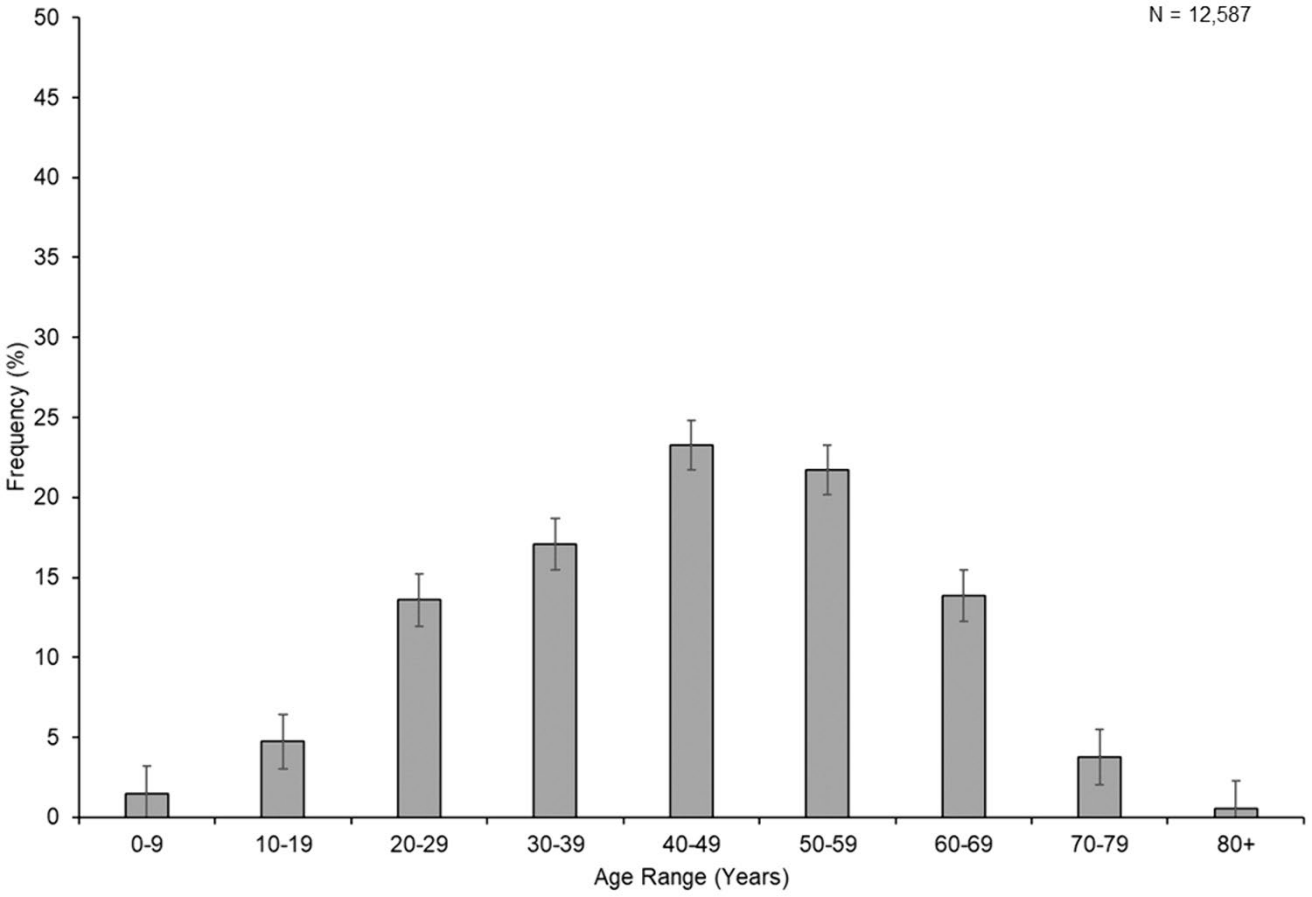
Age of onset distribution by decade for NMOSD. Error bars indicate 95% confidence interval

There were nine studies that provided age of onset data per decade of life [29, 36, 37, 39, 43, 47, 72, 82, 87] with one study giving incidence data for three separate years of data collection [87] which were included as three separate cohorts (Supplementary Table S6). A summation of these data are provided in Fig. 7 (*N* = 12,599). The commonest age of onset was 40–49 years and the age of onset profile was flatter and broader than that seen in MS. Similar to MS, and contrary to MOGAD [16], AQP4 antibody associated NMOSD appears to be relatively uncommon below the age of 10 years. Unlike MS, small numbers of cases continue to occur into the eighth and ninth decades. The double peak of age of onset seen in MS [162] and MOGAD [163] was not evident for AQP4 antibody associated NMOSD. Adjustment of age of onset distribution to a flat age structure (Supplementary Fig. S7A) provides an indication of the relative risk of acquiring AQP4 antibody-associated NMOSD in each decade and suggests that the age range 50–59 may represent the period of greatest risk. Data restricted to AQP4 antibody positive cases only (*N* = 106) are again presented in Supplementary Fig. S7B and is similar to the larger dataset.

## Discussion

This review and meta-analysis have shown that both the sex ratio and mean age of onset for AQP4 antibody-associated NMOSD are significantly influenced by the proportion of seropositive cases and population age profile. The effect of seropositive proportion on age of onset could be due to lower titres of antibody earlier in the disease course (i.e. at a younger age). There was a linear relationship between the sex ratio (plotted on the log scale) and the proportion of AQP4 antibody-positive cases (*p* < 0.001). Studies with lower proportions of AQP4 antibody-positive cases were associated with a lower sex ratio. It is harder to explain the effect of seropositive proportion on the sex ratio, where the sex ratio for false negative cases would be expected to be the same as seropositives. This finding suggests the accidental inclusion of cases with a diagnosis other than AQP4 antibody-associated NMOSD in these cohorts. This is likely to be a heterogenous group and might include MS, which has a lower female:male sex ratio (typically 2.73) [164], MOGAD (sex ratio typically 1.00) [17] or other yet-to-be-defined demyelinating disorders, as well as seronegative NMOSD. These findings are in line with prior studies of AQP4 antibody-associated NMOSD that have shown a lower female:male sex ratio and younger age of onset in seronegative cases when compared to seropositive cases [63, 113, 114].

The effect of population profile on the mean age of onset is also interesting. Populations with age distributions skewed towards younger ages will inevitably have a younger age of onset for any condition where onset spans the full range of ages, such as NMOSD (2–86 years). Mean life expectancy is a simple measure of population distribution [165] and showed a persistent effect on mean age of onset even when restricted to AQP4 antibody-positive cases with a regression coefficient of 0.8 (*p* < 0.001).

Taking these factors into account the female:male sex ratio in NMOSD was 8.89 (95% CI 7.78–10.15) when restricted to AQP4 antibody positive cases. This is significantly higher than the 2.73 (95% CI 2.37–3.09) figure for contemporaneous cohorts of MS [164], as the confidence intervals do not overlap. The female:male predominance for NMOSD is similar to that seen for systemic lupus erythematosus (SLE) 7:1 [166] and Sjögren’s syndrome 14:1 [167], two conditions that have been noted to co-exist in people with NMSOD [168]. This suggests a common pathophysiology or aetiology in which female sex is particularly important. It has been postulated that oestrogen may be of primary importance with a trial of an oestrogen receptor antagonist being of some benefit in SLE [166]. The sex ratio in AQP4 antibody-positive paediatric (5.69 [95% CI 3.45–9.38]) and late onset studies (5.48 [ 95% CI 4.10–7.33) were lower suggesting that the sex distribution of AQP4 antibody-associated NMOSD at the extremes of age may be different. One possible explanation for this may be an oestrogen effect during reproductive years increasing the risk of autoimmune disease in women [169].

The age of onset for AQP4 antibody-associated NMOSD ranged from 2 to 86 years and had a mean of 41.7 years (95% CI 38.5–43.8) when only AQP4 antibody-positive cases from longer-lived populations were considered. This is significantly higher (by almost 10 years) than the figure of 32 years for MS [164], although the latter may be increasing [170]. The distribution of age of onset across more than 12,000 cases shows a flatter profile than MS, with the commonest age of onset being 40–49 years, with a quarter of cases occurring in this decade. The decade of greatest risk per head of population was 50–59 years. The age profile for AQP4 antibody-associated NMOSD also includes approximately 20% of cases that occur in the seventh, eighth, and ninth decades. This contrasts with MS where age of onset greater than 60 years is rare (< 1%) [171, 172]. The flatter age of onset profile for AQP4 antibody-associated NMOSD is consistent with a triggering event that is relatively rare in the general population and shares similarities with Guillain–Barré syndrome [173]. The linear increase in incidence with age up to a peak around the fifth decade and then decrement in frequency at higher ages is consistent with either a latent period following exposure effect or genetic factors, where genetic load is associated with a younger age of onset [174].

This meta-analysis confirms that the female:male sex ratio for AQP4 antibody-associated NMOSD is significantly higher than that seen in MS (8.9:1 vs. 2.7:1). The mean age of onset in AQP4 antibody-associated NMOSD is approximately 10 years higher than is seen in MS or MOGAD. As with MS the number of cases of AQP4 antibody-associated NMOSD occurring before the age of 10 is relatively low [175], and contrasts with the distribution of MOGAD which has a distinct peak in childhood [176]. Finally, the peak age of risk per head of population may be 50–59 years for AQP4 antibody-associated NMOSD.

The strengths of this study were that a systematic approach, using published checklists, duplicate review, and standardised quality assessment tools, was used to undertake the literature search and record the data. The analysis was comprehensive and explored potential confounding factors with an initially inclusive approach, but ultimately more restrictive analysis focused on more homogeneous populations. The final results are robust with narrow confidence intervals and low heterogeneity when the analysis was restricted to seropositive cohorts in longer-lived populations. The potential weaknesses are that individual patient-level data were not generally available and some data (e.g. proportion of AQP4 antibody-positive cases) was not stated for every study. The collection of individual-level data should be considered in future analyses. Life expectancy is an unsophisticated measure of population distribution and unusual patterns of mortality age distributions could have an impact (e.g. high childhood mortality) [165]. Individual level data for the age of onset would permit a stratified analysis based on the population distribution for the region surveyed in each study, allowing calculation of age-specific, incidence rates [26].

This study provides a comprehensive update on the sex distribution and age of onset profile for AQP4 antibody-associated NMOSD that can be used as a benchmark for future comparative studies. The fact that studies using less stringent diagnostic criteria (seronegative cases) gave significantly different estimates of both sex ratio and mean age of onset points to phenotypic heterogeneity. We would recommend that future studies of AQP4 antibody-associated NMOSD report only on AQP4 antibody-positive cases or that data for seropositive cases be reported separately.

### Electronic supplementary material

Below is the link to the electronic supplementary material.

Supplementary file 1 (DOCX 142 kb)

Supplementary file 2 (PDF 1146 kb)

## Data Availability

Full data used in this analysis are available from the corresponding author upon request.

## Appendix 1

### Affiliated members of the Guthy–Jackson charitable foundation international clinical consortium:

Hesham Abboud, MD, PhD, Case Western Reserve University, University Hospitals Cleveland Medical Center Cleveland, Ohio, USA; Orhan Aktas, MD, Heinrich Heine University Düsseldorf, Düsseldorf, Germany; Raed Alroughani, MD, FRCPC, Division of Neurology, Amiri Hospital, Kuwait City, Kuwait; Ayse Altintas, MD, Koc University School of Medicine and Koc University Research Center for Translational Medicine (KUTTAM), Istanbul, Turkey; Metha Apiwattannakul, MD, Neurological Institute of Thailand. Bangkok, Thailand; Georgina Arrambide, MD, PhD, Neurology-Neuroimmunology Department, Multiple Sclerosis Centre of Catalonia (Cemcat), Vall d’Hebron Barcelona Hospital Campus, Barcelona, Catalonia, Spain; Jagannadha Avasarala, MD, PhD, U of Kentucky Medical Center, Lexington, KY, USA; Brenda Banwell, MD, Children's Hospital of Philadelphia, University of Pennsylvania, Philadelphia, PA, USA; Terrence F. Blaschke, MD, Stanford University, Stanford, CA, USA; James Bowen, MD, Multiple Sclerosis Center, Swedish Neuroscience Institute Seattle, WA, USA; Edgar Carnero Contentti, MD, MSc, Department of Neurosciences, Hospital Alemán, Buenos Aires, Argentina; Tanuja Chitnis, MD, Brigham and Women's Hospital, Boston, MA, USA; Jerome de Seze, MD, PhD, University Hospital of Strasbourg, Strasbourg, France; Guillermo Delgado-Garcia, MD, MSc, University of Calgary, Canada; Irena Dujmovic Basuroski, MD, PhD, University of North Carolina School of Medicine Department of Neurology, Chapel Hill, NC, USA; Jose Flores, MD, MSc, National University of México, México City, Mexico; Kazuo Fujihara, MD, Fukushima Medical University, Koriyama, Japan; Lorna Galleguillos, MD, Clínica Alemana de Santiago, Chile; Benjamin M. Greenberg, MD, MHS, University of Texas Southwestern, Dallas, Texas, USA; May Han, MD, Stanford University, Stanford, CA, USA; Joachim Havla, MD, LMU Hospital, Munich, Germany; Kerstin Hellwig, MD, Katholisches Klinikum, Bochum, Germany; Jyh Yung Hor, MD, Penang General Hospital, Penang, Malaysia; Sven Jarius, MD, Universität Heidelberg, Heidelberg, Germany; Jorge Andres Jimenez, MD, Neuroclinica, Medellin, Colombia; Najib Kissani, MD, Neurology Department, Marrakech, Morocco; Ingo Kleiter, MD, Marianne-Strauß-Klinik, Berg, Germany; Marco Lana-Peixoto, MD, PhD, CIEM MS Research Center, Federal University of Minas Gerais, Belo Horizonte, Minas Gerais, Brazil; M. Isabel Leite, MD, DPhil, FRCP, University of Oxford, Oxford, UK; Michael Levy, MD, PhD, Massachusetts General Hospital and Harvard Medical School, Boston, MA USA; Sara Mariotto, MD PhD, Neurology Unit, Department of Neurosciences, Biomedicine, and Movement Sciences; University of Verona, Verona, Italy; Maureen A. Mealy, PhD, Horizon Therapeutics, Rockville, MD, USA; Veronika E. Neubrand, PhD, University of Granada, Granada, Spain; Celia Oreja-Guevara, MD, PhD, Hospital Clinico San Carlos, Madrid, Spain, Jacqueline Palace, DM, Nuffield Department of Clinical Neurology and Oxford University Hospitals Trust, Oxford, UK; Lekha Pandit, MD, PhD, Nitte University, Mangalore, India; Sarah M. Planchon, PhD, Cleveland Clinic, Cleveland, OH, USA; Anne-Katrin Pröbstel, MD, University Hospital of Basel, Basel, Switzerland; Peiqing Qian, MD, Swedish Neuroscience Institute, Seattle, WA, USA; Chao Quan, MD, PhD, Huashan Hospital, Shanghai Medical College, Fudan University, Shanghai, China; Pavle Repovic, MD, PhD, Swedish Neuroscience Institute, Seattle, WA, USA; Claire Riley, MD, Columbia University Irving Medical Center, New York, NY, USA; Marius Ringelstein, MD, Department of Neurology, Heinrich-Heine-University Düsseldorf, Düsseldorf, Germany; Juan I.Rojas, MD, Hospital Universitario CEMIC, Buenos Aires, Argentina; Dalia Rotstein, MD, MPH, University of Toronto, Toronto, Ontario, Canada; Klemens Ruprecht, MD, Department of Neurology, Charité—Universitätsmedizin Berlin, Germany; Maria José Sá, MD, PhD, Centro Hospitalar São João and University Fernando Pessoa, Porto, Portugal; Albert Saiz, MD, PhD, Hospital Clinic and IDIBAPS, Barcelona, Spain; Sara Salama, MD, PhD, University of Alexandria, Egypt; Sasitorn Siritho, MD, Bumrungrad International Hospital, Bangkok, Thailand; Aksel Siva, MD, Istanbul University Cerrahpasa School of Medicine, Istanbul, Turkey; Terry J. Smith, MD, University of Michigan Medical School, Ann Arbor, MI, USA; Elias S. Sotirchos, M.D., Johns Hopkins University, Baltimore, MD, USA; Ibis Soto de Castillo, MD, Hospital Clinico Maracaibo, Zulia, Venezuela; Silvia Tenembaum, MD, National Pediatric Hospital Dr. Juan P. Garrahan, Ciudad de Buenos Aires, Argentina; Pablo Villoslada, MD, Hospital del Mar Barcelona, Barcelona, Spain; Barbara Willekens, MD, PhD, Antwerp University Hospital and University of Antwerp, Antwerp, Belgium; Dean Wingerchuk, MD, Mayo Clinic, Scottsdale, AZ USA; Bassem I. Yamout, MD, Harley Street Medical Center, Abu Dhabi, UAE; Michael Yeaman, PhD, Los Angeles Biomedical Research Institute at Harbor-University of California at Los Angeles (UCLA) Medical Center, Torrance, CA, USA and David Geffen School of Medicine at UCLA, Los Angeles, CA, USA.

### Protocol registration

The protocol for this systematic review and meta-analysis has not been registered.

### Statistical analysis

This was performed by SA under the supervision of SAB and JS (Professor of Biostatistics at Griffith University and Distinguished Professor at Charles Sturt University).
