# Supplementary material for: Sex ratio and age of onset in AQP4 antibody-associated NMOSD: a review and meta-analysis
Source: J Neurol. 2024 Jul 3;271(8):4794–812. doi: 10.1007/s00415-024-12452-8 (PMC11319503; doi:10.1007/s00415-024-12452-8)
Supplement: Supplementary file 1 — (DOCX 142 kb) [file 415_2024_12452_MOESM1_ESM.docx]

**Supplementary Table 1. Summary of included studies, quoted and estimated statistics and Johanna Briggs critical appraisal assessment**

| No. | Seq | Study [Ref] | Year of study | | Region | | Female Life-expectancy (Yrs) | | Study design | | Setting | Recruitment | Diagnostic criteria | AQP4 assay | AQP4 antibody postive (%) | Estimated response rate | N | | Fem | | Mal | Age of onset (Yrs)  Mean ± SD  Median (range) | | Sample frame | | | Sampling | | | Sample size | | | Subjects & setting | | | Coverage | | | Identifi-cation | | Measure | Statistics | Response  rate |
| --- | --- | --- | --- | --- | --- | --- | --- | --- | --- | --- | --- | --- | --- | --- | --- | --- | --- | --- | --- | --- | --- | --- | --- | --- | --- | --- | --- | --- | --- | --- | --- | --- | --- | --- | --- | --- | --- | --- | --- | --- | --- | --- | --- |
| ***Whole Population Studies*** | | | |  | |  | |  | |  | |  |  |  |  |  |  |  | | |  |  | | |  | | |  | | |  | | |  | | |  | | |  |  |  |  |
| 1 | 1 | Aboul-Enein et al., 2013 [28] | 2011 | | Austria | | 83.78 | | Prevalence study | | National testing laboratory, supported by national collaboration of neurologists | Positive AQP4 antibody result in clinically suspected cases of NMO or NMOSD | AQP4 antibody positive | M23 AQP4 immunofluorescence live cell-based assay | 100 | >28% of expected | 71 | | 62 | | 9 | 45.7 ± 14.1^#^  (12 - 79) | | Yes | | | Yes | | | Yes | | | Yes | | | Yes | | | Yes | | Yes | Yes | Yes |
| 2 | 1 | Adoni et al., 2010 [29]* | 2010 | | São Paulo, Brazil | | 79.39 | | Case series | | Single centre | Neurology clinic | Wingerchuk 1999 | Mayo Clinic live cell-based assay | 64.3 | Not estimated | 28 | | 25 | | 3 | 25.4 ± 11.4  26 (7 - 55) | | Yes | | | Yes | | | No | | | Unclear | | | Yes | | | Yes | | Yes | Yes | No |
|  | 2 |  |  | |  | |  | |  | |  |  |  |  | 100 | Not estimated | 18 | | 16 | | 2 | 24.2 ± 12.9  25 (7 -55) | | Yes | | | Yes | | | No | | | Unclear | | | Yes | | | Yes | | Yes | Yes | No |
| 3 | 1 | Altintas et al., 2015 [30] | 2015 | | Turkey | | 80.67 | | Case series | | Academic hospitals | Neurology clinics | Wingerchuk 2006 | Euroimmun transfected fixed cell-based assay | 62.5 | Not estimated | 77 | | 65 | | 12 | 39.0 ± 12.7 | | Yes | | | Yes | | | Yes | | | Unclear | | | Yes | | | Yes | | Yes | Yes | No |
| 4 | 1 | Alves et al., 2022 [31] | 2020 | | Goiás, Brazil | | 79.39 | | Case series | | Single centre | Population-based cross-sectional study from a CNS demyelinating disease centre | IPND 2015 | Not stated | 35.4 | Not estimated | 48 | | 39 | | 9 | 36.7 ± 16.0  (9 - 66) | | Yes | | | Yes | | | Yes | | | Yes | | | Yes | | | Yes | | Unclear | Yes | No |
| 5 | 1 | Asgari et al., 2011 [32] | 2008 | | Southern Denmark | | 83.02 | | Prevalence study | | Neurology and ophthalmology clinics | Clinics and national registry diagnostic codes for hospital visits | Wingerchuk 2006 | AQP4 transfected HEK293 live cell-based assay | 61.9 | Not estimated | 42 | | 31 | | 11 | 35.6 ± 11.2^#^  (15 - 64) | | Yes | | | Yes | | | Yes | | | Yes | | | Yes | | | Yes | | Yes | Yes | No |
| 6 | 1 | Barhate et al., 2014 [33] | 2013 | | Mumbai, India | | 72.17 | | Case series | | Single centre | Hospital (in-patient and out-patient) | Wingerchuk 2006 & Wingerchuk 2007 | M1 or M23 AQP4 transfected HEK293 live cell-based assay | 84.1 | Not estimated | 44 | | 39 | | 5 | 31.5 ± 14.8^†^  (8 - 72) | | Yes | | | Yes | | | Yes | | | Yes | | | Yes | | | Yes | | Yes | Yes | No |
|  | 2 |  |  | |  | |  | |  | |  |  |  |  | 100 |  | 37 | | 33 | | 4 | 33.2 ± 15.3^†^  (8 - 72) | | Yes | | | Yes | | | Yes | | | Yes | | | Yes | | | Yes | | Yes | Yes | No |
| 7 | 1 | Bennis et al., 2019 [34] | 2015 | | Casablanca, Morocco | | 74.31 | | Case series | | Single centre | Hospital (in-patient and out-patient) | IPND 2015 | Biomnis transfected fixed cell-based assay | 25.0 | Not estimated | 64 | | 50 | | 14 | 35.7 ± 10.7  36 (16 - 63) | | Yes | | | Yes | | | Yes | | | Yes | | | Yes | | | Yes | | Yes | Yes | No |
| 8 | 1 | Bichuetti et al., 2009 [35] | 2007 | | São Paulo, Brazil | | 79.39 | | Case series | | Single centre | Neuroimmunology clinic | Wingerchuk 2006 | Commercial assay (not further specified) | 17.1 | Not estimated | 41 | | 29 | | 12 | 32.6 ± 11.5  (20 - 60) | | Yes | | | Yes | | | Yes | | | Yes | | | Yes | | | Yes | | Unclear | Yes | No |
| 9 | 1 | Bizzoco et al., 2009 [36]* | 2006 | | Firenze & Prato, Italy | | 84.9 | | Prevalence study | | Adult and paediatric centres | Neurology clinics | Wingerchuk 2007 | Tissue (rat) immunofluorescence | 76.9 | Not estimated | 13 | | 12 | | 1 | 37.3 ± -16.0^†^  40 (9 - 69) | | Yes | | | Yes | | | No | | | Yes | | | Yes | | | Yes | | Yes | Yes | No |
|  | 2 |  |  | |  | |  | |  | |  |  |  |  | 100 |  | 7 | | 7 | | 0 | 41.0 ± 15.5  40 (25 - 69) | | Yes | | | Yes | | | No | | | Yes | | | Yes | | | Yes | | Yes | Yes | No |
| 10 | 1 | Bukhari et al., 2017 [37]* | 2013 | | Australia & New Zealand | | 84.84 | | Prevalence study | | National networks of neuroimmunology clinics | Suspected NMOSD from 36 adult and paediatric clinics | IPND 2015 | Oxford live cell-based assay, Euroimmun AQP transfected fixed cell-based assay or tissue (mouse) indirect immunofluoresence | 90.1 | 76% (capture-recapture method) | 147 | | 126 | | 21 | 41.7 ± 17.4  40 (13 - 85) | | Yes | | | Yes | | | Yes | | | Yes | | | Yes | | | Yes | | Yes | Yes | Yes |
|  | 2 | Bukhari et al., 2020 [113] |  | |  | |  | |  | |  |  |  |  | 100 |  | 68 | | 61 | | 7 | 44.8 ± 15.3^‡^  40.5 (13 - 85) | | Yes | | | Yes | | | Yes | | | Yes | | | Yes | | | Yes | | Yes | Yes | Yes |
| 11 | 1 | Cabre et al., 2001 [38] | 1999 | | Martinique, Caribbean | | 77.96 | | Prevalence study | | Single centre | Review of local case files (including private neurologists) | Wingerchuk 1999 | N/A | 0 | Not estimated | 17 | | 17 | | 0 | 31.7 ± 9.2 | | Yes | | | Yes | | | No | | | Yes | | | Yes | | | Yes | | No | Yes | No |
| 12 | 1 | Cabrera-Gomez et al., 2009 [39]* | 2004 | | Cuba | | 80.25 | | Prevalence study | | Hospitals and clinics | Nationwide survey involving neurologists, ophthalmologists and general physicians | Wingerchuk 1999 | N/A | 0 | Not estimated | 58 | | 51 | | 7 | 31.8 ± 11.1  (11 - 62) | | Yes | | | Yes | | | Yes | | | Yes | | | Yes | | | Yes | | No | Yes | No |
| 13 | 1 | Ceccarelli et al., 2020 [40] | 2017 | | United Arab Emirates | | 78.42 | | Case series | | Single centre | Chart review of neurological institute | IPND 2015 | Not stated or referenced (testing performed by BN Labcorp, Burlington - possibly ELISA) | 100 | Not estimated | 5 | | 5 | | 0 | 40.8 ± 12.5^†^  46 (19 - 50) | | Yes | | | Yes | | | No | | | Yes | | | Yes | | | Yes | | Unclear | Yes | No |
| 14 | 1 | Chan et al., 2013 [41] | 2010 | | Hong Kong, China | | 80.49 | | Case series | | Single centre | Consecutive CNS IDD cases | Wingerchuk 2006 & AQP4 antibody positive | AQP4 transfected HEK293 live cell-based assay | 88.9 | Not estimated | 47 | | 41 | | 6 | 45.1 ± 15.4  (16 - 76) | | Yes | | | Yes | | | Yes | | | Yes | | | Yes | | | Yes | | Yes | Yes | No |
| 15 | 1 | Collongues et al., 2010 [42] | 2008 | | France | | 85.09 | | Case series | | 25 centres across France | Suspected NMO from MS centres using a questionnaire | Wingerchuk 2006 | Tissue (rat) immunofluorescence | 48.0 | Not estimated | 125 | | 94 | | 31 | 34.5 ± 13.2  34.7 (4 - 66) | | Yes | | | Yes | | | Yes | | | Yes | | | Yes | | | Yes | | Yes | Yes | No |
| 16 | 1 | Cossburn et al., 2012 [43]* | 2006 | | South East Wales, UK | | 82.99 | | Prevalence study | | Single centre with outreach satellite clinics | Regional neuro-inflammatory database, neurologist referral, hospital databases and laboratory data | Wingerchuk 2007 | Not stated or referenced | 76.9 | Not estimated | 14 | | 12 | | 2 | 36.8 ± 22.2  40 (4 - 70) | | Yes | | | Yes | | | No | | | Yes | | | Yes | | | Yes | | No | Yes | No |
|  | 2 |  |  | |  | |  | |  | |  |  |  |  | 100 |  | 10 | | 8 | | 2 | 34.9 ± 22.6  42.5 (4 - 62) | | Yes | | | Yes | | | No | | | Yes | | | Yes | | | Yes | | No | Yes | No |
| 17 | 1 | Daoudi & Bouzar, 2016 [44] | 2014 | | Tizi-Ouzou, Algeria | | 78.12 | | Case series | | Single centre | Chart review of MS clinic attendees | IPND 2015 | Not clearly defined - state cell-based assay preferred | 37.5 | Not estimated | 8 | | 6 | | 2 | 29.4 ± 9.8^#^  (16 - 44) | | Yes | | | Yes | | | No | | | Yes | | | Yes | | | Yes | | No | Yes | No |
| 18 | 1 | Del Negro et al., 2017 [45] | 2012 | | Brasilia, Brazil | | 79.39 | | Case series | | Single centre | Subjects for whom AQP4 antibody testing requested | Wingerchuk 2006 | M1 AQP4 ELISA-R kit | 73.5 | Not estimated | 34 | | 30 | | 4 | 34.6 ± 17.2  (4 - 68) | | Yes | | | Yes | | | Yes | | | Yes | | | Yes | | | Yes | | Yes | Yes | No |
|  | 2 |  |  | |  | |  | |  | |  |  |  |  | 100 |  | 25 | | 23 | | 2 | 33.8 ± 17.3 | | Yes | | | Yes | | | Yes | | | Yes | | | Yes | | | Yes | | Yes | Yes | No |
| 19 | 1 | Drulovic et al., 2019 [46] | 2017 | | Belgrade, Serbia | | 78.28 | | Case series | | Single centre | Retrospective casenote review | IPND 2015 | Euroimmun AQP4 transfected fixed cell-based assay | 89.2 | Not estimated | 74 | | 63 | | 11 | 38.8 ± 12.8^‡^  40 (7 – 68) | | Yes | | | Yes | | | Yes | | | Yes | | | Yes | | | Yes | | Yes | Yes | No |
| 20 | 1 | Eskandarieh et al., 2017 [47]* | 2016 | | Tehran, Iran | | 79.09 | | Prevalence study | | Single centre | Hospital diagnosed cases and register of cases | IPND 2015 | ELISA | 46.8 | Not estimated | 103 | | 86 | | 17 | 31.5 ± 12.0^#^  (8 - 68) | | Yes | | | Yes | | | Yes | | | Yes | | | Yes | | | Yes | | Yes | Yes | No |
| 21 | 1 | Espiritu et al., 2019 [48] | 2019 | | Manila, Phillippines | | 73.6 | | Prevalence study | | Single centre | Consecutive cases at tertiary hospital | Not stated | AQP4 transfected EU90 cells (Eurofins Biomnis) | 44.4 | Not estimated | 18 | | 13 | | 5 | 27.8 ± 9.1  26 (12 - 47) | | Yes | | | Yes | | | No | | | Unclear | | | Unclear | | | Unclear | | Yes | Yes | No |
|  | 2 |  |  | |  | |  | |  | |  |  |  |  | 100 |  | 8 | | 7 | | 1 | 26.9 ± 9.6  24 (17 - 47) | | Yes | | | Yes | | | No | | | Unclear | | | Unclear | | | Unclear | | Yes | Yes | No |
| 22 | 1 | Etemadifar et al., 2020 [49] | 2019 | | Isfahan, Iran | | 79.09 | | Case series for MRI analysis | | Single centre | Retrospective casenote review | IPND 2015 | Euroimmun AQP4 transfected fixed cell-based assay | 48.1 | Not estimated | 108 | | 89 | | 19 | Not stated | | Yes | | | Yes | | | Yes | | | Yes | | | Yes | | | Yes | | Yes | Yes | No |
| 23 | 1 | Flanagan et al., 2016a [50] | 2012 | | Olmstead County, Minnesota, US | | 80.73 | | Prevalence study | | Population-based survey | Retrospective casenote review of subjects with CNS demyelinating disease | Wingerchuk 2006 | Mayo Clinic live M1 AQP4 transfected cell-based assay | 83.3 | Not estimated | 6 | | 5 | | 1 | 34.8 ± 17.8^‡^  37 (10 - 55) | | Yes | | | Yes | | | No | | | Yes | | | Yes | | | Yes | | Yes | Yes | No |
| 24 | 1 | Flanagan et al., 2016b [50] | 2012 | | Martinque, Carribean | | 77.96 | | Prevalence study | | Population-based survey | Retrospective casenote review of subjects with CNS demyelinating disease | Wingerchuk 2006 | Mayo Clinic live M1 AQP4 transfected live cell-based assay | 79.5 | Not estimated | 39 | | 35 | | 4 | 41.5 ± 15.8^‡^  35 (14 - 82) | | Yes | | | Yes | | | Yes | | | Yes | | | Yes | | | Yes | | Yes | Yes | No |
| 25 | 1 | Fukuda et al., 2022 [51] | 2019 | | Bahai, Brazil | | 79.39 | | Case series | | Single centre | Consecutive clinic attendees at tertiary clinic | IPND 2015 | Not stated | 60.4 | Not estimated | 91 | | 72 | | 19 | 36.3 ± 13.6 | | Yes | | | Yes | | | Yes | | | Yes | | | Yes | | | Yes | | Unclear | Yes | No |
| 26 | 1 | Gao et al., 2019 [52] | 2018 | | Shanghai, China | | 80.49 | | Case series | | Single centre | Retrospective review of demyelinating disease registry | IPND 2015 | Euroimmun transfected fixed cell-based assay | 70.2 | Not estimated | 84 | | 74 | | 10 | Not stated | | Yes | | | Yes | | | Yes | | | No | | | Yes | | | Yes | | Yes | Yes | No |
| 27 | 1 | Gracia et al., 2022 [53] | 2020 | | Central America and Caribbean | | 76.19 | | Case series | | Multicentre, multinational | Retrospective study of cases across centres | IPND 2015 and Wingerchuk 2006 | Cell-based assay (not further specified) | 52.7 | Not estimated | 186 | | 158 | | 28 | 37.5 ± 3.7^‡^  37 (28 - 48) | | Yes | | | Yes | | | Yes | | | Yes | | | Yes | | | Yes | | Unclear | Yes | No |
| 28 | 1 | Holroyd et al., 2018 [54] | 2016 | | Abu Dhabi, United Arab Emirates | | 78.42 | | Prevalence study | | Four hospital sites | Retrospective chart review of patients with CNS demyelinating disease identified by ICD codes | IPND 2015 | Not stated | 80.0 | Not estimated | 10 | | 7 | | 3 | 43.0 ± 18.7  43 (8 - 74) | | Yes | | | Yes | | | No | | | Yes | | | Yes | | | Yes | | Unclear | Yes | No |
| 29 | 1 | Hor et al., 2018 [55] | 2017 | | Penang, Malaysia | | 77.08 | | Prevalence study | | Single centre | Retrospective chart review of hospital clinic attendees and referrals from neurologists in private practice | IPND 2015 | Euroimmun transfected fixed cell-based assay | 100 | Not estimated | 14 | | 14 | | 0 | 43.1 ± 22.3^#^  (18 - 94) | | Yes | | | Yes | | | No | | | Yes | | | Yes | | | Yes | | Yes | Yes | No |
| 30 | 1 | Ibis et al., 2021 [56] | 2018 | | Venezuela | | 78.17 | | Prevalence study | | Multicentre | National database | IPND 2015 | Mayo Clinic live cell-based assay | 31.3 | Not estimated | 249 | | 206 | | 43 | 34.0 ± 14.8  39 (5 - 71) | | Yes | | | Yes | | | Yes | | | Yes | | | Yes | | | Yes | | Yes | Yes | No |
| 31 | 1 | Jagtap et al., 2015 [57] | 2012 | | Kerala, India | | 72.17 | | Case series | | Single centre | Local database | Miller 2008 | Not stated | 30.8 | Not estimated | 26 | | 21 | | 5 | 27.0 ± 12.4^#^  (9 - 58) | | Yes | | | Yes | | | No | | | Yes | | | Yes | | | Yes | | Unclear | Yes | No |
| 32 | 1 | Jiao et al., 2013 [58] | 2011 | | US | | 80.73 | | Case series | | Central referral laboratory | Cases referred for AQP4 antibody testing | Wingerchuk 2006 plus AQP4 antibody positivity | Mayo Clinic tissue (mouse) immunofluorescence, ELISA, Euroimmun fixed cell-based and FACS live cell-based assays | 88.1 | Not estimated | 159 | | 136 | | 23 | 38.6 ± 13.2^‡^  (5 -71) | | Yes | | | Yes | | | Yes | | | Yes | | | Yes | | | Yes | | Yes | Yes | No |
|  | 2 |  |  | |  | |  | |  | |  |  |  |  | 100 |  | 140 | | 126 | | 14 | 38.5 ± 12.6^‡^  39 (5 -71) | | Yes | | | Yes | | | Yes | | | Yes | | | Yes | | | Yes | | Yes | Yes | No |
| 33 | 1 | Jonsson et al., 2019 [59] | 2013 | | Sweden | | 83.97 | | Prevalence study | | Population-based survey | National registry of hospitalisations | Wingerchuk 2006 | Immunoblot or cell-based assay | Not stated | Not estimated | 92 | | 68 | | 24 | Not stated | | Yes | | | Yes | | | Yes | | | Yes | | | Yes | | | Yes | | Yes | Yes | No |
| 34 | 1 | Joshi et al., 2022 [60] | 2016 | | Gujarat, India | | 72.17 | | Case series | | Single centre | CNS inflammatory diseases clinic survey | Wingerchuk 2006 | AQP4 transfected cell-based FACS assay | 56.6 | Not estimated | 46 | | 34 | | 12 | 28.8 ± 8.3^#^  (13 - 50) | | Yes | | | Yes | | | Yes | | | Yes | | | Yes | | | Yes | | Yes | Yes | No |
|  | 2 |  |  | |  | |  | |  | |  |  |  |  | 100 |  | 26 | | 23 | | 3 | 36.8 ± 12.1^#^  (17 - 65) | | Yes | | | Yes | | | Yes | | | Yes | | | Yes | | | Yes | | Yes | Yes | No |
| 35 | 1 | Kashipazha et al., 2015 [61] | 2015 | | Khuzestan, Iran | | 79.09 | | Prevalence study | | Population-based survey | MS registry, neurology clinic referrals and private neurologist clinics with ON, TM or NMO | Wingerchuk 2006 | Not stated | 38.0 | Not estimated | 50 | | 44 | | 6 | 29.2 ± 11.1^#^ | | Yes | | | Yes | | | Yes | | | Unclear | | | Yes | | | Yes | | Unclear | Yes | No |
| 36 | 1 | Kim et al., 2012 [62] | 2010 | | Goyang, Korea | | 86.09 | | Case series | | Single centre | Consecutive attendees at national referral centre | AQP4 antibody positive | ELISA and Euroimmun fixed cell-based assay | 100 | Not estimated | 106 | | 97 | | 9 | 32.5 ± 10.3^‡^  32 (7 - 59) | | Yes | | | Yes | | | Yes | | | Yes | | | Yes | | | Yes | | Yes | Yes | No |
| 37 | 1 | Kishk et al., 2021 [63] | 2018 | | Cairo, Egypt | | 74.14 | | Case series | | Single centre | Retrospective casenote review of cases with NMO/NMOSD | IPND 2015 | ELISA or cell-based assay (specific assay not stated) | 50.0 | Not estimated | 70 | | 58 | | 12 | 28.9 ± 10.5 | | Yes | | | Yes | | | Yes | | | Yes | | | Yes | | | Yes | | Unclear | Yes | No |
|  | 2 |  |  | |  | |  | |  | |  |  |  |  | 100 |  | 35 | | 29 | | 6 | 30.1 ± 9.3 | | Yes | | | Yes | | | Yes | | | Yes | | | Yes | | | Yes | | Unclear | Yes | No |
| 38 | 1 | Kitley et al., 2014 [64] | 2013 | | Oxford, UK | | 82.99 | | Case series | | Single centre | Prospective clinical NMO database | AQP4 antibody positive | Oxford, M23 AQP4 transfected HEK live cell-based assay | 100 | Not estimated | 20 | | 18 | | 2 | 44.9 ± 14.8 | | Yes | | | Yes | | | No | | | Yes | | | Yes | | | Yes | | Yes | Yes | No |
| 39 | 1 | Kleiter et al., 2016 [65] | 2013 | | Germany | | 84.77 | | Case series | | National survey | NEMOS registry | Wingerchuk 2006 or AQP4 antibody positive | Not stated | 84.3 | Not estimated | 185 | | 152 | | 33 | 40.3 ± 14.9 | | Yes | | | Yes | | | Yes | | | Yes | | | Yes | | | Yes | | Unclear | Yes | No |
|  | 2 | Jarius et al., 2012 [114] | 2011 | |  | |  | |  | |  |  |  |  | 100 |  | 137 | | 125 | | 12 | 42.8 ± 13.6^‡^  40 (10 - 81) | | Yes | | | Yes | | | Yes | | | Yes | | | Yes | | | Yes | | Unclear | Yes | No |
| 40 | 1 | Mealy et al., 2018 [66] | 2018 | | Baltimore and New York, US | | 80.73 | | Case series for mortality study | | Two centres | Retrospective, clinic-based survey | IPND 2015 | Not stated | 79.6 | Not estimated | 427 | | 367 | | 60 | 39.9 ± 16.7 | | Yes | | | Yes | | | Yes | | | Unclear | | | Yes | | | Yes | | Unclear | Yes | No |
| 41 | 1 | Mireles-Ramirez et al., 2022 [67] | 2019 | | Western Mexico | | 78.86 | | Prevalence study | | Single regional referral centre | Consecutive cases at tertiary hospital | IPND 2015 | Not stated | 55.2 | Not estimated | 67 | | 50 | | 17 | 36.0 ±  ( - 65) | | Yes | | | Yes | | | Yes | | | Yes | | | Yes | | | Yes | | Unclear | Yes | No |
| 42 | 1 | Mirmosayyeb et al., 2021 [68] | 2021 | | Isfahan, Iran | | 79.09 | | Case series | | Single centre | CND demyelinating disease clinic survey | IPND 2015 | Not stated | Not stated | Not estimated | 220 | | 167 | | 53 | Not stated | | Yes | | | Yes | | | Yes | | | No | | | Yes | | | Yes | | Unclear | Yes | No |
| 43 | 1 | Miyamoto et al., 2018 [69] | 2011 | | Japan | | 86.94 | | Prevalence study | | Nationwide survey | Clinic-based random sampling | Wingerchuk 2006 | Not stated | Not stated | Not estimated | 1042 | | 901 | | 141 | 42.2 ± | | Yes | | | Yes | | | Yes | | | Yes | | | Yes | | | Yes | | Unclear | Yes | No |
|  | 2 | Nagaishi et al., 2011 [115] | 2011 | |  | |  | |  | |  |  |  |  | 100 |  | 549 | | 502 | | 47 | 44.3 ± 13.5^‡^  44 (3 - 86) | | Yes | | | Yes | | | Yes | | | Yes | | | Yes | | | Yes | | Unclear | Yes | No |
| 44 | 1 | Netravathi et al., 2019 [70] | 2018 | | Bangalore, India | | 72.17 | | Case series | | Single centre | Retrospective study at tertiary hospital | IPND 2015 + AQP4 antibody positive | Not stated | 100 | Not estimated | 101 | | 90 | | 11 | 31.0 ± 12.5  (9 - 65) | | Yes | | | Yes | | | Yes | | | Yes | | | Yes | | | Yes | | Unclear | Yes | No |
| 45 | 1 | Pandit & Kundapur., 2014 [71] | 2013 | | Mangalore, India | | 72.17 | | Prevalence study | | Clinic-based survey | Prospectively maintained CNS demyelinating disease registry | Wingerchuk 2006/2007 | Not stated | Not stated | Not estimated | 11 | | 6 | | 5 | 40.3 ± 12.5^‡^ | | Yes | | | Yes | | | No | | | Yes | | | Yes | | | Yes | | Unclear | Yes | No |
| 46 | 1 | Papais-Alvarenga et al., 2015 [72]* | 2011 | | South America | | 79.39 | | Case series | | Multicentre | Cross-sectional survey across MS centres | Wingerchuk 2006/2007 | Not stated | 42.0 | Not estimated | 226 | | 191 | | 35 | 31.2 ± 13.5  (2 - 68) | | Yes | | | Yes | | | Yes | | | Yes | | | Yes | | | Yes | | Unclear | Yes | No |
| 47 | 1 | Papp et al., 2020 [73] | 2016 | | Budapest, Hungary | | 79.59 | | Prevalence study | | Multicentre population-based survey | Positive AQP4 antibody result from participating laboratories. | IPND 2015 | Euroimmun transfected fixed cell-based assay | 83.1 | Not estimated | 154 | | 135 | | 19 | 42.0 ± 14.5^‡^  39.5 (6 - 83) | | Yes | | | Yes | | | Yes | | | Yes | | | Yes | | | Yes | | Yes | Yes | No |
|  | 2 | Papp et al., 2021a [74]^§^ | 2015 | | Budapest, Hungary | |  | | Prevalence study | | Multicentre population-based survey | Local registry and laboratory results | IPND 2015 plus AQP4 antibody positive | Euroimmun transfected fixed cell-based assay or Oxford live cell-based assay | 100 | Not estimated | 99 | | 90 | | 9 | 42.8 ± 15.4^‡^  41 (6 - 83) | | Yes | | | Yes | | | Yes | | | Yes | | | Yes | | | Yes | | Yes | Yes | No |
| 48 | 1 | Papp et al., 2021b [74]^§^ | 2015 | | Denmark | | 83.02 | | Prevalence study | | Nationwide survey | National registry | IPND 2015 plus AQP4 antibody positive | Euroimmun transfected fixed cell-based assay or Oxford live cell-based assay | 100 | Not estimated | 35 | | 31 | | 4 | 45.5 ± 15.2^‡^  47 (12 - 76) | | Yes | | | Yes | | | Yes | | | Yes | | | Yes | | | Yes | | Yes | Yes | No |
| 49 | 1 | Paz et al., 2021 [75] | 2017 | | Fortaleza, Brazil | | 79.39 | | Case series | | Single centre | Cross-sectional survey in a neurology clinic | IPND 2015 | Not stated | 60.5 | Not estimated | 38 | | 33 | | 5 | 38.0 ± 12.0 | | Yes | | | Yes | | | Yes | | | Yes | | | Yes | | | Yes | | Unclear | Yes | No |
| 50 | 1 | Rafiee et al., 2020 [76] | 2019 | | Tehran, Iran | | 79.09 | | Case-control study | | Single centre | Neurology clinic | IPND 2015 | Not stated | Not stated | Not estimated | 153 | | 123 | | 30 | 30.2 ± 10.7 | | Yes | | | Yes | | | Yes | | | Yes | | | Yes | | | Yes | | Unclear | Yes | No |
| 51 | 1 | Rivera et al., 2008 [77] | 2005 | | Mexico City, Mexico | | 78.86 | | Case series | | Single centre | Retrospective casenote review | Wingerchuk 1999 | Not tested | Not tested | Not estimated | 34 | | 24 | | 10 | 34.0 ± | | Yes | | | Yes | | | No | | | Yes | | | Yes | | | Yes | | No | Yes | No |
| 52 | 1 | Rojas et al., 2020 [78] | 2020 | | Argentina | | 79.5 | | Case series | | Multicentre | Prospective database maintained by MS specialists | IPND 2015 | Tissue indirect immunofluorescence or ELISA | 54.7 | Not estimated | 75 | | 59 | | 16 | Not stated | | Yes | | | Yes | | | Yes | | | No | | | Yes | | | Yes | | Yes | Yes | No |
| 53 | 1 | Salama et al., 2018 [79] | 2018 | | Alexandria, Egypt | | 74.14 | | Case series | | Single centre | Casenote review of neuroimmunology clinic | IPND 2015 | ELISA | 50.0 | Not estimated | 20 | | 17 | | 3 | 27.8 ± 13.7  25.5 ( - ) | | Yes | | | Yes | | | No | | | Unclear | | | Yes | | | Yes | | Yes | Yes | No |
| 54 | 1 | Santos et al., 2021 [3] | 2019 | | Portugal | | 84.4 | | Case series | | Multicentre | Nationwide network of MS Centres | IPND 2021 | Euroimmun transfected fixed cell-based assay, Oxford live cell-based assay and Mayo FACS assay | 100.0 | Not estimated | 77 | | 68 | | 9 | 48.4 ± 18.4  (10-87) | | Yes | | | Yes | | | Yes | | | Yes | | | Yes | | | Yes | | Yes | Yes | No |
| 55 | 1 | Sepulveda et al., 2016 [80] | 2015 | | Spain | | 85.68 | | Case series | | Multicentre | National network of MS study centres | Wingerchuk 2006 or AQP4 antibody positive | Tissue indirect immunofluorescence and AQP4 transfected HEK293 live cell-based assay | 81.8 | Not estimated | 181 | | 157 | | 24 | 41.3 ± 12.4^‡^  39 (10 - 77) | | Yes | | | Yes | | | Yes | | | Yes | | | Yes | | | Yes | | Yes | Yes | No |
|  | 2 |  |  | |  | |  | |  | |  |  |  |  | 100 |  | 148 | | 138 | | 10 | 41.2 ± 13.6^‡^  (10-77) | | Yes | | | Yes | | | Yes | | | Yes | | | Yes | | | Yes | | Yes | Yes | No |
| 56 | 1 | Shaygannejad et al., 2018 [81] | 2015 | | Isfahan, Iran | | 79.09 | | Case series | | Single centre | Newly diagnosed NMOSD from an MS clinic | Not stated | Euroimmun transfected fixed cell-based assay | 31.0 | Not estimated | 29 | | 22 | | 7 | Not stated | | Yes | | | Yes | | | No | | | No | | | Yes | | | No | | Yes | Yes | No |
| 57 | 1 | Shosha et al., 2020 [82]* | 2020 | | Arabian Gulf | | 76.15 | | Case series | | 15 neurology/MS centres across 5 countries | NMOSD registry | IPND 2015 | Cell-based assay, indirect immunofluorescence or ELISA | 65.3 | Not estimated | 144 | | 119 | | 25 | 31.0 ± 12.0 | | Yes | | | Yes | | | Yes | | | Unclear | | | Yes | | | Yes | | Yes | Yes | No |
|  | 2 |  |  | |  | |  | |  | |  |  |  |  | 100 |  | 94 | | 85 | | 9 | 32.0 ± 12.0 | | Yes | | | Yes | | | Yes | | | Unclear | | | Yes | | | Yes | | Yes | Yes | No |
| 58 | 1 | Singh et al., 2021 [83] | 2018 | | New Delhi, India | | 72.17 | | Case series | | Single centre | Retrospective casenote review | IPND 2015 | Not stated | 64.2 | Not estimated | 106 | | 77 | | 29 | 32.8 ± 11.4 | | Yes | | | Yes | | | Yes | | | Yes | | | Yes | | | Yes | | Unclear | Yes | No |
| 59 | 1 | Stratos et al., 2020 [84] | 2020 | | Toronto, Canada | | 84.05 | | Case series | | Two centres | Retrospective review of casenotes with suspected NMOSD | IPND 2015 | Not stated | 70.4 | Not estimated | 81 | | 71 | | 10 | 38.9 ± 16.8 | | Yes | | | Yes | | | Yes | | | Unclear | | | Yes | | | Yes | | Unclear | Yes | No |
| 60 | 1 | Sun et al., 2017 [85] | 2015 | | Beijing, China | | 80.49 | | Case series | | Single centre | Consecutive cases at tertiary hospital | IPND 2015 | Indirect immunofluorescence cell-based assay (not further specified or referenced) | 54.6 | Not estimated | 97 | | 82 | | 15 | 37.0 ± 14.5^‡^  (14 - 66) | | Yes | | | Yes | | | Yes | | | Yes | | | Yes | | | Yes | | Unclear | Yes | No |
| 61 | 1 | Tarhan et al., 2022 [86] | 2020 | | Gainesville, FL, US | | 80.73 | | Case series | | Single centre | Retrospective, clinic-based survey | IPND 2015 plus AQP4 antibody positive | ELISA or Mayo Clinic cell-based assay (8/43) | 100 | 11/54 (20%) excluded due to inadequate documentation | 43 | | 36 | | 7 | 40.5 ± 17.4^‡^  37 (6 - 82) | | Yes | | | Yes | | | Yes | | | Yes | | | Yes | | | Yes | | Yes | Yes | Yes |
| 62 | 1 | Tian et al., 2020 [87]* | 2018 | | China | | 80.49 | | National survey | | National database | National administrative healthcare database | ICD-10 G36.0 (IPND 2015) | Cell-based assay (not further specified) or ELISA | Not stated | 98.5% of specialist neurology referral centres | 11,973 | | 9,825 | | 2148 | Not stated | | Yes | | | Yes | | | Yes | | | No | | | Yes | | | Yes | | Unclear | Yes | No |
| 63 | 1 | van Pelt et al., 2016 [88] | 2015 | | Amsterdam, Netherlands | | 83.15 | | Case series | | Single centre | Retrospective survey | AQP4 antibody positive | AQP4 transfected HEK293 live cell-based assay | 100 | Not estimated | 41 | | 35 | | 6 | 42.0 ± 16.1 | | Yes | | | Yes | | | Yes | | | Yes | | | Yes | | | Yes | | Yes | Yes | No |
| 64 | 1 | Wu et al., 2022 [89] | 2017 | | China | | 80.49 | | Incidence study | | National survey | Nationwide medical insurance claim database | IPND 2015 | Not stated | Not stated | Not estimated | 1313 | | 1031 | | 282 | 47.6 ± 13.7  (19 - 89) | | Yes | | | Yes | | | Yes | | | Yes | | | Yes | | | Yes | | Unclear | Yes | No |
| 65 | 1 | Yin et al., 2015 [90] | 2015 | | Guangzhou, China | | 80.49 | | Case series | | Single centre | Retrospective analysis of consecutive attendees to a neurology clinic | Wingerchuk 2006 | Euroimmun AQP4 transfected fixed cell-based assay | 87.0 | Not estimated | 108 | | 92 | | 16 | 36.2 ± 12.6  37 (15 - 70) | | Yes | | | Yes | | | Yes | | | Unclear | | | Yes | | | Yes | | Yes | Yes | No |
|  | 2 |  |  | |  | |  | |  | |  |  |  |  | 100 |  | 94 | | 85 | | 9 | 35.5 ± 12.7 | | Yes | | | Yes | | | Yes | | | Unclear | | | Yes | | | Yes | | Yes | Yes | No |
| ***Paediatric Studies*** | | |  | |  | |  | |  | |  |  |  |  |  |  |  | | |  |  |  |  | | |  | | |  | | |  | | |  | | |  | | |  |  |  |
| 66 | 1 | Banwell et al., 2008 [91] | 2008 | | Toronto, Canada and Buenos Aires, Argentina | |  | | Case series | | Two paediatric CNS inflammatory disease clinics | Retrospective survey (onset <18 years) | Wingerchuk 1999 | Tissue immunofluorescence | 47.1 | Not estimated | 17 | | 14 | | 3 | 10.0 ± 3.1^‡^  10.4 (4 - 15) | | Yes | | | Unclear | | | No | | | Yes | | | Yes | | | Yes | | Yes | Yes | No |
| 67 | 1 | Camera et al., 2022 [92] | 2022 | | UK | |  | | Case series | | Six tertiary referral centres for NMO | Retrospective survey (onset <18 years) | IPND 2015 plus AQP4 antibody positive | Oxford live cell-based assay | 100 | Not estimated | 49 | | 43 | | 6 | 12 ± 4.1 | | Yes | | | Yes | | | Yes | | | Yes | | | Yes | | | Yes | | Yes | Yes | No |
| 68 | 1 | Chitnis et al., 2016 [11] | 2013 | | US | |  | | Case series | | US Network of Paediatric MS Centres | Retrospective survey (children and adolescents) | Wingerchuk 2006 | Not stated | 63.2 | Not estimated | 38 | | 26 | | 12 | 10.2 ± 4.7 | | Yes | | | Yes | | | Yes | | | Yes | | | Yes | | | Yes | | Unclear | Yes | No |
| 69 | 1 | Collongues et al., 2010 [93] | 2008 | | France | |  | | Case series | | Network of 25 French MS centres | Retrospective survey (onset <18 years) | Wingerchuk 2006 | NMO IgG (not otherwise stated) | 66.7 | Not estimated | 12 | | 9 | | 3 | 12.9 ± 4.4  14 (4 - 17) | | Yes | | | Yes | | | No | | | Yes | | | Yes | | | Yes | | Unclear | Yes | No |
| 70 | 1 | Huppke et al., 2010 [94] | 2009 | | Göttingen, Germany | |  | | Case series | | National referral centre | Retrospective survey (age <18 years) | Wingerchuk 2006 | Radioimmunoprecipitation assay | 28.6 | Not estimated | 7 | | 5 | | 2 | 10.3 ± 3.7^†^  11 (5 - 14) | | Yes | | | Yes | | | No | | | Yes | | | Yes | | | Yes | | Yes | Yes | No |
| 71 | 1 | Lin et al., 2020 [95] | 2015 | | Taiwan | |  | | Case series | | National Health Insurance database | Retrospective survey (onset <20 years) | ICD-9-CM 341.0 code | Not stated | Not stated | Not estimated | 42 | | 30 | | 12 | Not stated | | Yes | | | Yes | | | Yes | | | Yes | | | Yes | | | Yes | | Unclear | Yes | No |
| 72 | 1 | Lotze et al., 2008 [96] | 2007 | | Houston, TX, US | |  | | Case series | | Single centre | Retrospective casenote review (age <18 years) | Krupp 2007 | Not stated | 66.7 | Not estimated | 9 | | 9 | | 0 | 12.1 ± 4.7  14 (2 - 16) | | Yes | | | Yes | | | No | | | Yes | | | Yes | | | Yes | | Unclear | Yes | No |
| 73 | 1 | Martins et al., 2022 [97] | 2019 | | Portugal | |  | | Multicentre | | Nationwide survey | Retrospective survey (onset <18 years) | IPND 2015 | Euroimmun AQP4 transfected fixed cell-based assay, Oxford live cell-based assay or Mayo Clinic live cell FACS assay | 100 | Not estimated | 6 | | 5 | | 1 | 12.2 ± 4.6  13.5 (4 - 17) | | Yes | | | Yes | | | No | | | Yes | | | Yes | | | Yes | | Yes | Yes | No |
| 74 | 1 | McKeon et al., 2008 [98] | 2007 | | Rochester, MN, US | |  | | Case series | | Single centre | Retrospective casenote review following laboratory result survey (age <19 years) | Wingerchuk 2006 | Tissue immunofluorescence or Mayo Clinic AQP4 transfectied HEK293 live cell-based assay | 100 | Not estimated | 58 | | 51 | | 7 | 11.5 ± 3.0^‡^  12 (4 - 18) | | Yes | | | Yes | | | Yes | | | Yes | | | Yes | | | Yes | | Yes | Yes | No |
| 75 | 1 | Paolilo et al., 2020 [99] | 2020 | | Brazil and Europe | |  | | Case series | | Multicentre, multinational study | Retrospective survey (onset <18 years) | IPND 2015 plus AQP4 antibody positive | Live cell-based assay | 100 | Not estimated | 67 | | 54 | | 13 | 10.2 ± 3.6 | | Yes | | | Unclear | | | Yes | | | Yes | | | Yes | | | Yes | | Yes | Yes | No |
| 76 | 1 | Yamaguchi et al., 2016 [100] | 2009 | | Japan | |  | | National survey | | Nationwide survey | Retrospective survey (onset <16 years) | Wingerchuk 2006 | Not stated | 50.0 | Not estimated | 10 | | 8 | | 2 | 10.3 ± 1.2  9.5 (3 - 15) | | Yes | | | Yes | | | No | | | Yes | | | Yes | | | Yes | | Unclear | Yes | No |
| 77 | 1 | Yoon et al., 2021 [101] | 2017 | | Korea | |  | | Case series | | National survey | National Health Insurance database (age <18 years) | ICD-10 G360 | Not stated | Not stated | Not estimated | 68 | | 42 | | 26 | 11.6 ± | | Yes | | | Yes | | | Yes | | | Yes | | | Yes | | | Yes | | Unclear | Yes | No |
| 78 | 1 | Zhou et al., 2019 [102] | 2017 | | Guangzhou, China | |  | | Case series | | Two centres | Retrospective survey (onset <18 years) | Wingerchuk 2006 plus AQP4 antibody positive | In-house live cell-based assay (precise details could not be located even after following quoted reference and supplementary data) | 100 | Not estimated | 23 | | 22 | | 1 | 13.8 ± 1.8^‡^  14 (10 - 17) | | Yes | | | Yes | | | No | | | Yes | | | Yes | | | Yes | | Unclear | Yes | No |
| ***Late Onset Studies*** | | |  | |  | |  | |  | |  |  |  |  |  |  |  | | |  |  |  |  | | |  | | |  | | |  | | |  | | |  | | |  |  |  |
| 79 | 1 | Cai et al., 2020 [103] | 2020 | | Chengdu, China | |  | | Case series | | Single centre | Retrospective casenote review (onset >50 years) | IPND 2015 | Euroimmun AQP4 transfected fixed cell-based assay | 85.3 | Not estimated | 116 | | 100 | | 16 | 58.2 ± 8.5^‡^  (52 - 76) | | Yes | | | Yes | | | Yes | | | Yes | | | Yes | | | Yes | | Yes | Yes | No |
| 80 | 1 | Carnero Contentti et al., 2020 [104] | 2020 | | Argentina, Brazil and Venezuela | |  | | Case series | | Multinational, multicentre | Retrospective casenote review (onset >49 years) | IPND 2015 | Cell-based assay (not further defined) or tissue indirect immunofluoresence | 66.7 | Not estimated | 24 | | 20 | | 4 | 59.8 ± 6.8 | | Yes | | | Unclear | | | Yes | | | Yes | | | Yes | | | Yes | | Unclear | Yes | No |
| 81 | 1 | Collongues et al., 2014 [105] | 2012 | | Europe | |  | | Case series | | Multinational, multicentre | Retrospective casenote review (onset >50 years) | Wingerchuk 2007 | M23 AQP4 transfected HEK293 live cell-based assay, radioimmunoprecipitation assay or indirect immunofluorescence assay | 85.0 | Not estimated | 108 | | 88 | | 20 | 56.6 (50 - 82) | | Yes | | | Yes | | | Yes | | | Yes | | | Yes | | | Yes | | Yes | Yes | No |
|  | 2 |  |  | |  | |  | |  | |  |  |  |  | 100 |  | 46 | | 41 | | 5 | 61.0 ± 7.2^‡^  56 (50 - 82) | | Yes | | | Yes | | | Yes | | | Yes | | | Yes | | | Yes | | Yes | Yes | No |
| 82 | 1 | Delgado-Garcia et al., 2020 [106] | 2019 | | Mexico | |  | | Case series | | Multinational, multicentre | Retrospective casenote review (onset >49 years) | AQP4 antibody positive | Not stated | 100 | Not estimated | 7 | | 7 | | 0 | 54.5 ± 4.7 | | Yes | | | Yes | | | No | | | Yes | | | Yes | | | Yes | | Unclear | Yes | No |
| 83 | 1 | Fragoso et al., 2019 [12] | 2019 | | Brazil | |  | | Case series | | Multicentre | Retrospective casenote review (onset >50 years) | IPND 2015 | Immunofluorescence technique (not otherwise stated) | 64.9 | Not estimated | 37 | | 30 | | 7 | 56.4 ± 5.6 | | Yes | | | Unclear | | | Yes | | | Yes | | | Yes | | | Yes | | Unclear | Yes | No |
| 84 | 1 | Lavandier et al., 2019 [107] | 2019 | | France | |  | | Case series | | Multicentre | Retrospective casenote review (onset >70 years) | IPND 2015 | Not stated | 83.3 | Not estimated | 6 | | 5 | | 1 | 74.3 ± 4.3  73.5 (71 - 81) | | Yes | | | Unclear | | | Yes | | | Yes | | | Yes | | | Yes | | Unclear | Yes | No |
|  | 2 |  |  | |  | |  | |  | |  |  |  |  | 100 |  | 5 | | 4 | | 1 | 74.2 ± 4.3  72 (71 - 81) | | Yes | | | Unclear | | | Yes | | | Yes | | | Yes | | | Yes | | Unclear | Yes | No |
| 85 | 1 | Mao et al., 2015 [108] | 2014 | | Guangdong, China | |  | | Case series | | Single centre | Retrospective chart review of consecutive cases (onset >49 years) | Wingerchuk 2006 plus AQP4 antibody positive | Euroimmun AQP4 transfected fixed cell-based assay | 100 | Not estimated | 30 | | 26 | | 4 | 58.8 ± 4.9^‡^  57.5 (50 - 70) | | Yes | | | Yes | | | Yes | | | Yes | | | Yes | | | Yes | | Yes | Yes | No |
| 86 | 1 | Nakahara et al., 2021 [109] | 2020 | | Kunamoto, Japan | |  | | Case series | | Single centre | Retrospective casenote review (onset >49 years) | IPND 2015 | Cell-based assay (not further defined) or ELISA | 90.0 | Not estimated | 30 | | 24 | | 6 | 60.7 ± 8.6^‡^  (51 - 72) | | Yes | | | Yes | | | Yes | | | Yes | | | Yes | | | Yes | | Unclear | Yes | No |
| 87 | 1 | Seok et al., 2017 [110] | 2014 | | Korea | |  | | Case series | | Multicentre | Retrospective casenote review (onset >49 years) | IPND 2015 plus AQP4 antibody positive | Euroimmun AQP4 transfected fixed cell-based assay | 100 | Not estimated | 45 | | 40 | | 5 | 58.1 ± 6.2 | | Yes | | | Yes | | | Yes | | | Yes | | | Yes | | | Yes | | Yes | Yes | No |
| 88 | 1 | Sepulveda et al., 2019 [111] | 2018 | | Spain | |  | | Case series | | Multicentre | Retrospective casenote review (onset >49 years) | IPND 2015 | M23 AQP4 transfected HEK293 live cell-based assay | 100 | Not estimated | 60 | | 48 | | 12 | 63.0 ± 8.3^‡^  59 (50 - 84) | | Yes | | | Yes | | | Yes | | | Yes | | | Yes | | | Yes | | Yes | Yes | No |
| 89 | 1 | Zhang et al., 2017 [112] | 2014 | | Tianjin, China | |  | | Case series | | Single centre | Retrospective casenote review (onset >50 years) | IPND 2015 | AQP4 transfected HEK293 cell-based assay or fluorescence immunoprecipitation assay | 88.1 | Not estimated | 59 | | 47 | | 12 | 59.4 ±  (51 - 80) | | Yes | | | Yes | | | Yes | | | Yes | | | Yes | | | Yes | | Yes | Yes | No |

No. = study number; Seq = sequence for serial studies within one article; AQP4 = aquaporin-4; IPND = International Panel for Neuromyelitis Optica Diagnosis; ICD = International Classification of Diseases: HEK = human embryonic kidney;

* = study also provided age of onset distribution data

^§^ = please note that this study is listed in PubMed by the authors first name “Viktoria et al., 2021” and the original article is also incorrect

^†^ = calculated from raw data provided in manuscript or supplementary data

^‡^ = estimated from summary data provided as detailed in methods

^#^ = standard deviation estimated from range

**Supplementary Table 2. List of excluded studies and reason for exclusion.**

| Study [Ref] | Reason for exclusion (prior/subsequent reference) [ref] |
| --- | --- |
| Alvarenga et al., 2017 [116] | Review of prior studies |
| Asgari et al., 2019 [117] | Review of prior studies |
| Ashtari et al., 2017 [118] | Subsequently updated (Shaygannejad et al., 2018) [81] |
| Badihian et al., 2018 [119] | No relevant data |
| Bergamaschi & Ghezzi, 2004 [120] | Review article |
| Bukhari et al., 2022 [121] | Previously described population (Bukhari et al., 2017) [37] |
| Cabre et al., 2009 [122] | Non-English |
| Carnero Contentti et al., 2020 [123] | No relevant data |
| Cheng et al., 2008 [124] | Not NMOSD population (MS) |
| Choy et al., 2018 [125] | Selected population (ON) |
| Collongues et al., 2011 [126] | Not NMOSD population ("high risk for NMO") |
| Cristiano et al., 2016 [127] | Not NMOSD population (MS) |
| Dale et al., 2018 [128] | No relevant data |
| Domingos et al., 2015 [129] | Subsequently updated (Santos et al., 2021) [3] |
| Eskandarieh et al., 2017 [130] | Previously described population (Eskandarieh et al., 2017) [47] |
| Eskandarieh et al., 2018 [131] | Previously described population (Eskandarieh et al., 2017) [47] |
| Etemadifar et al., 2014 [132] | Subsequently updated (Etemadifar et al., 2020) [49] |
| Etemadifar et al., 2014 [133] | Subsequently updated (Etemadifar et al., 2020) [49] |
| Fragoso et al., 2019 [134] | Selected population (early onset) |
| Gold et al., 2019 [135] | Review article |
| Höftberger et al., 2015 [136] | Subsequently updated (Sepulveda et al., 2016) [80] |
| Hor et al., 2020 [8] | Review article |
| Houzen et al., 2012 [137] | Subsequently updated (Miyamoto et al., 2018) [69] |
| Houzen et al., 2018 [138] | Not NMOSD population (MS) |
| Jacob et al., 2013 [139] | No relevant data |
| Jarius et al., 2010 [140] | Selected population (AQP4 Ab +ve ON) |
| Jarius et al., 2016 [141] | Not AQP4 Ab related NMOSD population (MOG Ab +ve) |
| Kim et al., 2018 [142] | Previously described population (Kim et al., 2012) [62] and data presented based on ethnic ancestry |
| Kim et al., 2020 [143] | No relevant data |
| Krumbholz et al., 2015 [144] | Case report |
| Lee et al., 2015 [145] | Selected population (Asian ancestry) |
| Lee et al., 2020 [146] | No relevant data |
| Li et al., 2021 [147] | Selected population (highly active NMSOD) |
| Marignier et al., 2017 [148] | Review article |
| Mealy et al., 2012 [149] | Subsequently updated (Mealy et al., 2018) [66] |
| O'Connell et al., 2020 [150] | No relevant data |
| Ortiz Salas et al., 2022 [151] | Non-English |
| Papp et al., 2018 [152] | Subsequently updated (Papp et al., 2021b) [74] |
| Park et al., 2014 [153] | Previously described population (Kim et al., 2012) [62] |
| Pittock et al., 2014 [154] | Selected population (previously diagnosed as MS) |
| Rezaeimanesh et al., 2020 [155] | No relevant data |
| Sahraian et al., 2010 [156] | Subsequently update (Eskandarieh et al., 2017) [47] |
| Saiz et al., 2007 [157] | Subsequently updated (Sepulveda et al., 2016) [80] |
| Sepulveda et al., 2018 [158] | Previously described population (Sepulveda et al., 2016) [80] |
| Simaniv et al., 2021 [159] | Non-English |
| Vanikieti et al., 2017 [160] | Selected population (NMOSD with ON) |
| Wang et al., 2011 [161] | Selected population (genetic analysis of NMOSD) |
| Wingerchuk et al., 2009 [9] | Review article |

**Supplementary Table 3. Age of onset distribution data for NMOSD**

|  |  |  | **Age Range (Years)** | | | | | | | | |  |
| --- | --- | --- | --- | --- | --- | --- | --- | --- | --- | --- | --- | --- |
| **Study [Ref]** | **Year** | **Region** | **0-9** | **10-19** | **20-29** | **30-39** | **40-49** | **50-59** | **60-69** | **70-79** | **80+** | **Total** |
| Cabrera-Gomez et al., 2009 [39] | 2004 | Cuba | 0 | 2 | 5 | 17 | 18 | 13 | 3 | 0 | 0 | 58 |
| Bizzoco et al 2009 [36] | 2006 | Italy | 1 | 0 | 5 | 1 | 3 | 2 | 1 | 0 | 0 | 13 |
| Cossburn et al., 2012 [43] | 2010 | UK | 2 | 3 | 0 | 1 | 4 | 1 | 2 | 1 | 0 | 14 |
| Adoni et al., 2010 [29] | 2010 | Brazil | 3 | 4 | 13 | 5 | 1 | 1 | 0 | 0 | 0 | 27 |
| Papais-Alvarenga et al., 2015 [72] | 2017 | Latin America | 10 | 36 | 69 | 48 | 38 | 22 | 3 | 0 | 0 | 226 |
| Bukhari et al., 2017 [37] | 2013 | Australia/New Zealand | 0 | 10 | 12 | 17 | 15 | 11 | 10 | 3 | 1 | 79 |
| Eskandarieh et al., 2017 [47] | 2016 | Iran | 4 | 15 | 37 | 26 | 18 | 2 | 1 | 0 | 0 | 103 |
| Tian et al., 2020a [87] | 2016 | China | 42 | 150 | 526 | 626 | 960 | 843 | 500 | 136 | 13 | 3796 |
| Tian et al., 2020b [87] | 2017 | China | 65 | 171 | 519 | 664 | 901 | 840 | 589 | 160 | 24 | 3933 |
| Tian et al., 2020c [87] | 2018 | China | 75 | 185 | 498 | 711 | 965 | 984 | 636 | 173 | 29 | 4256 |
| Shosha et al., 2020 [82] | 2020 | Arabian Gulf | 4 | 12 | 25 | 32 | 7 | 9 | 4 | 1 | 0 | 94 |
| **Combined** |  |  | **206** | **588** | **1709** | **2148** | **2930** | **2728** | **1749** | **474** | **67** | **12599** |

**Supplementary Table 4. Outcomes from meta-regression analysis of sex ratio for all studies**

| **Variable** | **Coefficient** | **(95% CI)** | **z** | **p** |
| --- | --- | --- | --- | --- |
| Intercept | 1.025 | (0.752 – 1.298) | 7.360 | <0.001 |
| Proportion AQP4 Antibody Positive | 0.008 | (0.005 – 0.011) | 5.411 | <0.001 |
| South/Central American* | 0.220 | (-0.001 – 0.440) | 1.951 | 0.051 |
| Middle Eastern/African* | 0.180 | (-0.072 – 0.432) | 1.397 | 0.162 |
| South Asian/Indian Subcontinent* | -0.283 | (-0.569 – 0.003) | -1.939 | 0.052 |
| South East Asian* | 0.225 | (-0.041 – 0.491) | 1.659 | 0.097 |

CI = confidence interval

* = Compared to White/European/Caucasian

**Supplementary Table 5. Outcomes from meta-regression analysis for age of onset**

| **Variable** | **Coefficient** | **(95% CI)** | **z** | **p** |
| --- | --- | --- | --- | --- |
| Intercept | -19.088 | (-44.557 – 6.381) | -1.469 | 0.142 |
| Mean Female Life-expectancy | 0.657 | (0.326 – 0.988) | 3.887 | <0.001 |
| Proportion AQP4 Antibody Positive | 0.053 | (0.009 – 0.096) | 2.343 | 0.019 |

CI = confidence interval

**Supplementary Table 6. Outcomes from meta-regression analysis of age of onset for AQP4 antibody positive cases only.**

| **Variable** | **Coefficient** | **(95% CI)** | **z** | **p** |
| --- | --- | --- | --- | --- |
| Intercept | -30.349 | (-64.257 – 3.559) | -1.754 | 0.079 |
| Mean Female Life-expectancy | 0.854 | (0.433 – 1.275) | 3.979 | <0.001 |

CI = confidence interval

**References**

1. Wingerchuk DM, Banwell B, Bennett JL, Cabre P, Carroll W, Chitnis T, de Seze J, Fujihara K, Greenberg B, Jacob A, Jarius S, Lana-Peixoto M, Levy M, Simon JH, Tenembaum S, Traboulsee AL, Waters P, Wellik KE, Weinshenker BG, International Panel for NMOD (2015) International consensus diagnostic criteria for neuromyelitis optica spectrum disorders. Neurology 85(2):177-89. https://doi.org/10.1212/WNL.0000000000001729

2. Hinson SR, Pittock SJ, Lucchinetti CF, Roemer SF, Fryer JP, Kryzer TJ, Lennon VA (2007) Pathogenic potential of IgG binding to water channel extracellular domain in neuromyelitis optica. Neurology 69(24):2221-31. https://doi.org/10.1212/01.WNL.0000289761.64862.ce

3. Santos E, Rocha AL, Oliveira V, Ferro D, Samoes R, Sousa AP, Figueiroa S, Mendonca T, Abreu P, Guimaraes J, Sousa R, Melo C, Correia I, Duraes J, Sousa L, Ferreira J, de Sa J, Sousa F, Sequeira M, Correia AS, Andre AL, Basilio C, Arenga M, Mendes I, Marques IB, Perdigao S, Felgueiras H, Alves I, Correia F, Barroso C, Morganho A, Carmona C, Palavra F, Santos M, Salgado V, Palos A, Nzwalo H, Timoteo A, Guerreiro R, Isidoro L, Boleixa D, Carneiro P, Neves E, Silva AM, Goncalves G, Leite MI, Sa MJ (2021) Neuromyelitis optica spectrum disorders: A nationwide Portuguese clinical epidemiological study. Mult Scler Relat Disord 56:103258. https://doi.org/10.1016/j.msard.2021.103258

4. Watanabe M, Nakamura Y, Sato S, Niino M, Fukaura H, Tanaka M, Ochi H, Kanda T, Takeshita Y, Yokota T, Nishida Y, Matsui M, Nagayama S, Kusunoki S, Miyamoto K, Mizuno M, Kawachi I, Saji E, Ohashi T, Shimohama S, Hisahara S, Nishiyama K, Iizuka T, Nakatsuji Y, Okuno T, Ochi K, Suzumura A, Yamamoto K, Kawano Y, Tsuji S, Hirata M, Sakate R, Kimura T, Shimizu Y, Nagaishi A, Okada K, Hayashi F, Sakoda A, Masaki K, Shinoda K, Isobe N, Matsushita T, Kira JI (2021) HLA genotype-clinical phenotype correlations in multiple sclerosis and neuromyelitis optica spectrum disorders based on Japan MS/NMOSD Biobank data. Sci Rep 11(1):607. https://doi.org/10.1038/s41598-020-79833-7

5. Frau J, Coghe G, Lorefice L, Fenu G, Cocco E (2023) The Role of Microorganisms in the Etiopathogenesis of Demyelinating Diseases. Life (Basel) 13(6):1309. https://doi.org/10.3390/life13061309

6. Liu S, Tan B, Zhou J, Xiao L, Li M, Yin J (2024) Vitamin D status and the risk of neuromyelitis optica spectrum disorders: A systematic review and meta-analysis. J Clin Neurosci 119:185-92. https://doi.org/10.1016/j.jocn.2023.12.010

7. Davey Smith G (2019) Post-Modern Epidemiology: When Methods Meet Matter. Am J Epidemiol 188(8):1410-9. https://doi.org/10.1093/aje/kwz064

8. Hor JY, Asgari N, Nakashima I, Broadley SA, Leite MI, Kissani N, Jacob A, Marignier R, Weinshenker BG, Paul F, Pittock SJ, Palace J, Wingerchuk DM, Behne JM, Yeaman MR, Fujihara K (2020) Epidemiology of Neuromyelitis Optica Spectrum Disorder and Its Prevalence and Incidence Worldwide. Front Neurol 11:501. https://doi.org/10.3389/fneur.2020.00501

9. Wingerchuk DM (2009) Neuromyelitis optica: effect of gender. J Neurol Sci 286(1-2):18-23. https://doi.org/10.1016/j.jns.2009.08.045

10. Pandit L, Asgari N, Apiwattanakul M, Palace J, Paul F, Leite MI, Kleiter I, Chitnis T, Consortium GIC, Biorepository for Neuromyelitis O (2015) Demographic and clinical features of neuromyelitis optica: A review. Mult Scler 21(7):845-53. https://doi.org/10.1177/1352458515572406

11. Chitnis T, Ness J, Krupp L, Waubant E, Hunt T, Olsen CS, Rodriguez M, Lotze T, Gorman M, Benson L, Belman A, Weinstock-Guttman B, Aaen G, Graves J, Patterson M, Rose JW, Casper TC (2016) Clinical features of neuromyelitis optica in children: US Network of Pediatric MS Centers report. Neurology 86(3):245-52. https://doi.org/10.1212/WNL.0000000000002283

12. Fragoso YD, Ruocco HH, Dias RM, Cabeca H, Goncalves R, de Carvalho Sousa NA, Spessotto CV, Tauil CB, Alves-Leon SV, Gomes S, Goncalves MVM, Machado SCN, Anacleto A, Correa EC, Pimentel MLV, Santos GAC (2019) Late Onset of Neuromyelitis Optica Spectrum Disorders. Neurol Ther 8(2):477-82. https://doi.org/10.1007/s40120-019-0143-2

13. Wingerchuk DM, Hogancamp WF, O'Brien PC, Weinshenker BG (1999) The clinical course of neuromyelitis optica (Devic's syndrome). Neurology 53(5):1107-14. https://doi.org/10.1212/wnl.53.5.1107

14. Wingerchuk DM, Lennon VA, Pittock SJ, Lucchinetti CF, Weinshenker BG (2006) Revised diagnostic criteria for neuromyelitis optica. Neurology 66(10):1485-9. https://doi.org/10.1212/01.wnl.0000216139.44259.74

15. Wingerchuk DM, Lennon VA, Lucchinetti CF, Pittock SJ, Weinshenker BG (2007) The spectrum of neuromyelitis optica. Lancet Neurol 6(9):805-15. https://doi.org/10.1016/S1474-4422(07)70216-8

16. Banwell B, Bennett JL, Marignier R, Kim HJ, Brilot F, Flanagan EP, Ramanathan S, Waters P, Tenembaum S, Graves JS, Chitnis T, Brandt AU, Hemingway C, Neuteboom R, Pandit L, Reindl M, Saiz A, Sato DK, Rostasy K, Paul F, Pittock SJ, Fujihara K, Palace J (2023) Diagnosis of myelin oligodendrocyte glycoprotein antibody-associated disease: International MOGAD Panel proposed criteria. Lancet Neurol 22(3):268-82. https://doi.org/10.1016/S1474-4422(22)00431-8

17. Boudjani H, Fadda G, Dufort G, Antel J, Giacomini P, Levesque-Roy M, Oskoui M, Duquette P, Prat A, Girard M, Rebillard RM, Meijer I, Pinchefsky E, Nguyen CE, Rossignol E, Rouleau J, Blanchard O, Khairallah N, Beauchemin P, Trudelle AM, Lapointe E, Saveriano A, Larochelle C (2023) Clinical course, imaging, and pathological features of 45 adult and pediatric cases of myelin oligodendrocyte glycoprotein antibody-associated disease. Mult Scler Relat Disord 76:104787. https://doi.org/10.1016/j.msard.2023.104787

18. Modig K, Rau R, Ahlbom A (2020) Life expectancy: what does it measure? BMJ Open 10(7):e035932. https://doi.org/10.1136/bmjopen-2019-035932

19. World Health Organization (2020) Life expectancy and health life expectancy data by country. Accessed 24 Oct 2023. World Health Organization, Geneva, Switzerland. https://www.who.int/data/gho/data/indicators/indicator-details/GHO/life-expectancy-at-birth-(years)

20. Munn Z, Moola S, Lisy K, Riitano D, Tufanaru C (2015) Methodological guidance for systematic reviews of observational epidemiological studies reporting prevalence and cumulative incidence data. Int J Evid Based Healthc 13(3):147-53. https://doi.org/10.1097/XEB.0000000000000054

21. Wan X, Wang W, Liu J, Tong T (2014) Estimating the sample mean and standard deviation from the sample size, median, range and/or interquartile range. BMC Med Res Methodol 14:135. https://doi.org/10.1186/1471-2288-14-135

22. Borenstein M, Hedges LV, Higgins JPT, Rothstein HR (2008) Comprehensive meta-analysis (version 2.2.027). Organ Res Methods 11(1):188-91. https://doi.org/10.1177/1094428106296641

23. Möller S, Ahrenfeldt LJ (2021) Estimating relative risk when observing zero events - frequentist inference and Bayesian credibility intervals. Int J Env Res Pub Health 18(11):5527. https://doi.org/10.3390/ijerph18115527

24. Higgins JP, Thompson SG, Deeks JJ, Altman DG (2003) Measuring inconsistency in meta-analyses. British Medical Journal 327(7414):557-60. https://doi.org/10.1136/bmj.327.7414.557

25. Egger M, Davey Smith G, Schneider M, Minder C (1997) Bias in meta-analysis detected by a simple, graphical test. British Medical Journal 315(7109):629-34. https://doi.org/10.1136/bmj.315.7109.629

26. Naing NN (2000) Easy way to learn standardization : direct and indirect methods. Malays J Med Sci 7(1):10-5. PMC3406211

27. United Nations (2022) World Population Prospects: The 2022 Revision. Accessed 24 Oct 2023. United Nations, Department of Economic and Social Affairs, Population Division, New York, NY, USA. https://www.populationpyramid.net/

28. Aboul-Enein F, Seifert-Held T, Mader S, Kuenz B, Lutterotti A, Rauschka H, Rommer P, Leutmezer F, Vass K, Flamm-Horak A, Stepansky R, Lang W, Fertl E, Schlager T, Heller T, Eggers C, Safoschnik G, Fuchs S, Kraus J, Assar H, Guggenberger S, Reisz M, Schnabl P, Komposch M, Simschitz P, Skrobal A, Moser A, Jeschow M, Stadlbauer D, Freimuller M, Guger M, Schmidegg S, Franta C, Weiser V, Koppi S, Niederkorn-Duft M, Raber B, Schmeissner I, Jecel J, Tinchon A, Storch MK, Reindl M, Berger T, Kristoferitsch W (2013) Neuromyelitis optica in Austria in 2011: to bridge the gap between neuroepidemiological research and practice in a study population of 8.4 million people. PLoS One 8(11):e79649. https://doi.org/10.1371/journal.pone.0079649

29. Adoni T, Lino AM, da Gama PD, Apostolos-Pereira SL, Marchiori PE, Kok F, Callegaro D (2010) Recurrent neuromyelitis optica in Brazilian patients: clinical, immunological, and neuroimaging characteristics. Mult Scler 16(1):81-6. https://doi.org/10.1177/1352458509353651

30. Altintas A, Karabudak R, Balci BP, Terzi M, Soysal A, Saip S, Tuncer Kurne A, Uygunoglu U, Nalbantoglu M, Gozubatik Celik G, Isik N, Celik Y, Gokcay F, Duman T, Boz C, Yucesan C, Mangan MS, Celebisoy N, Diker S, Colpak Isikay I, Kansu T, Siva A (2015) Neuromyelitis Optica and Neuromyelitis Optica Spectrum Disorder Patients in Turkish Cohort: Demographic, Clinical, and Laboratory Features. Neurologist 20(4):61-6. https://doi.org/10.1097/NRL.0000000000000057

31. Alves CS, Santos FBC, Diniz DS (2022) Correlation between Amerindian ancestry and neuromyelitis optica spectrum disorders (NMSOD) among patients in Midwestern Brazil. Arq Neuropsiquiatr 80(5):497-504. https://doi.org/10.1590/0004-282X-ANP-2020-0527

32. Asgari N, Lillevang ST, Skejoe HP, Falah M, Stenager E, Kyvik KO (2011) A population-based study of neuromyelitis optica in Caucasians. Neurology 76(18):1589-95. https://doi.org/10.1212/WNL.0b013e3182190f74

33. Barhate KS, Ganeshan M, Singhal BS (2014) A clinical and radiological profile of neuromyelitis optica and spectrum disorders in an Indian cohort. Ann Indian Acad Neurol 17(1):77-81. https://doi.org/10.4103/0972-2327.128559

34. Bennis A, El Otmani H, Benkirane N, Harrizi I, El Moutawakil B, Rafai MA, Slassi I (2019) Clinical course of neuromyelitis optica spectrum disorder in a moroccan cohort. Mult Scler Relat Disord 30:141-8. https://doi.org/10.1016/j.msard.2019.02.012

35. Bichuetti DB, Oliveira EM, Souza NA, Rivero RL, Gabbai AA (2009) Neuromyelitis optica in Brazil: a study on clinical and prognostic factors. Mult Scler 15(5):613-9. https://doi.org/10.1177/1352458508101935

36. Bizzoco E, Lolli F, Repice AM, Hakiki B, Falcini M, Barilaro A, Taiuti R, Siracusa G, Amato MP, Biagioli T, Lori S, Moretti M, Vinattieri A, Nencini P, Massacesi L, Mata S (2009) Prevalence of neuromyelitis optica spectrum disorder and phenotype distribution. J Neurol 256(11):1891-8. https://doi.org/10.1007/s00415-009-5171-x

37. Bukhari W, Prain KM, Waters P, Woodhall M, O'Gorman CM, Clarke L, Silvestrini RA, Bundell CS, Abernethy D, Bhuta S, Blum S, Boggild M, Boundy K, Brew BJ, Brown M, Brownlee WJ, Butzkueven H, Carroll WM, Chen C, Coulthard A, Dale RC, Das C, Dear K, Fabis-Pedrini MJ, Fulcher D, Gillis D, Hawke S, Heard R, Henderson APD, Heshmat S, Hodgkinson S, Jimenez-Sanchez S, Killpatrick T, King J, Kneebone C, Kornberg AJ, Lechner-Scott J, Lin MW, Lynch C, Macdonell R, Mason DF, McCombe PA, Pender MP, Pereira JA, Pollard JD, Reddel SW, Shaw C, Spies J, Stankovich J, Sutton I, Vucic S, Walsh M, Wong RC, Yiu EM, Barnett MH, Kermode AG, Marriott MP, Parratt JDE, Slee M, Taylor BV, Willoughby E, Wilson RJ, Vincent A, Broadley SA (2017) Incidence and prevalence of NMOSD in Australia and New Zealand. J Neurol Neurosurg Psychiatry 88(8):632-8. https://doi.org/10.1136/jnnp-2016-314839

38. Cabre P, Heinzlef O, Merle H, Buisson GG, Bera O, Bellance R, Vernant JC, Smadja D (2001) MS and neuromyelitis optica in Martinique (French West Indies). Neurology 56(4):507-14. https://doi.org/10.1212/wnl.56.4.507

39. Cabrera-Gomez JA, Kurtzke JF, Gonzalez-Quevedo A, Lara-Rodriguez R (2009) An epidemiological study of neuromyelitis optica in Cuba. J Neurol 256(1):35-44. https://doi.org/10.1007/s00415-009-0009-0

40. Ceccarelli A, Mifsud VA, Dogar A, Hussain SI (2020) Seropositive neuromyelitis optica spectrum disorder in Emirati patients: A case series. J Clin Neurosci 72:185-90. https://doi.org/10.1016/j.jocn.2019.11.045

41. Chan KH, Lee R, Lee JC, Tse AC, Pang SY, Lau GK, Teo KC, Ho PW (2013) Central nervous system inflammatory demyelinating disorders among Hong Kong Chinese. J Neuroimmunol 262(1-2):100-5. https://doi.org/10.1016/j.jneuroim.2013.06.004

42. Collongues N, Marignier R, Zephir H, Papeix C, Blanc F, Ritleng C, Tchikviladze M, Outteryck O, Vukusic S, Fleury M, Fontaine B, Brassat D, Clanet M, Milh M, Pelletier J, Audoin B, Ruet A, Lebrun-Frenay C, Thouvenot E, Camu W, Debouverie M, Creange A, Moreau T, Labauge P, Castelnovo G, Edan G, Le Page E, Defer G, Barroso B, Heinzlef O, Gout O, Rodriguez D, Wiertlewski S, Laplaud D, Borgel F, Tourniaire P, Grimaud J, Brochet B, Vermersch P, Confavreux C, de Seze J (2010) Neuromyelitis optica in France: a multicenter study of 125 patients. Neurology 74(9):736-42. https://doi.org/10.1212/WNL.0b013e3181d31e35

43. Cossburn M, Tackley G, Baker K, Ingram G, Burtonwood M, Malik G, Pickersgill T, te Water Naude J, Robertson N (2012) The prevalence of neuromyelitis optica in South East Wales. Eur J Neurol 19(4):655-9. https://doi.org/10.1111/j.1468-1331.2011.03529.x

44. Daoudi S, Bouzar M (2016) Neuromyelitis optica spectrum disorders in Algeria: A preliminary study in the region of Tizi Ouzou. Mult Scler Relat Disord 6:37-40. https://doi.org/10.1016/j.msard.2015.12.005

45. Del Negro MC, Marinho PB, Papais-Alvarenga RM (2017) Neuromyelitis optica: phenotypic characteristics in a Brazilian case series. Arq Neuropsiquiatr 75(2):81-6. https://doi.org/10.1590/0004-282X20160193

46. Drulovic J, Martinovic V, Basuroski ID, Mesaros S, Mader S, Weinshenker B, Pekmezovic T (2019) Long-term outcome and prognosis in patients with neuromyelitis optica spectrum disorder from Serbia. Mult Scler Relat Disord 36:101413. https://doi.org/10.1016/j.msard.2019.101413

47. Eskandarieh S, Nedjat S, Azimi AR, Moghadasi AN, Sahraian MA (2017) Neuromyelitis optica spectrum disorders in Iran. Mult Scler Relat Disord 18:209-12. https://doi.org/10.1016/j.msard.2017.10.007

48. Espiritu AI, Mesina BVQ, Puerto AAD, Reyes NGD, Damian LF, Pascual VJ (2019) Neuromyelitis optica spectrum disorder in a tertiary hospital in the Philippines: a case series. Mult Scler Relat Disord 31:124-30. https://doi.org/10.1016/j.msard.2019.04.006

49. Etemadifar M, Sabeti F, Ebrahimian S, Momeni F (2020) Dorsal midbrain involvement in MRI as a core clinical manifestation for NMOSD diagnosis. Mult Scler Relat Disord 43:102150. https://doi.org/10.1016/j.msard.2020.102150

50. Flanagan EP, Cabre P, Weinshenker BG, St Sauver J, Jacobson DJ, Majed M, Lennon VA, Lucchinetti CF, McKeon A, Matiello M, Kale N, Wingerchuk DM, Mandrekar J, Sagen JA, Fryer JP, Borders Robinson A, Pittock SJ (2016) Epidemiology of aquaporin-4 autoimmunity and neuromyelitis optica spectrum. Ann Neurol 79(5):775-83. https://doi.org/10.1002/ana.24617

51. Fukuda TG, Silva ITF, Dos Santos TSS, Filho MBP, de Abreu FF, Oliveira-Filho J (2022) Clinical and prognostic aspects of patients with the Neuromyelitis Optica Spectrum Disorder (NMOSD) from a cohort in Northeast Brazil. BMC Neurol 22(1):95. https://doi.org/10.1186/s12883-022-02621-5

52. Gao M, Yao X, Ding J, Hong R, Wu Y, Huang H, Zhuang L, Li Z, Wang Y, Zhang Y, Guan Y (2019) Low levels of vitamin D and the relationship between vitamin D and Th2 axis-related cytokines in neuromyelitis optica spectrum disorders. J Clin Neurosci 61:22-7. https://doi.org/10.1016/j.jocn.2018.11.024

53. Gracia F, Ramirez D, Parajeles-Vindas A, Diaz A, Diaz de la Fe A, Sanchez NER, Escobar RC, Valle LAG, Weiser R, Santos B, Candelario A, Benzadon A, Araujo P, Valderrama C, Larreategui M, Carrillo G, Gracia K, Vazquez-Cespedes J, Monterrey-Alvarez P, Carazo-Cespedes K, Sanabria-Castro A, Miranda-Loria G, Balmaceda-Meza A, Rivera LIP, Leal IO, Salinas LCR, Thompson A, Torres EL, Pereira DE, Zepeda C, Lopez CA, Valse EAC, Urbina KZC, Urrutia MA, Van Sijtveld I, Armien B, Rivera VM (2022) Neuromyelitis Optica Spectrum Disorder in Central America and the Caribbean: A Multinational Clinical Characterization Study. Neurol Int 14(1):284-93. https://doi.org/10.3390/neurolint14010023

54. Holroyd KB, Aziz F, Szolics M, Alsaadi T, Levy M, Schiess N (2018) Prevalence and characteristics of transverse myelitis and neuromyelitis optica spectrum disorders in the United Arab Emirates: A multicenter, retrospective study. Clin Exp Neuroimmunol 9(3):155-61. https://doi.org/10.1111/cen3.12458

55. Hor JY, Lim TT, Chia YK, Ching YM, Cheah CF, Tan K, Chow HB, Arip M, Eow GB, Easaw PES, Leite MI (2018) Prevalence of neuromyelitis optica spectrum disorder in the multi-ethnic Penang Island, Malaysia, and a review of worldwide prevalence. Mult Scler Relat Disord 19:20-4. https://doi.org/10.1016/j.msard.2017.10.015

56. Ibis SC, Omaira M, Arnoldo S, Elizabeth A, Sandra M, Carlota CM, Elizabeth C, Laura V, Rosalba L, Oscar V, Luisa DM, Nahir A, Hernandez F (2021) Epidemiological findings of neuromyelitis optica spectrum disorders in a Venezuelan study. Mult Scler Relat Disord 47:102652. https://doi.org/10.1016/j.msard.2020.102652

57. Jagtap SA, Mandliya A, Sarada C, Nair MD (2015) Neuromyelitis optica and neuromyelitis optica spectrum disorder: Natural history and long-term outcome, an Indian experience. J Neurosci Rural Pract 6(3):331-5. https://doi.org/10.4103/0976-3147.158755

58. Jiao Y, Fryer JP, Lennon VA, Jenkins SM, Quek AM, Smith CY, McKeon A, Costanzi C, Iorio R, Weinshenker BG, Wingerchuk DM, Shuster EA, Lucchinetti CF, Pittock SJ (2013) Updated estimate of AQP4-IgG serostatus and disability outcome in neuromyelitis optica. Neurology 81(14):1197-204. https://doi.org/10.1212/WNL.0b013e3182a6cb5c

59. Jonsson DI, Sveinsson O, Hakim R, Brundin L (2019) Epidemiology of NMOSD in Sweden from 1987 to 2013: A nationwide population-based study. Neurology 93(2):e181-e9. https://doi.org/10.1212/WNL.0000000000007746

60. Joshi PB, Shah SD, Patel MA, Shah SV, Darji SH, Mirche KC (2022) A Study of Neuromyelitis Optica Spectrum Disorders (NMOSD): Disease Pattern Based on Antibody Status. Neurol India 70(3):1131-6. https://doi.org/10.4103/0028-3886.349679

61. Kashipazha D, Mohammadianinejad SE, Majdinasab N, Azizi M, Jafari M (2015) A descriptive study of prevalence, clinical features and other findings of neuromyelitis optica and neuromyelitis optica spectrum disorder in Khuzestan Province, Iran. Iran J Neurol 14(4):204-10. PMC4754599

62. Kim SH, Kim W, Li XF, Jung IJ, Kim HJ (2012) Clinical spectrum of CNS aquaporin-4 autoimmunity. Neurology 78(15):1179-85. https://doi.org/10.1212/WNL.0b013e31824f8069

63. Kishk NA, Abdelfattah W, Shalaby NM, Shehata HS, Hassan A, Hegazy MI, Abokrysha NT, Abdellatif D, Shawky SM, Abdo SS, Taha N, Fouad AM, Elmazny A, Ragab AH (2021) The aquaporin4-IgG status and how it affects the clinical features and treatment response in NMOSD patients in Egypt. BMC Neurol 21(1):53. https://doi.org/10.1186/s12883-021-02083-1

64. Kitley J, Waters P, Woodhall M, Leite MI, Murchison A, George J, Kuker W, Chandratre S, Vincent A, Palace J (2014) Neuromyelitis optica spectrum disorders with aquaporin-4 and myelin-oligodendrocyte glycoprotein antibodies: a comparative study. JAMA Neurol 71(3):276-83. https://doi.org/10.1001/jamaneurol.2013.5857

65. Kleiter I, Gahlen A, Borisow N, Fischer K, Wernecke KD, Wegner B, Hellwig K, Pache F, Ruprecht K, Havla J, Krumbholz M, Kumpfel T, Aktas O, Hartung HP, Ringelstein M, Geis C, Kleinschnitz C, Berthele A, Hemmer B, Angstwurm K, Stellmann JP, Schuster S, Stangel M, Lauda F, Tumani H, Mayer C, Zeltner L, Ziemann U, Linker R, Schwab M, Marziniak M, Then Bergh F, Hofstadt-van Oy U, Neuhaus O, Winkelmann A, Marouf W, Faiss J, Wildemann B, Paul F, Jarius S, Trebst C, Neuromyelitis Optica Study G (2016) Neuromyelitis optica: Evaluation of 871 attacks and 1,153 treatment courses. Ann Neurol 79(2):206-16. https://doi.org/10.1002/ana.24554

66. Mealy MA, Kessler RA, Rimler Z, Reid A, Totonis L, Cutter G, Kister I, Levy M (2018) Mortality in neuromyelitis optica is strongly associated with African ancestry. Neurol Neuroimmunol Neuroinflamm 5(4):e468. https://doi.org/10.1212/NXI.0000000000000468

67. Mireles-Ramirez MA, Pacheco-Moises FP, Gonzalez-Usigli HA, Sanchez-Rosales NA, Hernandez-Preciado MR, Delgado-Lara DLC, Hernandez-Cruz JJ, Ortiz GG (2022) Neuromyelitis optica spectrum disorder: pathophysiological approach. Int J Neurosci:1-13. https://doi.org/10.1080/00207454.2022.2153046

68. Mirmosayyeb O, Barzegar M, Afshari-Safavi A, Nehzat N, Heidari A, Emami P, Shaygannejad V (2021) Evaluation of Month of Birth in Neuromyelitis Optica Spectrum Disorders (NMSOD) and Multiple Sclerosis (MS). Mult Scler Int 2021:8874999. https://doi.org/10.1155/2021/8874999

69. Miyamoto K, Fujihara K, Kira JI, Kuriyama N, Matsui M, Tamakoshi A, Kusunoki S (2018) Nationwide epidemiological study of neuromyelitis optica in Japan. J Neurol Neurosurg Psychiatry 89(6):667-8. https://doi.org/10.1136/jnnp-2017-317321

70. Netravathi M, Bollampalli HK, Bhat MD, Ganaraja VH, Prasad S, Mahadevan A, Kamble N, Nalini A, Yadav R, Pal PK, Satishchandra P (2019) Clinical, neuroimaging and therapeutic response in AQP4-positive NMO patients from India. Mult Scler Relat Disord 30:85-93. https://doi.org/10.1016/j.msard.2019.01.032

71. Pandit L, Kundapur R (2014) Prevalence and patterns of demyelinating central nervous system disorders in urban Mangalore, South India. Mult Scler 20(12):1651-3. https://doi.org/10.1177/1352458514521503

72. Papais-Alvarenga RM, Vasconcelos CC, Carra A, de Castillo IS, Florentin S, Diaz de Bedoya FH, Mandler R, de Siervi LC, Pimentel ML, Alvarenga MP, Alvarenga MP, Grzesiuk AK, Gama Pereira AB, Gomes Neto AP, Velasquez C, Soublette C, Fleitas CV, Diniz DS, Armas E, Batista E, Hernandez F, Pereira FF, Siqueira HH, Cabeca H, Sanchez J, Brooks JB, Goncalves MV, Barroso MC, Ravelo ME, Castillo MC, Ferreira ML, Rocha MS, Parolin MK, Molina O, Marinho PB, Christo PP, Brant de Souza R, Pessanha Neto S, Camargo SM, Machado SC, Neri VC, Fragoso YD, Alvarenga H, Thuler LC (2015) Central Nervous System Idiopathic Inflammatory Demyelinating Disorders in South Americans: A Descriptive, Multicenter, Cross-Sectional Study. PLoS One 10(7):e0127757. https://doi.org/10.1371/journal.pone.0127757

73. Papp V, Iljicsov A, Rajda C, Magyari M, Koch-Henriksen N, Petersen T, Jakab G, Deme I, Nagy F, Imre P, Lohner Z, Kovacs K, Birkas AJ, Koves A, Rum G, Nagy Z, Kerenyi L, Vecsei L, Bencsik K, Jobbagy Z, Dioszeghy P, Horvath L, Galantai G, Kasza J, Molnar G, Simo M, Satori M, Rozsa C, Acs P, Berki T, Lovas G, Komoly S, Illes Z (2020) A population-based epidemiological study of neuromyelitis optica spectrum disorder in Hungary. Eur J Neurol 27(2):308-17. https://doi.org/10.1111/ene.14079

74. Papp V, Trones KDP, Magyari M, Koch-Henriksen N, Iljicsov A, Rajda C, Nielsen HH, Lovas G, Rozsa C, Kristiansen BH, Stenager E, Frederiksen JL, Komoly S, Sellebjerg F, Petersen T, Illes Z (2021) Population-based head-to-head comparison of the clinical characteristics and epidemiology of AQP4 antibody-positive NMOSD between two European countries. Mult Scler Relat Disord 51:102879. https://doi.org/10.1016/j.msard.2021.102879

75. Paz ES, Maciel P, D'Almeida JAC, Silva B, Sampaio HAC, Pinheiro ADV, Carioca AAF, de Melo MLP (2021) Excess weight, central adiposity and pro-inflammatory diet consumption in patients with neuromyelitis optica spectrum disorder. Mult Scler Relat Disord 54:103110. https://doi.org/10.1016/j.msard.2021.103110

76. Rafiee F, Tarjoman T, Moghadasi AN, Sahraian MA, Azimi A, Rezaeimanesh N, Eskandarieh S (2020) Stressful life events, socioeconomic status, and the risk of neuromyelitis optica spectrum disorder: A population-based case-control study. Mult Scler Relat Disord 46:102544. https://doi.org/10.1016/j.msard.2020.102544

77. Rivera JF, Kurtzke JF, Booth VJ, Corona VTt (2008) Characteristics of Devic's disease (neuromyelitis optica) in Mexico. J Neurol 255(5):710-5. https://doi.org/10.1007/s00415-008-0781-2

78. Rojas JI, Alonso Serena M, Garcea O, Patrucco L, Carra A, Correale J, Vrech C, Pappolla A, Miguez J, Doldan ML, Silveira F, Alonso R, Cohen L, Pita C, Silva BA, Fiol M, Gaitan MI, Marrodan M, Negrotto L, Ysrraelit MC, Deri N, Luetic G, Caride A, Carnero Contentti E, Lopez PA, Pettinicchi JP, Curbelo C, Martinez AD, Steinberg JD, Balbuena ME, Tkachuk V, Burgos M, Knorre E, Leguizamon F, Piedrabuena R, Liwacki SDV, Barboza AG, Nofal P, Volman G, Alvez Pinheiro A, Hryb J, Tavolini D, Blaya PA, Silva E, Blanche J, Tizio S, Caceres F, Saladino ML, Zanga G, Fracaro ME, Sgrilli G, Pagani Cassara F, Vazquez G, Sinay V, Menichini ML, Lazaro L, Cabrera LM, Bestoso S, Divi P, Jacobo M, Kohler E, Kohler M, Giunta D, Mainella C, Manzi R, Parada Marcilla M, Viglione JP, Martos I, Reich E, Jose G, Cristiano E, Fernandez Liguori N, on behalf Relevar EMi (2020) Multiple sclerosis and neuromyelitis optica spectrum disorders in Argentina: comparing baseline data from the Argentinean MS Registry (RelevarEM). Neurol Sci 41(6):1513-9. https://doi.org/10.1007/s10072-019-04230-6

79. Salama S, Marouf H, Ihab Reda M, Mansour AR, O EL, Levy M (2018) Clinical and radiological characteristics of neuromyelitis optica spectrum disorder in the North Egyptian Nile Delta. J Neuroimmunol 324:22-5. https://doi.org/10.1016/j.jneuroim.2018.08.014

80. Sepulveda M, Armangue T, Sola-Valls N, Arrambide G, Meca-Lallana JE, Oreja-Guevara C, Mendibe M, Alvarez de Arcaya A, Aladro Y, Casanova B, Olascoaga J, Jimenez-Huete A, Fernandez-Fournier M, Ramio-Torrenta L, Cobo-Calvo A, Vinals M, de Andres C, Meca-Lallana V, Cervello A, Calles C, Rubio MB, Ramo-Tello C, Caminero A, Munteis E, Antiguedad AR, Blanco Y, Villoslada P, Montalban X, Graus F, Saiz A (2016) Neuromyelitis optica spectrum disorders: Comparison according to the phenotype and serostatus. Neurol Neuroimmunol Neuroinflamm 3(3):e225. https://doi.org/10.1212/NXI.0000000000000225

81. Shaygannejad V, Maljaei MB, Bank SS, Mirmosayyeb O, Maracy MR, Askari G (2018) Association between Sun Exposure, Vitamin D Intake, Serum Vitamin D Level, and Immunoglobulin G Level in Patients with Neuromyelitis Optica Spectrum Disorder. Int J Prev Med 9:68. https://doi.org/10.4103/ijpvm.IJPVM_45_16

82. Shosha E, Al Asmi A, Nasim E, Inshasi J, Abdulla F, Al Malik Y, Althobaiti A, Alzawahmah M, Alnajashi HA, Binfalah M, AlHarbi A, Thubaiti IA, Ahmed SF, Al-Hashel J, Elyas M, Nandhagopal R, Gujjar A, Harbi TA, Towaijri GA, Alsharooqi IA, AlMaawi A, Al Khathaami AM, Alotaibi N, Nahrir S, Al Rasheed AA, Al Qahtani M, Alawi S, Hundallah K, Jumah M, Alroughani R, with the Guthy-Jackson Charitable Foundation International Clinical C (2020) Neuromyelitis optica spectrum disorders in Arabian Gulf (NMOAG); establishment and initial characterization of a patient registry. Mult Scler Relat Disord 38:101448. https://doi.org/10.1016/j.msard.2019.101448

83. Singh N, Bhatia R, Bali P, Sreenivas V, Padma MV, Goyal V, Saxena R, Dash D, Garg A, Joseph SL (2021) Clinical Features, Gender Differences, Disease Course, and Outcome in Neuromyelitis Optica Spectrum Disorder. Ann Indian Acad Neurol 24(2):186-91. https://doi.org/10.4103/aian.AIAN_334_20

84. Stratos K, Lee L, Dai D, Pavenski K, Zuo F, Rotstein D (2020) Evaluation of ethnicity as a predictor of diagnostic phenotype and prognosis in neuromyelitis optica spectrum disorder in Toronto, Canada. Mult Scler Relat Disord 40:101950. https://doi.org/10.1016/j.msard.2020.101950

85. Sun H, Sun X, Li J, Huo Y, Wu L, Huang D, Yu S, Wu W (2017) Gender differences among Chinese patients with neuromyelitis optica spectrum disorders. Mult Scler Relat Disord 17:5-8. https://doi.org/10.1016/j.msard.2017.06.008

86. Tarhan B, Rempe T, Rahman S, Rodriguez E, Sladky J, Tuna IS, Rees J (2022) A Comparison of Pediatric- and Adult-Onset Aquaporin-4 Immunoglobulin G-Positive Neuromyelitis Optica Spectrum Disorder: A Review of Clinical and Radiographic Characteristics. J Child Neurol 37(8-9):727-37. https://doi.org/10.1177/08830738221103085

87. Tian DC, Li Z, Yuan M, Zhang C, Gu H, Wang Y, Shi FD (2020) Incidence of neuromyelitis optica spectrum disorder (NMOSD) in China: A national population-based study. Lancet Reg Health West Pac 2:100021. https://doi.org/10.1016/j.lanwpc.2020.100021

88. van Pelt ED, Wong YY, Ketelslegers IA, Hamann D, Hintzen RQ (2016) Neuromyelitis optica spectrum disorders: comparison of clinical and magnetic resonance imaging characteristics of AQP4-IgG versus MOG-IgG seropositive cases in the Netherlands. Eur J Neurol 23(3):580-7. https://doi.org/10.1111/ene.12898

89. Wu Y, Yang M, Gao P, Wang Z, Wu J, Wang J, Xu Q, Zhou H, Wu T, Wu W, Wei S, Hu YH (2022) Incidence of neuromyelitis optica spectrum disorders in China: a large cohort study using claim data. BMJ Open 12(1):e048942. https://doi.org/10.1136/bmjopen-2021-048942

90. Yin J, Long Y, Shan F, Fan Y, Wu L, Zhong R, Gao C, Chen X, Gao Q, Yang N (2015) Clinical manifestations of neuromyelitis optica in male and female patients. Neurol Res 37(11):967-73. https://doi.org/10.1179/1743132815Y.0000000081

91. Banwell B, Tenembaum S, Lennon VA, Ursell E, Kennedy J, Bar-Or A, Weinshenker BG, Lucchinetti CF, Pittock SJ (2008) Neuromyelitis optica-IgG in childhood inflammatory demyelinating CNS disorders. Neurology 70(5):344-52. https://doi.org/10.1212/01.wnl.0000284600.80782.d5

92. Camera V, Messina S, Elhadd KT, Sanpera-Iglesias J, Mariano R, Hacohen Y, Dobson R, Meletti S, Wassmer E, Lim MJ, Huda S, Hemingway C, Leite MI, Ramdas S, Palace J (2022) Early predictors of disability of paediatric-onset AQP4-IgG-seropositive neuromyelitis optica spectrum disorders. J Neurol Neurosurg Psychiatry 93(1):101-11. https://doi.org/10.1136/jnnp-2021-327206

93. Collongues N, Marignier R, Zephir H, Papeix C, Fontaine B, Blanc F, Rodriguez D, Fleury M, Vukusic S, Pelletier J, Audoin B, Thouvenot E, Camu W, Barroso B, Ruet A, Brochet B, Vermersch P, Confavreux C, de Seze J (2010) Long-term follow-up of neuromyelitis optica with a pediatric onset. Neurology 75(12):1084-8. https://doi.org/10.1212/WNL.0b013e3181f39a66

94. Huppke P, Bluthner M, Bauer O, Stark W, Reinhardt K, Huppke B, Gartner J (2010) Neuromyelitis optica and NMO-IgG in European pediatric patients. Neurology 75(19):1740-4. https://doi.org/10.1212/WNL.0b013e3181fc2823

95. Lin WS, Wang HP, Chen HM, Lin JW, Lee WT (2020) Epidemiology of pediatric multiple sclerosis, neuromyelitis optica, and optic neuritis in Taiwan. J Neurol 267(4):925-32. https://doi.org/10.1007/s00415-019-09647-9

96. Lotze TE, Northrop JL, Hutton GJ, Ross B, Schiffman JS, Hunter JV (2008) Spectrum of pediatric neuromyelitis optica. Pediatrics 122(5):e1039-47. https://doi.org/10.1542/peds.2007-2758

97. Martins C, Moura J, Figueiroa S, Garrido C, Martins J, Samoes R, Guimaraes J, Melo C, Sousa R, Palavra F, Ferreira J, da Silva AM, Sa MJ, Santos E (2022) Pediatric neuromyelitis optica spectrum disorders in Portugal: A multicentre retrospective study. Mult Scler Relat Disord 59:103531. https://doi.org/10.1016/j.msard.2022.103531

98. McKeon A, Lennon VA, Lotze T, Tenenbaum S, Ness JM, Rensel M, Kuntz NL, Fryer JP, Homburger H, Hunter J, Weinshenker BG, Krecke K, Lucchinetti CF, Pittock SJ (2008) CNS aquaporin-4 autoimmunity in children. Neurology 71(2):93-100. https://doi.org/10.1212/01.wnl.0000314832.24682.c6

99. Paolilo RB, Hacohen Y, Yazbeck E, Armangue T, Bruijstens A, Lechner C, Apostolos-Pereira SL, Martynenko Y, Breu M, de Medeiros Rimkus C, Wassmer E, Baumann M, Papetti L, Capobianco M, Kornek B, Rostasy K, da Paz JA, Ciccarelli O, Lim M, Saiz A, Neuteboom R, Marignier R, Hemingway C, Sato DK, Deiva K (2020) Treatment and outcome of aquaporin-4 antibody-positive NMOSD: A multinational pediatric study. Neurol Neuroimmunol Neuroinflamm 7(5):e837. https://doi.org/10.1212/NXI.0000000000000837

100. Yamaguchi Y, Torisu H, Kira R, Ishizaki Y, Sakai Y, Sanefuji M, Ichiyama T, Oka A, Kishi T, Kimura S, Kubota M, Takanashi J, Takahashi Y, Tamai H, Natsume J, Hamano S, Hirabayashi S, Maegaki Y, Mizuguchi M, Minagawa K, Yoshikawa H, Kira J, Kusunoki S, Hara T (2016) A nationwide survey of pediatric acquired demyelinating syndromes in Japan. Neurology 87(19):2006-15. https://doi.org/10.1212/WNL.0000000000003318

101. Yoon HH, Park JY, Kim SY, Lee NM, Yi DY, Yun SW, Lim IS, Chae SA (2021) Epidemiology of Demyelinating Diseases in Korean Pediatric Patients. J Child Neurol 36(2):141-7. https://doi.org/10.1177/0883073820959543

102. Zhou Y, Zhong X, Shu Y, Cui C, Wang J, Wang Y, Li X, Chen Z, Peng L, Kermode A, Qiu W (2019) Clinical course, treatment responses and outcomes in Chinese paediatric neuromyelitis optica spectrum disorder. Mult Scler Relat Disord 28:213-20. https://doi.org/10.1016/j.msard.2018.12.038

103. Cai LJ, Zhang Q, Zhang Y, Chen HX, Shi ZY, Du Q, Zhou HY (2020) Clinical characteristics of very late-onset neuromyelitis optica spectrum disorder. Mult Scler Relat Disord 46:102515. https://doi.org/10.1016/j.msard.2020.102515

104. Carnero Contentti E, Daccach Marques V, Soto de Castillo I, Tkachuk V, Ariel B, Castillo MC, Cristiano E, Diegues Serva GB, Dos Santos AC, Finkelsteyn AM, Lopez PA, Patrucco L, Molina O, Pettinicchi JP, Toneguzzo V, Caride A, Rojas JI (2020) Clinical features and prognosis of late-onset neuromyelitis optica spectrum disorders in a Latin American cohort. J Neurol 267(5):1260-8. https://doi.org/10.1007/s00415-020-09699-2

105. Collongues N, Marignier R, Jacob A, Leite MI, Siva A, Paul F, Zephir H, Akman-Demir G, Elsone L, Jarius S, Papeix C, Mutch K, Saip S, Wildemann B, Kitley J, Karabudak R, Aktas O, Kuscu D, Altintas A, Palace J, Confavreux C, De Seze J (2014) Characterization of neuromyelitis optica and neuromyelitis optica spectrum disorder patients with a late onset. Mult Scler 20(8):1086-94. https://doi.org/10.1177/1352458513515085

106. Delgado-Garcia G, Antonio-Luna E, Lopez-Mena D, Rivas-Alonso V, Flores-Rivera J, Corona-Vazquez T (2020) AQP4-IgG-positive neuromyelitis optica spectrum disorder with late onset in Mexico. Mult Scler Relat Disord 43:102221. https://doi.org/10.1016/j.msard.2020.102221

107. Lavandier N, Bonnan M, Carra-Dalliere C, Charif M, Labauge P, Camdessanche JP, Edan G, Naudin A, Brassat D, Ciron J, Clavelou P, Dulau C, Moroso A, Brochet B, Ouallet JC, on behalf Societe Francophone de la Sclerose en P (2019) First clinical inflammatory demyelinating events of the central nervous system in a population aged over 70 years: A multicentre study. Mult Scler Relat Disord 28:309-12. https://doi.org/10.1016/j.msard.2018.12.016

108. Mao Z, Yin J, Zhong X, Zhao Z, Qiu W, Lu Z, Hu X (2015) Late-onset neuromyelitis optica spectrum disorder in AQP4-seropositivepatients in a Chinese population. BMC Neurol 15:160. https://doi.org/10.1186/s12883-015-0417-y

109. Nakahara K, Nakane S, Nagaishi A, Narita T, Matsuo H, Ando Y (2021) Very late onset neuromyelitis optica spectrum disorders. Eur J Neurol 28(8):2574-81. https://doi.org/10.1111/ene.14901

110. Seok JM, Cho HJ, Ahn SW, Cho EB, Park MS, Joo IS, Shin HY, Kim SY, Kim BJ, Kim JK, Cho JY, Huh SY, Kwon O, Lee KH, Kim BJ, Min JH (2017) Clinical characteristics of late-onset neuromyelitis optica spectrum disorder: A multicenter retrospective study in Korea. Mult Scler 23(13):1748-56. https://doi.org/10.1177/1352458516685416

111. Sepulveda M, Delgado-Garcia G, Blanco Y, Sola-Valls N, Martinez-Lapiscina EH, Armangue T, Montejo C, Pulido-Valdeolivas I, Martinez-Hernandez E, Arino H, Escudero D, Ruiz-Garcia R, Llufriu S, Dalmau J, Graus F, Saiz A (2019) Late-onset neuromyelitis optica spectrum disorder: The importance of autoantibody serostatus. Neurol Neuroimmunol Neuroinflamm 6(6):e607. https://doi.org/10.1212/NXI.0000000000000607

112. Zhang LJ, Yang LN, Li T, Wang J, Qi Y, Zhang DQ, Yang CS, Yang L (2017) Distinctive characteristics of early-onset and late-onset neuromyelitis optica spectrum disorders. Int J Neurosci 127(4):334-8. https://doi.org/10.1080/00207454.2016.1254630

113. Bukhari W, Clarke L, O'Gorman C, Khalilidehkordi E, Arnett S, Prain KM, Woodhall M, Silvestrini R, Bundell CS, Ramanathan S, Abernethy D, Bhuta S, Blum S, Boggild M, Boundy K, Brew BJ, Brownlee W, Butzkueven H, Carroll WM, Chen C, Coulthard A, Dale RC, Das C, Dear K, Fabis-Pedrini MJ, Fulcher D, Gillis D, Hawke S, Heard R, Henderson APD, Heshmat S, Hodgkinson S, Jimenez-Sanchez S, Kilpatrick TJ, King J, Kneebone C, Kornberg AJ, Lechner-Scott J, Lin MW, Lynch C, Macdonnell RAL, Mason DF, McCombe PA, Pereira J, Pollard JD, Reddel SW, Shaw C, Spies J, Stankovich J, Sutton I, Vucic S, Walsh M, Wong RC, Yiu EM, Barnett MH, Kermode AG, Marriott MP, Parratt J, Slee M, Taylor BV, Willoughby E, Wilson RJ, Brilot F, Vincent A, Waters P, Broadley SA (2020) The clinical profile of NMOSD in Australia and New Zealand. J Neurol 267(5):1431-43. https://doi.org/10.1007/s00415-020-09716-4

114. Jarius S, Ruprecht K, Wildemann B, Kuempfel T, Ringelstein M, Geis C, Kleiter I, Kleinschnitz C, Berthele A, Brettschneider J, Hellwig K, Hemmer B, Linker RA, Lauda F, Mayer CA, Tumani H, Melms A, Trebst C, Stangel M, Marziniak M, Hoffmann F, Schippling S, Faiss JH, Neuhaus O, Ettrich B, Zentner C, Guthke K, Hofstadt-van Oy U, Reuss R, Pellkofer H, Ziemann U, Kern P, Wandinger KP, Bergh FT, Boettcher T, Langel S, Liebetrau M, Rommer PS, Niehaus S, Munch C, Winkelmann A, Zettl UU, Metz I, Veauthier C, Sieb JP, Wilke C, Hartung HP, Aktas O, Paul F (2012) Contrasting disease patterns in seropositive and seronegative neuromyelitis optica: A multicentre study of 175 patients. J Neuroinflammation 9:14. https://doi.org/10.1186/1742-2094-9-14

115. Nagaishi A, Takagi M, Umemura A, Tanaka M, Kitagawa Y, Matsui M, Nishizawa M, Sakimura K, Tanaka K (2011) Clinical features of neuromyelitis optica in a large Japanese cohort: comparison between phenotypes. J Neurol Neurosurg Psychiatry 82(12):1360-4. https://doi.org/10.1136/jnnp-2011-300403

116. Alvarenga MP, Schimidt S, Alvarenga RP (2017) Epidemiology of neuromyelitis optica in Latin America. Mult Scler J Exp Transl Clin 3(3):2055217317730098. https://doi.org/10.1177/2055217317730098

117. Asgari N, Lillevang ST, Skejoe HPB, Kyvik KO (2019) Epidemiology of neuromyelitis optica spectrum disorder in Denmark (1998-2008, 2007-2014). Brain Behav 9(7):e01338. https://doi.org/10.1002/brb3.1338

118. Ashtari F, Safaei A, Shaygannejad V, Najafi MA, Vesal S (2017) Neuromyelitis optica spectrum disease characteristics in Isfahan, Iran: A cross-sectional study. J Res Med Sci 22:41. https://doi.org/10.4103/1735-1995.202142

119. Badihian S, Manouchehri N, Mirmosayyeb O, Ashtari F, Shaygannejad V (2018) Neuromyelitis optica spectrum disorder and menstruation. Rev Neurol (Paris) 174(10):716-21. https://doi.org/10.1016/j.neurol.2018.01.373

120. Bergamaschi R, Ghezzi A (2004) Devic's neuromyelitis optica: clinical features and prognostic factors. Neurol Sci 25 Suppl 4:S364-7. https://doi.org/10.1007/s10072-004-0342-0

121. Bukhari W, Khalilidehkordi E, Mason DF, Barnett MH, Taylor BV, Fabis-Pedrini M, Kermode AG, Subramanian S, Waters P, Broadley SA, Australian, New Zealand NMOC (2022) NMOSD and MS prevalence in the Indigenous populations of Australia and New Zealand. J Neurol 269(2):836-45. https://doi.org/10.1007/s00415-021-10665-9

122. Cabre P, Gonzalez-Quevedo A, Lannuzel A, Bonnan M, Merle H, Olindo S, Chausson N, Lara-Rodriguez R, Smadja D, Cabrera-Gomez J (2009) [Descriptive epidemiology of neuromyelitis optica in the Caribbean basin]. Rev Neurol (Paris) 165(8-9):676-83. https://doi.org/10.1016/j.neurol.2009.02.012

123. Carnero Contentti E, Daccach Marques V, Soto de Castillo I, Tkachuk V, Lopez PA, Rojas JI (2020) Age at onset correlate with disability in Latin American aquaporin-4-IgG-positive NMOSD patients. Mult Scler Relat Disord 44:102258. https://doi.org/10.1016/j.msard.2020.102258

124. Cheng Q, Miao L, Zhang J, Guan YT, Liu ZG, Wang X, Sun XJ, Zhao ZX, Song YJ, Ding XY, Guo ZI, Cheng XJ, Chen SD, Jiang GX, Fredrikson S (2008) Clinical features of patients with multiple sclerosis from a survey in Shanghai, China. Mult Scler 14(5):671-8. https://doi.org/10.1177/1352458507087844

125. Choy BNK, Ng ALK, Lai JSM (2018) Clinical characteristics of optic neuritis in Hong Kong population: 10-year review. Int Ophthalmol 38(2):557-64. https://doi.org/10.1007/s10792-017-0491-9

126. Collongues N, Marignier R, Zephir H, Blanc F, Vukusic S, Outteryck O, Fleury M, Ruet A, Borgel F, Thouvenot E, Moreau T, Defer G, Derache N, Pelletier J, Audoin B, Debouverie M, Labauge P, Gout O, Camu W, Brassat D, Brochet B, Vermersch P, Confavreux C, de Seze J (2011) High-risk syndrome for neuromyelitis optica: a descriptive and comparative study. Mult Scler 17(6):720-4. https://doi.org/10.1177/1352458510396923

127. Cristiano E, Patrucco L, Miguez J, Giunta D, Peroni J, Rojas JI (2016) Increasing incidence of multiple sclerosis among women in Buenos Aires: a 22 year health maintenance organization based study. Neurol Sci 37(10):1621-6. https://doi.org/10.1007/s10072-016-2637-3

128. Dale GH, Svendsen KB, Gjelstrup MC, Christensen T, Houen G, Nielsen E, Bek T, Petersen T (2018) Incidence of neuromyelitis optica spectrum disorder in the Central Denmark Region. Acta Neurol Scand 137(6):582-8. https://doi.org/10.1111/ane.12903

129. Domingos J, Isidoro L, Figueiredo R, Brum M, Capela C, Barros P, Santos E, Macario Mdo C, Pinto Marques J, Pedrosa R, Vale J, Sa MJ (2015) Neuromyelitis optica in Portugal (NEMIPORT) - A multicentre study. Clin Neurol Neurosurg 134:79-84. https://doi.org/10.1016/j.clineuro.2015.04.001

130. Eskandarieh S, Nedjat S, Abdollahpour I, Moghadasi AN, Azimi AR, Sahraian MA (2017) Comparing epidemiology and baseline characteristic of multiple sclerosis and neuromyelitis optica: A case-control study. Mult Scler Relat Disord 12:39-43. https://doi.org/10.1016/j.msard.2017.01.004

131. Eskandarieh S, Nedjat S, Abdollahpour I, Azimi AR, Moghadasi AN, Asgari N, Sahraian MA (2018) Environmental risk factors in neuromyelitis optica spectrum disorder: a case-control study. Acta Neurol Belg 118(2):277-87. https://doi.org/10.1007/s13760-018-0900-5

132. Etemadifar M, Dashti M, Vosoughi R, Abtahi SH, Ramagopalan SV, Nasr Z (2014) An epidemiological study of neuromyelitis optica in Isfahan. Mult Scler 20(14):1920-2. https://doi.org/10.1177/1352458514537699

133. Etemadifar M, Mehrbod N, Dehghani L, Golabbakhsh A, Fereidan-Esfahani M, Akbari M, Nasr Z (2014) Prevalence of Lhermitte's sign in multiple sclerosis versus neuromyelitis optica. Iran J Neurol 13(1):50-1. PMC3968359

134. Fragoso YD, Sousa NAC, Saad T, Alves-Leon SV, Pimentel MLV, Goncalves MVM, Stella CV, Diniz DS, Santos GC, Gomes S, Adoni T, Anacleto A, Claudino R, Malfetano FR, Winckler TCD, Damasceno A, Eboni ACB, Farinhas JGD, Mota RSS (2019) Clinical Characteristics of Patients With Neuromyelitis Optica Spectrum Disorders With Early Onset. J Child Neurol 34(9):487-90. https://doi.org/10.1177/0883073819842421

135. Gold SM, Willing A, Leypoldt F, Paul F, Friese MA (2019) Sex differences in autoimmune disorders of the central nervous system. Semin Immunopathol 41(2):177-88. https://doi.org/10.1007/s00281-018-0723-8

136. Hoftberger R, Sepulveda M, Armangue T, Blanco Y, Rostasy K, Calvo AC, Olascoaga J, Ramio-Torrenta L, Reindl M, Benito-Leon J, Casanova B, Arrambide G, Sabater L, Graus F, Dalmau J, Saiz A (2015) Antibodies to MOG and AQP4 in adults with neuromyelitis optica and suspected limited forms of the disease. Mult Scler 21(7):866-74. https://doi.org/10.1177/1352458514555785

137. Houzen H, Niino M, Hirotani M, Fukazawa T, Kikuchi S, Tanaka K, Sasaki H (2012) Increased prevalence, incidence, and female predominance of multiple sclerosis in northern Japan. J Neurol Sci 323(1-2):117-22. https://doi.org/10.1016/j.jns.2012.08.032

138. Houzen H, Kondo K, Horiuchi K, Niino M (2018) Consistent increase in the prevalence and female ratio of multiple sclerosis over 15 years in northern Japan. Eur J Neurol 25(2):334-9. https://doi.org/10.1111/ene.13506

139. Jacob A, Panicker J, Lythgoe D, Elsone L, Mutch K, Wilson M, Das K, Boggild M (2013) The epidemiology of neuromyelitis optica amongst adults in the Merseyside county of United Kingdom. J Neurol 260(8):2134-7. https://doi.org/10.1007/s00415-013-6926-y

140. Jarius S, Frederikson J, Waters P, Paul F, Akman-Demir G, Marignier R, Franciotta D, Ruprecht K, Kuenz B, Rommer P, Kristoferitsch W, Wildemann B, Vincent A (2010) Frequency and prognostic impact of antibodies to aquaporin-4 in patients with optic neuritis. J Neurol Sci 298(1-2):158-62. https://doi.org/10.1016/j.jns.2010.07.011

141. Jarius S, Ruprecht K, Kleiter I, Borisow N, Asgari N, Pitarokoili K, Pache F, Stich O, Beume LA, Hummert MW, Trebst C, Ringelstein M, Aktas O, Winkelmann A, Buttmann M, Schwarz A, Zimmermann H, Brandt AU, Franciotta D, Capobianco M, Kuchling J, Haas J, Korporal-Kuhnke M, Lillevang ST, Fechner K, Schanda K, Paul F, Wildemann B, Reindl M, in cooperation with the Neuromyelitis Optica Study G (2016) MOG-IgG in NMO and related disorders: a multicenter study of 50 patients. Part 1: Frequency, syndrome specificity, influence of disease activity, long-term course, association with AQP4-IgG, and origin. J Neuroinflammation 13(1):279. https://doi.org/10.1186/s12974-016-0717-1

142. Kim SH, Mealy MA, Levy M, Schmidt F, Ruprecht K, Paul F, Ringelstein M, Aktas O, Hartung HP, Asgari N, Tsz-Ching JL, Siritho S, Prayoonwiwat N, Shin HJ, Hyun JW, Han M, Leite MI, Palace J, Kim HJ (2018) Racial differences in neuromyelitis optica spectrum disorder. Neurology 91(22):e2089-e99. https://doi.org/10.1212/WNL.0000000000006574

143. Kim JE, Park SH, Han K, Kim HJ, Shin DW, Kim SM (2020) Prevalence and incidence of neuromyelitis optica spectrum disorder and multiple sclerosis in Korea. Mult Scler 26(14):1837-44. https://doi.org/10.1177/1352458519888609

144. Krumbholz M, Hofstadt-van Oy U, Angstwurm K, Kleiter I, Jarius S, Paul F, Aktas O, Buchholz G, Kern P, Straube A, Kumpfel T (2015) Very late-onset neuromyelitis optica spectrum disorder beyond the age of 75. J Neurol 262(5):1379-84. https://doi.org/10.1007/s00415-015-7766-8

145. Lee JD, Guimond C, Yee IM, Vilarino-Guell C, Wu ZY, Traboulsee AL, Sadovnick AD (2015) Incidence of Multiple Sclerosis and Related Disorders in Asian Populations of British Columbia. Can J Neurol Sci 42(4):235-41. https://doi.org/10.1017/cjn.2015.36

146. Lee HL, Kim JY, Seok JM, Hong YH, Lim NG, Shin HY, Kim BJ, Hwang SY, Min JH, Kim BJ (2020) Prevalence and Incidence of Neuromyelitis Optica Spectrum Disorder in Korea: Population Based Study. J Korean Med Sci 35(17):e115. https://doi.org/10.3346/jkms.2020.35.e115

147. Li Y, Zhang J, Zhou Y, Xie H, Duan R, Jing L, Yao Y, Teng J, Jia Y (2021) Analysis of Predictive Risk Factors in Aquaporin-4-IgG Positive Highly Active Neuromyelitis Optica Spectrum Disorders. Front Neurol 12:731835. https://doi.org/10.3389/fneur.2021.731835

148. Marignier R, Cobo Calvo A, Vukusic S (2017) Neuromyelitis optica and neuromyelitis optica spectrum disorders. Curr Opin Neurol 30(3):208-15. https://doi.org/10.1097/WCO.0000000000000455

149. Mealy MA, Wingerchuk DM, Greenberg BM, Levy M (2012) Epidemiology of neuromyelitis optica in the United States: a multicenter analysis. Arch Neurol 69(9):1176-80. https://doi.org/10.1001/archneurol.2012.314

150. O'Connell K, Hamilton-Shield A, Woodhall M, Messina S, Mariano R, Waters P, Ramdas S, Leite MI, Palace J (2020) Prevalence and incidence of neuromyelitis optica spectrum disorder, aquaporin-4 antibody-positive NMOSD and MOG antibody-positive disease in Oxfordshire, UK. J Neurol Neurosurg Psychiatry 91(10):1126-8. https://doi.org/10.1136/jnnp-2020-323158

151. Ortiz Salas PA, Gaviria Carrillo M, Cortes Bernal GA, Moreno Medina K, Roa LF, Rodriguez Quintana JH (2022) Neuromyelitis optica spectrum disorder: do patients positive and negative for anti-aquaporin-4 antibodies present distinct entities? A Colombian perspective. Neurologia (Engl Ed) 38(7):504-10. https://doi.org/10.1016/j.nrleng.2020.08.022

152. Papp V, Illes Z, Magyari M, Koch-Henriksen N, Kant M, Pfleger CC, Roemer SF, Jensen MB, Petersen AE, Nielsen HH, Rosendahl L, Mezei Z, Christensen T, Svendsen K, Hyldgaard Jensen PE, Lydolph MC, Heegaard N, Frederiksen JL, Sellebjerg F, Stenager E, Petersen T (2018) Nationwide prevalence and incidence study of neuromyelitis optica spectrum disorder in Denmark. Neurology 91(24):e2265-e75. https://doi.org/10.1212/WNL.0000000000006645

153. Park TJ, Kim JH, Kim HJ, Bae JS, Cheong HS, Park BL, Shin HD (2014) Lack of association between AQP4 polymorphisms and risk of inflammatory demyelinating disease in a Korean population. Gene 536(2):302-7. https://doi.org/10.1016/j.gene.2013.12.007

154. Pittock SJ, Lennon VA, Bakshi N, Shen L, McKeon A, Quach H, Briggs FB, Bernstein AL, Schaefer CA, Barcellos LF (2014) Seroprevalence of aquaporin-4-IgG in a northern California population representative cohort of multiple sclerosis. JAMA Neurol 71(11):1433-6. https://doi.org/10.1001/jamaneurol.2014.1581

155. Rezaeimanesh N, Sahraian MA, Moghadasi AN, Eskandarieh S (2020) Epidemiology of neuromyelitis optica spectrum disorder in Tehran, Iran: the prevalence, baseline characteristics, and clinical aspects. Neurol Sci 41(9):2647-8. https://doi.org/10.1007/s10072-020-04393-7

156. Sahraian MA, Moinfar Z, Khorramnia S, Ebrahim MM (2010) Relapsing neuromyelitis optica: demographic and clinical features in Iranian patients. Eur J Neurol 17(6):794-9. https://doi.org/10.1111/j.1468-1331.2009.02928.x

157. Saiz A, Zuliani L, Blanco Y, Tavolato B, Giometto B, Graus F, Spanish-Italian NMOSG (2007) Revised diagnostic criteria for neuromyelitis optica (NMO). Application in a series of suspected patients. J Neurol 254(9):1233-7. https://doi.org/10.1007/s00415-007-0509-8

158. Sepulveda M, Aldea M, Escudero D, Llufriu S, Arrambide G, Otero-Romero S, Sastre-Garriga J, Romero-Pinel L, Martinez-Yelamos S, Sola-Valls N, Armangue T, Sotoca J, Escartin A, Robles-Cedeno R, Ramio-Torrenta L, Presas-Rodriguez S, Ramo-Tello C, Munteis E, Pelayo R, Gubieras L, Brieva L, Ortiz N, Hervas M, Mane-Martinez MA, Cano A, Vela E, Tintore M, Blanco Y, Montalban X, Graus F, Saiz A (2018) Epidemiology of NMOSD in Catalonia: Influence of the new 2015 criteria in incidence and prevalence estimates. Mult Scler 24(14):1843-51. https://doi.org/10.1177/1352458517735191

159. Simaniv TO, Kochergin IA, Zakharova MN, Korobko DS, Zaslavskii LG, Zelenova OV, Abramov SI (2021) [Clinical and epidemiological aspects of neuromyelitis optic spectrum diseases in the russian population]. Zh Nevrol Psikhiatr Im S S Korsakova 121(7):96-103. https://doi.org/10.17116/jnevro202112107196

160. Vanikieti K, Poonyathalang A, Jindahra P, Bouzika P, Rizzo JF, 3rd, Cestari DM (2017) Clinical characteristics and long-term visual outcome of optic neuritis in neuromyelitis optica spectrum disorder: A comparison between Thai and American-Caucasian cohorts. Mult Scler Relat Disord 17:87-91. https://doi.org/10.1016/j.msard.2017.07.013

161. Wang H, Dai Y, Qiu W, Zhong X, Wu A, Wang Y, Lu Z, Bao J, Hu X (2011) HLA-DPB1 0501 is associated with susceptibility to anti-aquaporin-4 antibodies positive neuromyelitis optica in southern Han Chinese. J Neuroimmunol 233(1-2):181-4. https://doi.org/10.1016/j.jneuroim.2010.11.004

162. Mumford CJ, Fraser MB, Wood NW, Compston DA (1992) Multiple sclerosis in the Cambridge health district of east Anglia. J Neurol Neurosurg Psychiatry 55(10):877-82. 1015180

163. Nakamura M, Ogawa R, Fujimori J, Uzawa A, Sato Y, Nagashima K, Kuriyama N, Kuwabara S, Nakashima I (2023) Epidemiological and clinical characteristics of myelin oligodendrocyte glycoprotein antibody-associated disease in a nationwide survey. Mult Scler 29(4-5):530-9. https://doi.org/10.1177/13524585231156736

164. Trojano M, Lucchese G, Graziano G, Taylor BV, Simpson S, Jr., Lepore V, Grand'maison F, Duquette P, Izquierdo G, Grammond P, Amato MP, Bergamaschi R, Giuliani G, Boz C, Hupperts R, Van Pesch V, Lechner-Scott J, Cristiano E, Fiol M, Oreja-Guevara C, Saladino ML, Verheul F, Slee M, Paolicelli D, Tortorella C, D'Onghia M, Iaffaldano P, Direnzo V, Butzkueven H, Group MSS, the New Zealand MSPSG (2012) Geographical variations in sex ratio trends over time in multiple sclerosis. PLoS One 7(10):e48078. https://doi.org/10.1371/journal.pone.0048078

165. Silcocks PB, Jenner DA, Reza R (2001) Life expectancy as a summary of mortality in a population: statistical considerations and suitability for use by health authorities. J Epidemiol Community Health 55(1):38-43. https://doi.org/10.1136/jech.55.1.38

166. Rider V, Abdou NI, Kimler BF, Lu N, Brown S, Fridley BL (2018) Gender Bias in Human Systemic Lupus Erythematosus: A Problem of Steroid Receptor Action? Front Immunol 9:611. https://doi.org/10.3389/fimmu.2018.00611

167. Kvarnstrom M, Ottosson V, Nordmark B, Wahren-Herlenius M (2015) Incident cases of primary Sjogren's syndrome during a 5-year period in Stockholm County: a descriptive study of the patients and their characteristics. Scand J Rheumatol 44(2):135-42. https://doi.org/10.3109/03009742.2014.931457

168. Pittock SJ, Lennon VA, de Seze J, Vermersch P, Homburger HA, Wingerchuk DM, Lucchinetti CF, Zephir H, Moder K, Weinshenker BG (2008) Neuromyelitis optica and non organ-specific autoimmunity. Arch Neurol 65(1):78-83. https://doi.org/10.1001/archneurol.2007.17

169. Desai MK, Brinton RD (2019) Autoimmune Disease in Women: Endocrine Transition and Risk Across the Lifespan. Front Endocrinol (Lausanne) 10:265. https://doi.org/10.3389/fendo.2019.00265

170. Romero-Pinel L, Bau L, Matas E, Leon I, Munoz-Vendrell A, Arroyo P, Masuet-Aumatell C, Martinez-Yelamos A, Martinez-Yelamos S (2022) The age at onset of relapsing-remitting multiple sclerosis has increased over the last five decades. Mult Scler Relat Disord 68:104103. https://doi.org/10.1016/j.msard.2022.104103

171. Hooge JP, Redekop WK (1992) Multiple sclerosis with very late onset. Neurology 42(10):1907-10. https://doi.org/10.1212/wnl.42.10.1907

172. Bermel RA, Rae-Grant AD, Fox RJ (2010) Diagnosing multiple sclerosis at a later age: more than just progressive myelopathy. Mult Scler 16(11):1335-40. https://doi.org/10.1177/1352458510377334

173. McGrogan A, Madle GC, Seaman HE, de Vries CS (2009) The epidemiology of Guillain-Barre syndrome worldwide. A systematic literature review. Neuroepidemiology 32(2):150-63. https://doi.org/10.1159/000184748

174. Childs B, Scriver CR (1986) Age at onset and causes of disease. Perspect Biol Med 29(3):437-60. https://doi.org/10.1353/pbm.1986.0056

175. Lee JY, Chitnis T (2016) Pediatric Multiple Sclerosis. Semin Neurol 36(2):148-53. https://doi.org/10.1055/s-0036-1579738

176. Li Y, Xie H, Zhang J, Zhou Y, Jing L, Yao Y, Duan R, Jia Y (2021) Clinical and Radiological Characteristics of Children and Adults With First-Attack Myelin Oligodendrocyte Glycoprotein Antibody Disease and Analysis of Risk Factors for Predicting the Severity at Disease Onset in Central China. Front Immunol 12:752557. https://doi.org/10.3389/fimmu.2021.752557
