# Supplementary material for: Sex ratio and age of onset in AQP4 antibody-associated NMOSD: a review and meta-analysis
Source: J Neurol. 2024 Jul 3;271(8):4794–812. doi: 10.1007/s00415-024-12452-8 (PMC11319503; doi:10.1007/s00415-024-12452-8)
Supplement: Supplementary file 2 — (PDF 1146 kb) [file 415_2024_12452_MOESM2_ESM.pdf]

Supplementary Figure 1

Study [Ref]	Sex Ratio	[95%CI]	Fem	Mal	Weight
Bizzoco et al., 2009 [36]	12.00	[1.81, 79.39]	12	1	0.20%
Collongues, 2010 [42]	3.03	[2.20, 4.18]	94	31	2.60%
Asgari et al., 2011 [32]	2.82	[1.64, 4.83]	31	11	1.50%
Cossburn et al., 2012 [32]	6.00	[1.63, 22.05]	12	2	0.40%
Aboul-Enein et al., 2013 [28]	6.89	[3.72, 12.77]	62	9	1.30%
Jiao et al., 2013 [58]	5.91	[4.03, 8.68]	136	23	2.20%
Kitley et al., 2014 [64]	9.00	[2.40, 33.78]	18	2	0.40%
Flanagan et al., 2016a [50]	5.00	[0.81, 30.99]	5	1	0.20%
Kleiter et al., 2016 [65]	4.61	[3.36, 6.31]	152	33	2.60%
Sepulveda et al., 2016 [80]	6.54	[4.49, 9.53]	157	24	2.20%
van Pelt et al., 2016 [88]	5.83	[2.75, 12.36]	35	6	0.90%
Bukhari et al., 2017 [37]	6.00	[4.02, 8.97]	126	21	2.10%
Drulovic et al., 2019 [46]	5.73	[3.29, 9.95]	63	11	1.50%
Jonsson et al., 2019 [59]	2.83	[1.97, 4.08]	68	24	2.30%
Papp et al., 2020 [73]	7.11	[4.64, 10.87]	135	19	2.00%
Stratos et al., 2020 [84]	7.10	[3.95, 12.76]	71	10	1.40%
Papp et al., 2021 [74]	7.75	[3.06, 19.63]	31	4	0.70%
Santos et al., 2021 [3]	7.56	[3.99, 13.75]	68	9	1.30%
Tarhan et al., 2022 [86]	5.14	[2.58, 10.26]	36	7	1.10%
**White/European/Caucasian**	**5.23**	**[4.37, 6.27]**	**1312**	**248**	**26.70%**
**τ^2=0.07, I^2=50%, X^2=34.17, df =17, p=0.008**					
Cabre et al., 2001 [38]	11.67	[1.73, 78.88]	17	0	0.20%
Rivera et al., 2008 [77]	2.40	[1.36, 4.22]	24	10	1.40%
Bichuetti et al., 2009 [35]	2.42	[1.44, 4.04]	29	12	1.60%
Cabrera-Gomez et al., 2009 [39]	7.29	[3.61, 14.70]	51	7	1.00%
Adoni et al., 2010 [29]	8.33	[2.84, 24.44]	25	3	0.50%
Papais-Alvarenga et al., 2015 [72]	5.46	[4.00, 7.44]	191	35	2.60%
Del Negro et al., 2017 [45]	7.50	[2.96, 18.99]	30	4	0.70%
Mealy et al., 2018 [66]	6.12	[4.83, 7.75]	367	60	3.10%
Rojas et al., 2020 [78]	3.69	[2.35, 5.79]	59	16	1.90%
Ibis et al., 2021 [56]	4.79	[3.63, 6.33]	206	43	2.80%
Paz et al., 2021 [75]	6.60	[2.89, 15.09]	33	5	0.80%
Alves et al., 2022 [31]	4.33	[2.37, 7.92]	39	9	1.30%
Fukuda et al., 2022 [51]	3.79	[2.51, 5.73]	72	19	2.00%
Gracia et al., 2022 [53]	5.64	[3.99, 7.98]	158	28	2.40%
Mireles-Ramirez et al., 2022 [67]	2.94	[1.91, 4.54]	50	17	2.00%
**South/Central American**	**4.56**	**[3.80, 5.47]**	**1351**	**268**	**24.40%**
**τ^2=0.06, I^2=54%, X^2=30.33, df =14, p=0.007**					
Altintas et al., 2015 [30]	5.42	[3.19, 9.19]	65	12	1.50%
Kashipazha et al., 2015 [61]	7.33	[3.43, 15.65]	44	6	0.90%
Daoudi & Bouzar, 2016 [44]	3.00	[0.85, 10.62]	6	2	0.40%
Flanagan et al., 2016b [50]	8.75	[3.44, 22.28]	35	4	0.70%
Eskandarieh et al., 2017 [47]	5.06	[3.25, 7.88]	86	17	1.90%
Holroyd et al., 2018 [54]	2.33	[0.83, 6.54]	7	3	0.60%
Salama et al., 2018 [79]	5.67	[1.96, 16.37]	17	3	0.50%
Shaygannejad et al., 2018 [81]	3.14	[1.60, 6.18]	22	7	1.10%
Bennis et al., 2019 [34]	3.57	[2.21, 5.77]	50	14	1.70%
Ceccarelli et al., 2020 [40]	3.67	[0.58, 23.09]	5	0	0.20%
Etemadifar et al., 2020 [49]	4.68	[3.08, 7.11]	89	19	2.00%
Rafiee et al., 2020 [76]	4.10	[2.95, 5.70]	123	30	2.50%
Shosha et al., 2020 [82]	4.76	[3.31, 6.85]	119	25	2.30%
Kishk et al., 2021 [63]	4.83	[2.86, 8.18]	58	12	1.60%
Mirmosayyeb et al., 2021 [68]	3.15	[2.46, 4.03]	167	53	3.10%
**Middle Eastern/African**	**4.19**	**[3.66, 4.79]**	**893**	**207**	**21.00%**
**τ^2=0.01, I^2=7%, X^2=15.13, df =14, p=0.37**					
Barhate et al., 2014 [33]	7.80	[3.40, 17.90]	39	5	0.80%
Pandit & Kundapur, 2014 [71]	1.20	[0.52, 2.79]	6	5	0.80%
Jagtap et al., 2015 [57]	4.20	[1.87, 9.44]	21	5	0.80%
Joshi et al., 2022 [60]	2.83	[1.69, 4.74]	34	12	1.60%
Netravathi et al., 2019 [70]	8.18	[4.66, 14.36]	90	11	1.40%
Singh et al., 2021 [83]	2.66	[1.91, 3.70]	77	29	2.50%
**South Asian/Indian Subcontinent**	**3.66**	**[2.20, 6.10]**	**267**	**67**	**8.00%**
**τ^2=0.30, I^2=77%, X^2=21.94, df =5, p=0.0005**					
Kim et al., 2012 [62]	10.78	[5.75, 20.17]	97	9	1.20%
Chan et al., 2013 [41]	6.83	[3.21, 14.54]	41	6	0.90%
Yin et al., 2015 [90]	5.75	[3.63, 9.09]	92	16	1.80%
Sun et al., 2017 [85]	5.47	[3.41, 8.77]	82	15	1.80%
Hor et al., 2018 [55]	9.67	[1.44, 64.73]	14	0	0.20%
Miyamoto et al., 2018 [69]	6.39	[5.47, 7.46]	901	141	3.70%
Espiritu et al., 2019 [48]	2.60	[1.17, 5.78]	13	5	0.90%
Gao et al., 2019 [52]	7.40	[4.11, 13.32]	74	10	1.30%
Tian et al., 2020 [87]	4.57	[4.40, 4.76]	9825	2148	4.20%
Wu et al., 2022 [89]	3.66	[3.28, 4.07]	1031	282	3.90%
**South East Asian**	**5.26**	**[4.38, 6.33]**	**12170**	**2632**	**19.90%**
**τ^2=0.04, I^2=82%, X^2=49.43, df =9, p<0.00001**					
**Overall (Random Effects Model)**	**4.70**	**[4.33, 5.11]**	**15993**	**3422**	**100.00%**
**τ^2=0.04, I^2=61%, X^2=161.72, df =63, p<0.00001**					

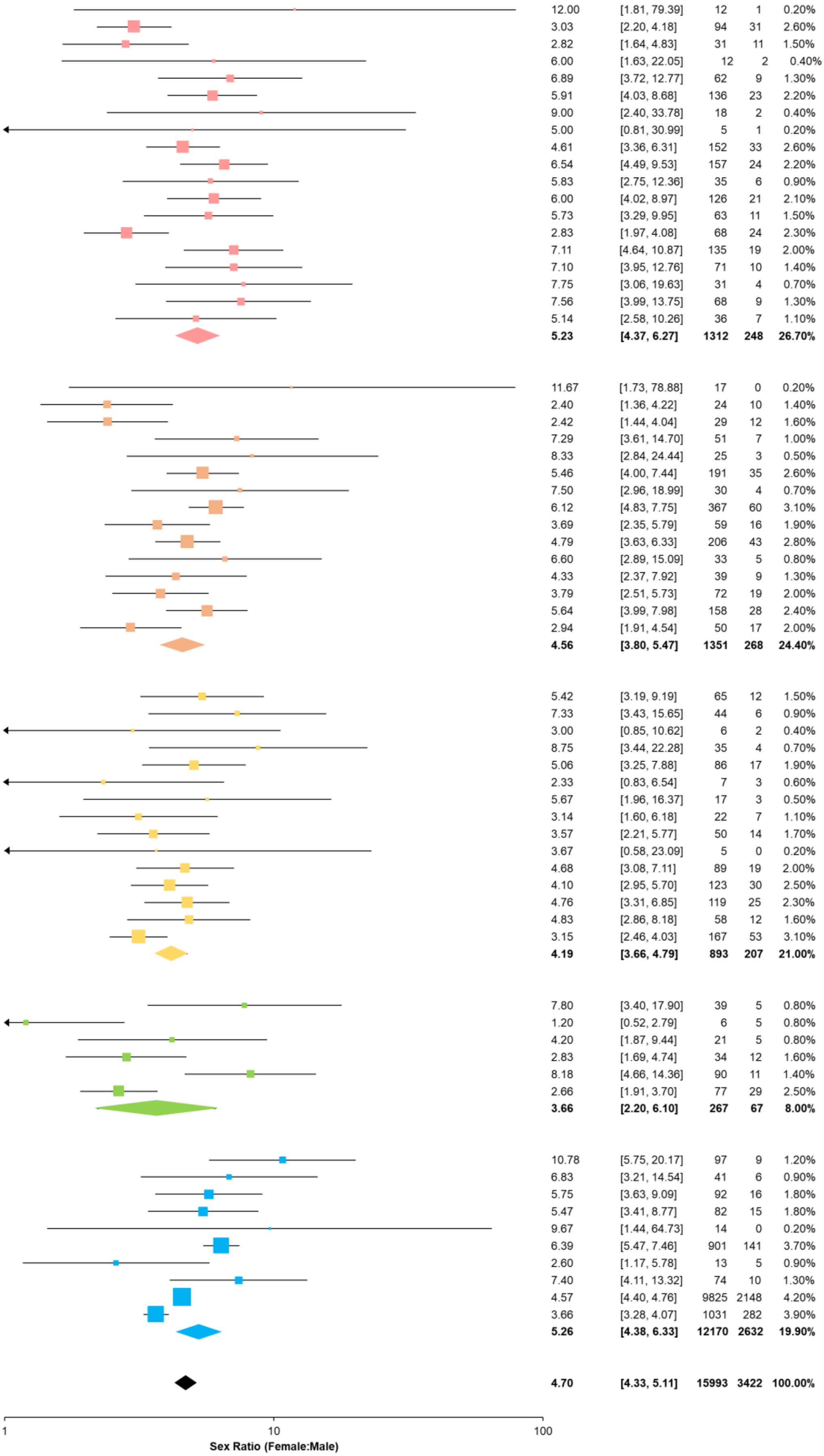

Key White/European/Caucasian; South/Central American; Middle Eastern/African; South Asian/Indian Subcontinent; East Asian

Forrest plot of sex ratio for all available studies subgrouped by geographical region. Inverse variance method with random effects model. CI = confidence interval; Fem = female; Mal = male.

Supplementary Figure 2

A

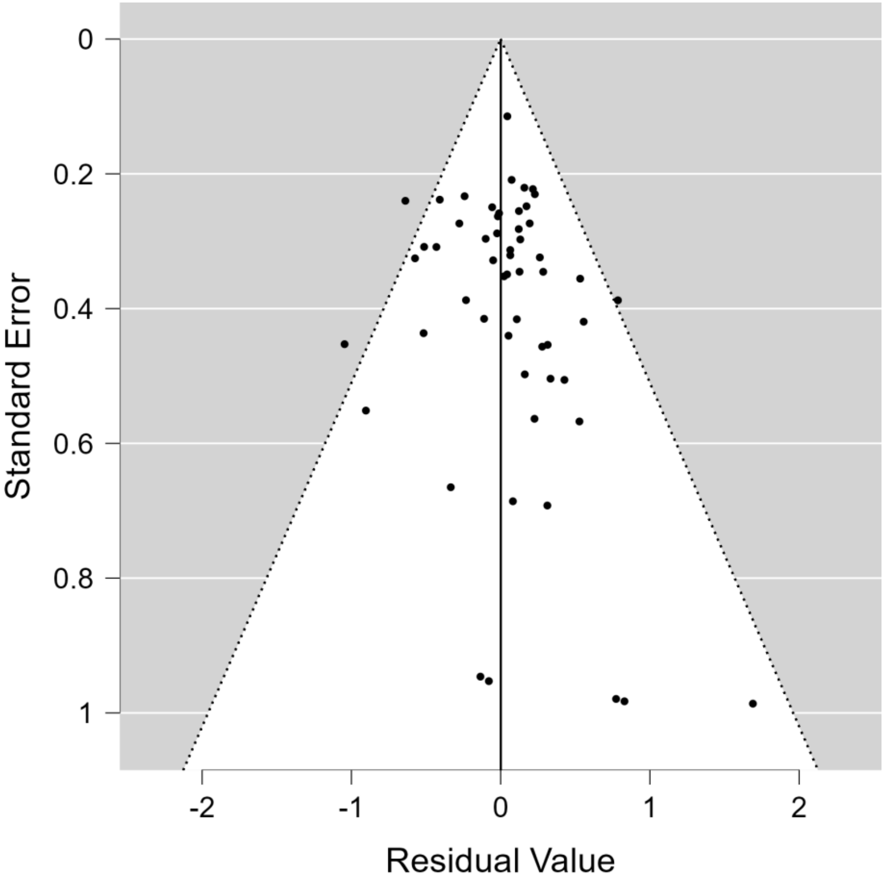

B

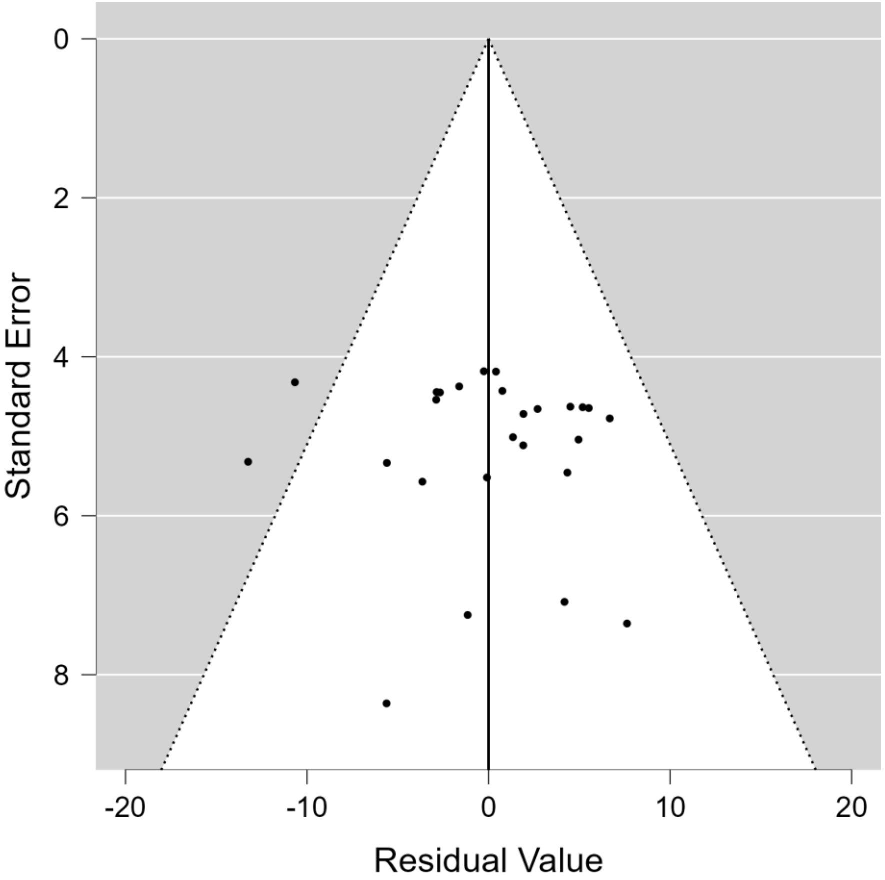

Funnel plots for sex ratio (A) and age of onset (B) meta-regression analyses. Age of onset analysis restricted to AQP4 antibody positive studies.

Supplementary Figure 3

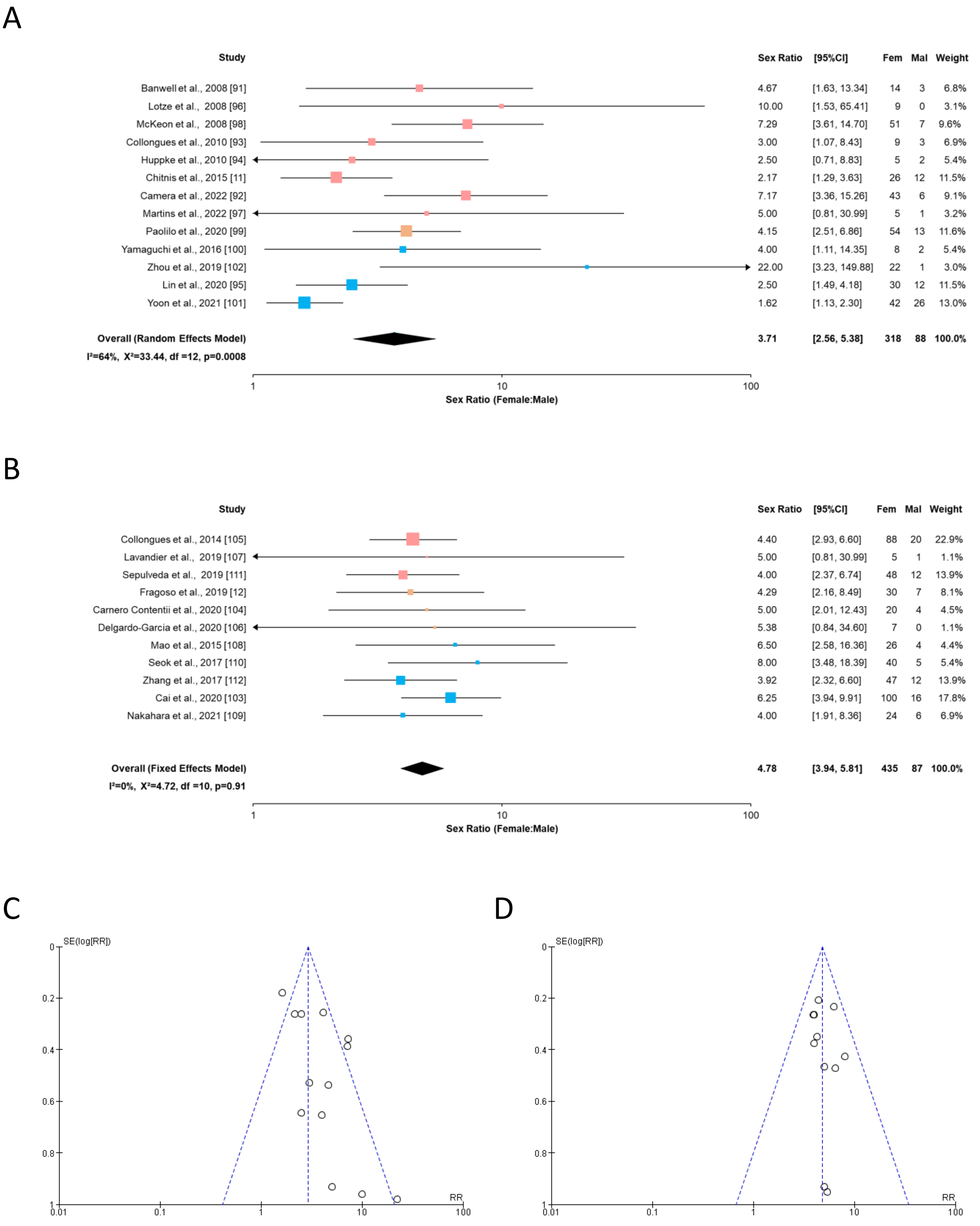

Forrest plots of sex ratio for paediatric (A) and late onset (B) studies and funnel plots of paediatric (C) and late onset (D) studies. SE = standard error; IV = inverse variance; CI = confidence interval; RR = risk ratio.

Supplementary Figure 4

A

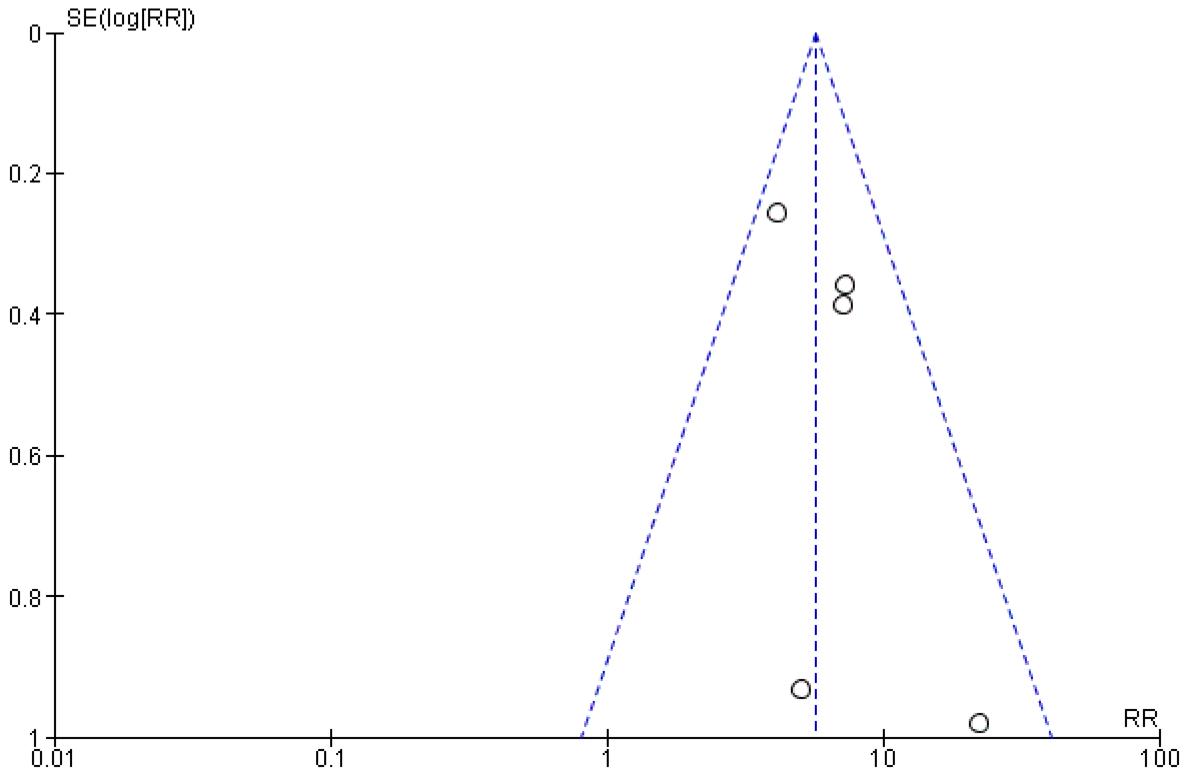

B

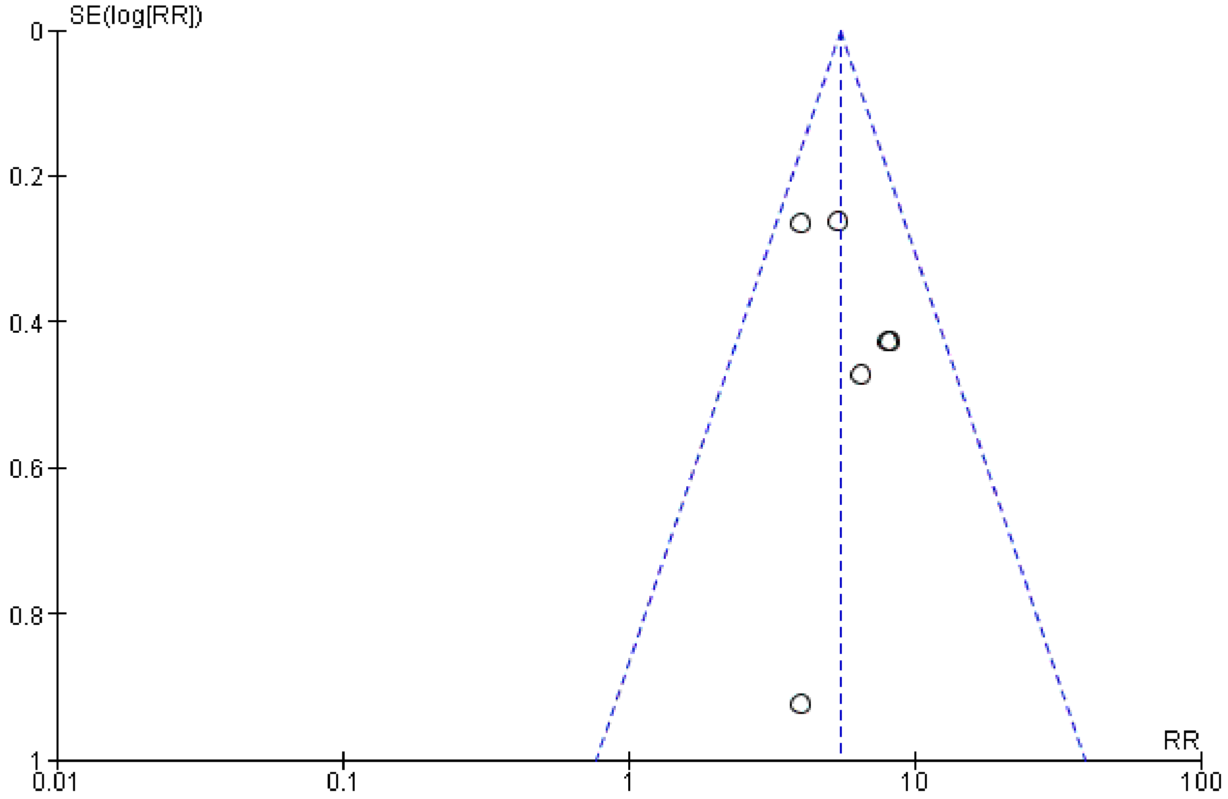

Funnel plots of sex ratio in paediatric (A) and late onset (B) studies restricted to AQP4 antibody positive studies. SE = standard error; RR = risk ratio.

Supplementary Figure 5

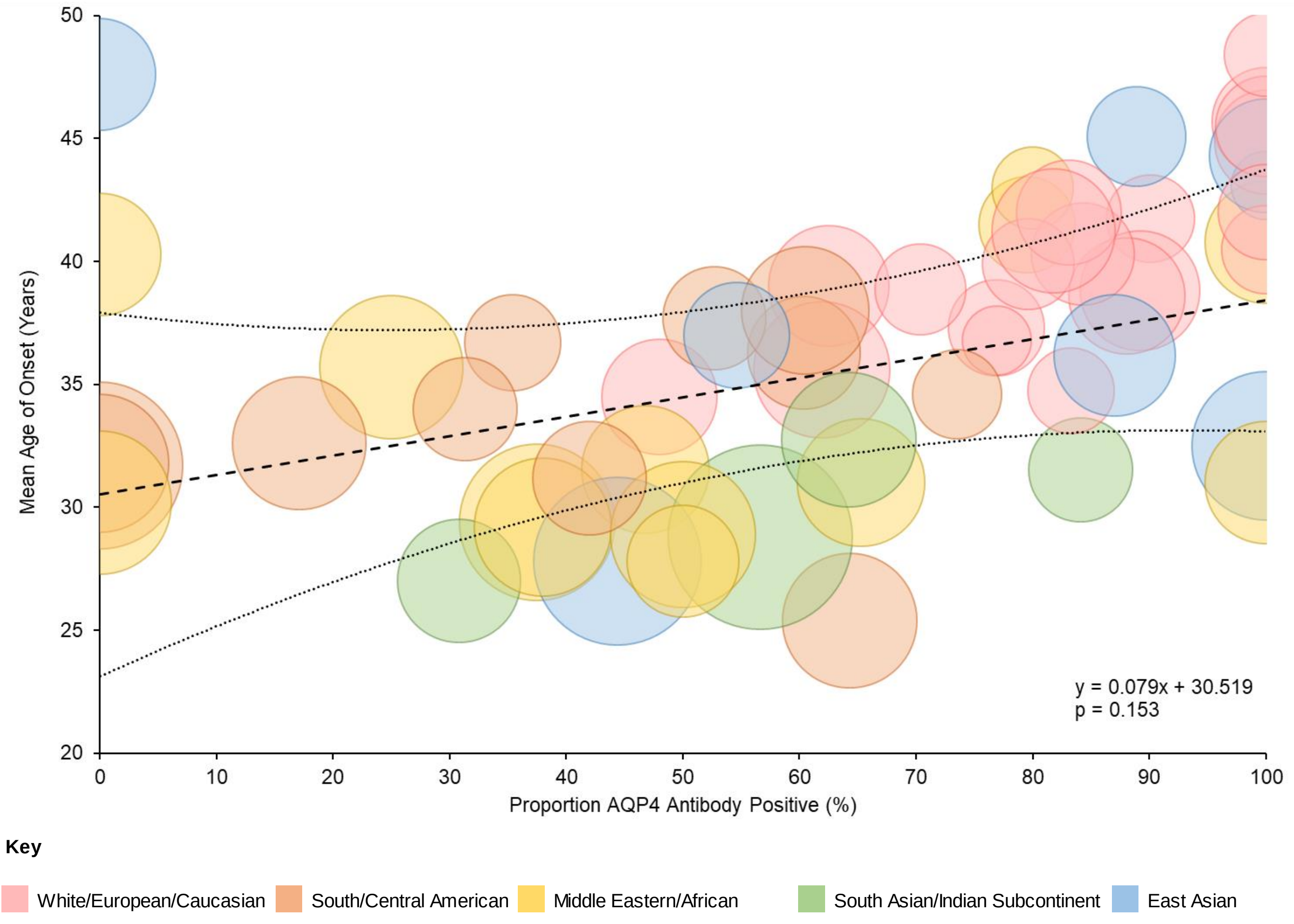

Bubble plot of mean age of onset against proportion of AQP4 antibody positive cases. Bubble size is proportional to the variance of the mean age of onset. Meta-regression model included geographical region. Dashed line indicates fitted regression from meta-regression analysis (indicated by formula) and dotted lines show 95% confidence interval.

Supplementary Figure 6

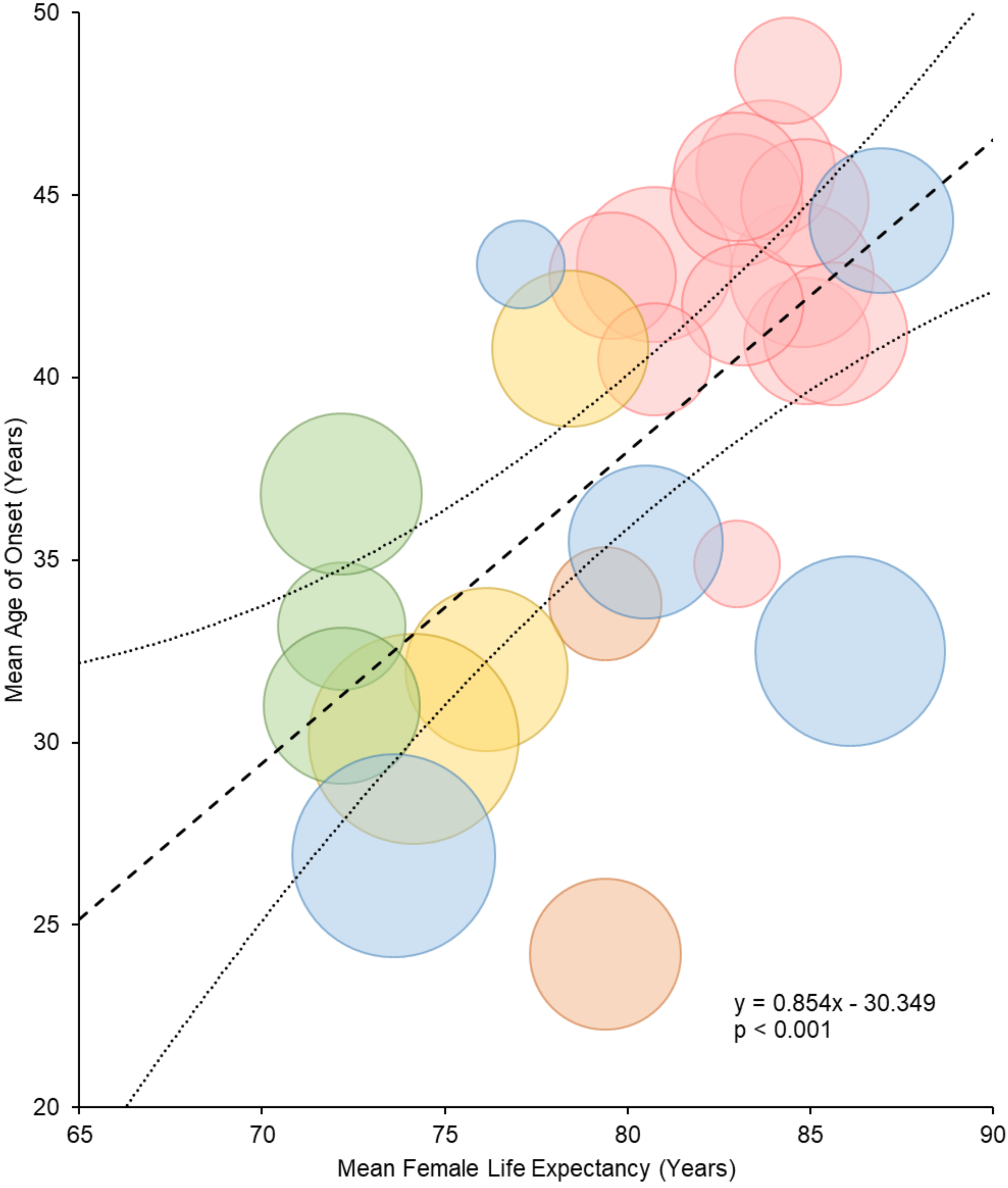

Key

White/European/Caucasian | South/Central American | Middle Eastern/African | South Asian/Indian Subcontinent | East Asian

Bubble plot of mean age of onset against mean female, life expectancy for the country of study, restricted to studies with all AQP4 antibody positive cases. Bubble size is proportional to the variance of the mean age of onset. Meta-regression model included geographical region. Dashed line indicates fitted regression from meta-regression analysis (indicated by formula) and dotted lines show 95% confidence interval.

Supplementary Figure 7

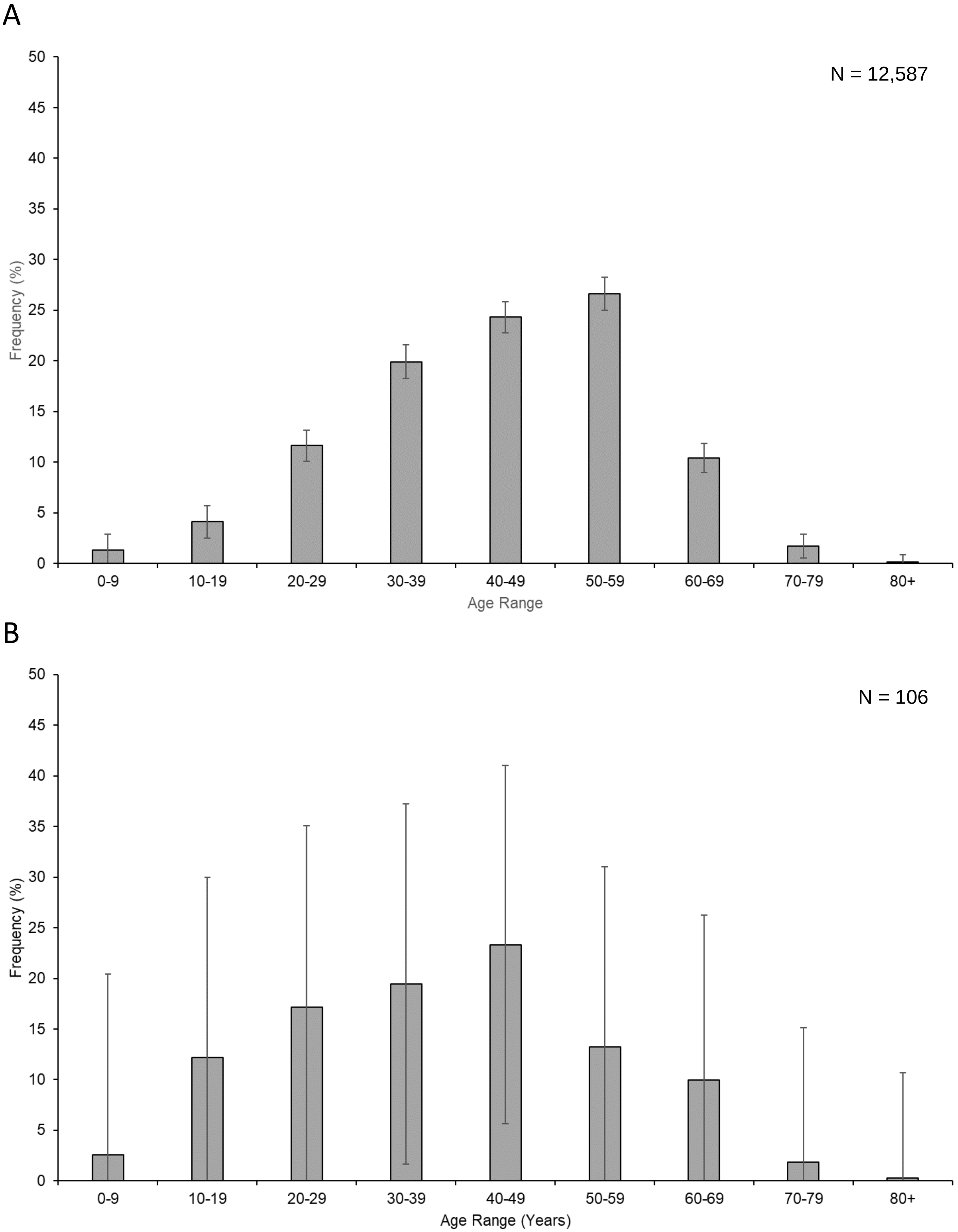

Age of onset distributions for NMOSD adjusted to a flat population structure for all studies (A) or restricted to studies of AQP4 seropositive cases and meeting all JBI critical appraisal criteria (B). Error bars show 95% confidence interval. N = total number of cases.
